# Trade-offs between nature and people in Ethiopia’s protected areas demonstrate challenges in translating global conservation targets into national realities

**DOI:** 10.1038/s41559-026-03047-9

**Published:** 2026-05-12

**Authors:** Sophie Jago, Gebremeskel Gizaw, Bezawit Genanaw, Joe Langley, Ermias Lulekal, Joseph D. M. White, Adèle N. Rowlands, Tariku Geda, Kumara Wakjira, Fekede Regassa, Sebsebe Demissew, Feleke Woldeyes, Wendawek Abebe, Julia P. G. Jones, Robert J. Smith, James S. Borrell

**Affiliations:** 1 Royal Botanic Gardens, Kew, Richmond, UK; 2 Durrell Institute of Conservation and Ecology, University of Kent, Canterbury, UK; 3 Ethiopian Wildlife Conservation Authority, Addis Ababa, Ethiopia; 4 Addis Ababa University, Addis Ababa, Ethiopia; 5 Department of Zoology, University of Cambridge, Cambridge, UK; 6 Independent researcher, Rochester, UK; 7 Ethiopian Biodiversity Institute, Addis Ababa, Ethiopia; 8 The National Herbarium of Ethiopia, College of Natural and Computational Sciences, Addis Ababa University, Addis Ababa, Ethiopia; 9 School of Environmental and Natural Sciences, Bangor University, Bangor, UK; 10 Institute of Environmental Biology, Utrecht University, Utrecht, The Netherlands; 11 Present Address: Department of Biology, Arba Minch University, Arba Minch, Ethiopia

**Keywords:** Conservation biology, Environmental impact, Developing world

## Abstract

Achieving global biodiversity targets, such as the commitment to conserve 30% of the planet by 2030, depends on the ability of individual countries to translate targets into reality. While there has long been recognition that protected areas can bring costs as well as benefits, the implications of this for delivery of global targets have not been fully explored. We focus on Ethiopia, a country supporting globally important biodiversity but facing substantial poverty challenges. We characterize the extent and representativeness of Ethiopia’s protected area network, demonstrating that a threefold expansion—particularly into ecoregions with higher opportunity cost—would be required to meet the Kunming–Montreal Global Biodiversity Framework Target 3. Using a quasi-experimental approach (accounting for known confounders and exploring sensitivity to potential unobserved confounders), we show that the existing protected area network has reduced forest loss and agricultural expansion, and helped to maintain grasslands. Yet, this has brought social wellbeing costs equivalent to 3.9 million fewer household-months of adequate food. Surveys show that national conservation stakeholders recognize these challenges and prioritize improving effectiveness of the existing network over expansion. Our findings highlight that trade-offs between environmental and social outcomes are not simply challenges to be managed, but are central to whether global biodiversity commitments can be delivered.

## Abstract (web summary)

Quasi-experimental analysis of the effects of Ethiopia’s protected areas on environmental and human wellbeing is combined with surveys of conservation professionals to explore why improving protected area effectiveness may be prioritized over network expansion.

## Main

Ambitious global targets provide a shared vision for halting biodiversity loss, but achieving them depends on the ability of individual countries to turn commitments into action^1^. In 2022, 196 parties committed to conserve 30% of the planet by 2030 under the Kunming–Montreal Global Biodiversity Framework (GBF) Target 3 (30-by-30)^2^, a substantial increase from the current terrestrial protected and conserved area coverage of 17.2% (ref. ^3^). While attention has largely been focused on area coverage^4^, both 30-by-30 and its predecessor, Aichi Target 11, also require protected areas to be ecologically representative, well connected, effectively managed and equitably governed^2^—dimensions that are far less often systematically assessed or reported^5^. Evidence from the global south shows that simple ‘win–win’ narratives can be misleading with costs often borne locally, especially by marginalized groups^6^. As the target deadline approaches, understanding what progress is realistically achievable at the national level, and at what cost, is essential.

Protected areas have predominantly been established on land with lower economic value and fewer opportunity costs, rather than in the locations that would yield the greatest benefits for biodiversity conservation^7,8^. As a result, many ecologically important areas remain under-protected. In 2020 only 44.5% of terrestrial ecoregions had reached the 17% coverage target outlined in Aichi Target 11^9^. To meet the more ambitious 30-by-30 target, and ensure ecologically representative networks, countries will need to expand into underrepresented ecoregions, which risks increasing competition with alternative land use such as agriculture. Consequently, trade-offs with local food supplies, local livelihoods and the number of people impacted are likely to increase dramatically^10,11^.

While area-based approaches dominate global conservation policy^12,13^, debates continue over whether protected areas are performing effectively^14,15^. A growing requirement for evidence to inform conservation policy decisions has driven an increase in research using quasi-experimental methods^16,17^. While studies exploring the impacts of protected areas vary in robustness^18^, researchers have applied quasi-experimental designs to evaluate the effectiveness of protected areas across different outcome measures including forest cover^19–23^, agricultural expansion^24^, anthropogenic threats more broadly^25^, species populations^26,27^ and measures of human wellbeing^28–30^. Studies also vary in scale; however, global syntheses pool highly diverse socio-ecological contexts, which can mask heterogeneity in outcomes and limit national policy relevance^25,29,31^.

There is also an ongoing debate about the extent to which conservation successes from protected areas come at the detriment of the wellbeing of local communities^11,32–35^. In low-income countries where rural poverty remains a considerable challenge, protected areas are increasingly expected to contribute to socio-economic development alongside conservation goals, despite environmental and social goals often conflicting with one another^36–38^. In such contexts, there is little credible evidence of sustained positive social outcomes^11^ and transparent evaluation is needed to identify who bears the costs^33,39^. A few studies have explicitly looked at trade-offs between environmental and social outcomes of protected areas; however, many rely on data aggregated across large administrative units^40–45^, limited outcome indicators^31,40–43,46^ or global proxies for development^31^ that are insensitive to local variation, and household-level multidimensional analyses remain rare^47–49^. With 30-by-30 requiring a near-doubling of the global protected and conserved area estate, understanding current effectiveness and trade-offs between environmental and social wellbeing outcomes—through robust analyses that capture multiple components of wellbeing at fine spatial scales—is increasingly urgent^11^. Without a clearer understanding of trade-offs, countries may be reluctant to support protected area expansion that risks harming local communities, or may require additional funding and international support to offset potential negative effects^11^.

Ethiopia is a good example of a country where there is potential for trade-offs between environmental and social wellbeing outcomes^50^. Ethiopia encompasses two global biodiversity hotspots^51^, but also faces long-standing poverty^52^ and food security challenges^53^. Ethiopia is committed to conserving its biodiversity^54^, having ratified the Convention on Biodiversity in 1995 and signed up to meet the GBF targets in 2022. However, its natural resources are facing growing pressures driven by the need for development and improved living standards^55,56^. In 2020, around 18 million people lived within 10 km of a protected area in Ethiopia, and tensions over land use in these areas has been widely documented^57–60^.

Here we provide a comprehensive national-scale evaluation of Ethiopia’s progress towards the multiple dimensions of the 30-by-30 target. We assess the extent of Ethiopia’s protected area network and how well it represents national ecoregions and species. We then apply a robust quasi-experimental approach to assess both environmental (forest, agriculture and grassland cover change) and human wellbeing (change in months of adequate food, dietary diversity and material wellbeing) impacts of Ethiopia’s protected areas. Considering protected areas individually, we then examine predictors of performance across environmental and wellbeing outcomes. Finally, we explore the views of key national stakeholders in conservation policy and practice and consider the alignment of national priorities and global goals. This research highlights the very real challenges faced by those tasked with turning a global commitment into reality.

## Results

### Protected area extent

**Fig. 1:**
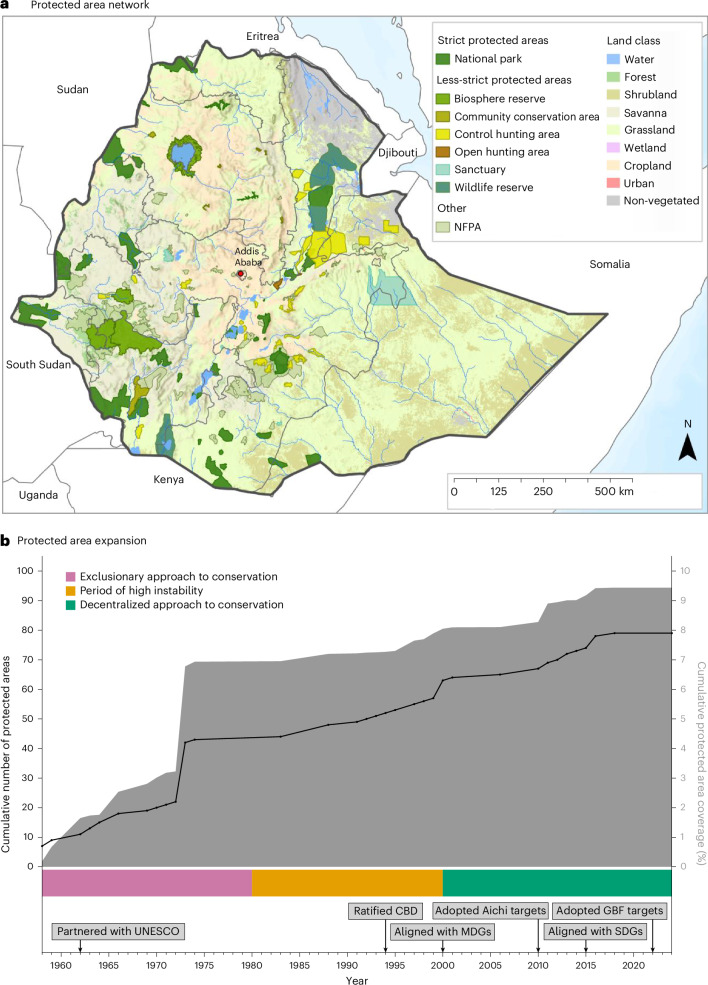
Ethiopia’s protected area network. **a**, Map of Ethiopia’s protected areas indicating the distribution of strict (IUCN category II) and less-strict (IUCN categories IV and VI) protected areas (as of September 2024), coloured by their national designations, overlaid onto a reclassified MODIS V6 land cover map showing hill shade. **b**, The expansion in the number of protected areas and the percentage land coverage of protected areas over time under different overarching approaches to conservation, highlighting major conservation events that have occurred over the timeline. These include Ethiopia’s engagement with international institutions and frameworks, such as UNESCO (the United Nations agency responsible for promoting education, science and cultural heritage conservation) and the Convention on Biological Diversity (CBD), which Ethiopia ratified and subsequently adopted the global biodiversity targets under it including the Aichi Biodiversity Targets and the Global Biodiversity Framework (GBF), as well as alignment with broader global development agendas such as the Millenium Development Goals (MDGs) and Sustainable Development Goals (SDGs). Further information on these time periods is available in Extended Data Fig. 1.

**Extended Data Fig. 1:**
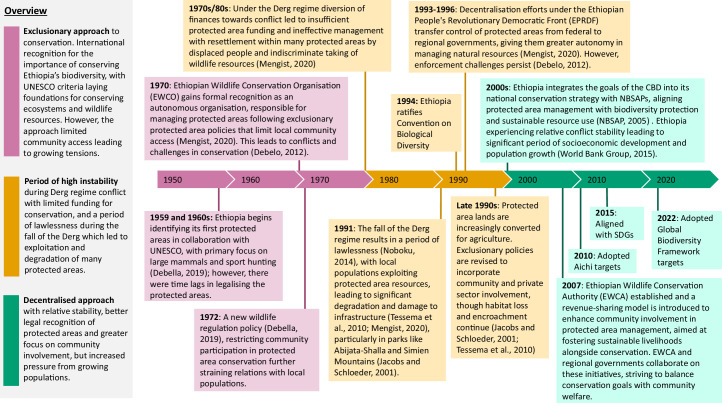
Review of history of conservation in Ethiopia. Ethiopia has been formally establishing protected areas since 1958. Since then, there has been three overarching time periods with different conservation management approaches due to changing national priorities^101,119–121^. From the 1950s to the late 1970s Ethiopia had an exclusionary approach to conservation, limiting community access. From the 1980s to the early 2000s there was a period of high instability with limited funding for conservation during the Derg regime, where protected areas are thought to have been largely ineffective. Since 2000, Ethiopia developed a decentralised approach with better legal recognition of protected areas and greater focus on community involvement, aligning protected area management with sustainable resource use.

As of September 2024, protected areas cover 9.4% of Ethiopia (Fig. 1a). Strict protected areas (International Union for Conservation of Nature (IUCN) category II) make up 3.8% of Ethiopia, while less strict (IUCN categories IV and VI) make up 5.6% (Supplementary Table 10). Including National Forest Priority Areas (NFPAs), which are not protected areas but are included in the World Database on Protected Areas (WDPA), would bring national coverage up to 12.4%. This still differs from the 17% coverage reported in the WDPA (Supplementary Table 1) because our updated dataset removes degazetted or duplicate areas and updates boundaries of downsized or merged areas. Ethiopia’s protected area network has expanded steadily over time (Fig. 1b), amid changing political regimes and evolving conservation policy (Extended Data Fig. 1). Newer protected areas have generally been established in areas of higher human pressures (Supplementary Results 1), and we estimate around 18 million people lived within 10 km of a protected area in 2020.

### Ecological and taxonomic representativeness

**Fig. 2:**
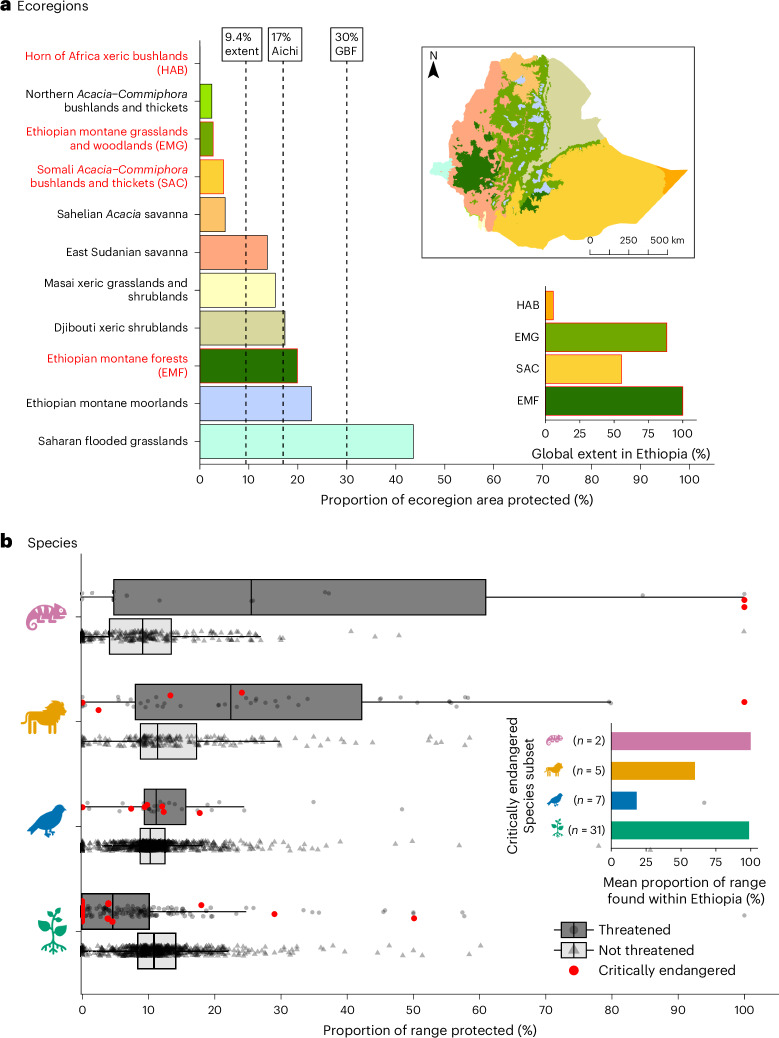
Representativeness of Ethiopia’s protected area network. **a**, Percentage of each ecoregion that is protected, with dashed lines indicating the current total proportion of Ethiopia’s land area protected (9.4%), the 17% Aichi 2020 target and the 30% GBF 2030 target. Terrestrial ecoregions in red are classed as ‘nature imperilled’ by Dinerstein et al.^61^ and the inset graph indicates the proportion of these ecoregions that are found in Ethiopia. **b**, Percentage of range protected for each species, with the spread of this grouped for herptiles (*n* = 241), mammals (*n* = 274), birds (n = 767) and plants (*n* = 785), and separated for threatened (circles; *n* = 294) and non-threatened species (triangles; *n* = 1,773). Boxplots show the median (centre line), the interquartile range (box bounds: 25th–75th percentiles) and whiskers extending to 1.5 × interquartile range. Critically endangered species are shown in red and the inset graph indicates the average proportion of their ranges that are found in Ethiopia.

To be ecologically representative, protected area networks must contain adequate samples of the full range of existing ecoregions, environments and species, especially those that are threatened or are of particular importance^2^. Of the 11 global terrestrial ecoregions present within Ethiopia^61^, 10 are currently represented within the protected area network, but coverage is uneven (0–43%; mean = 13.5%). Four ecoregions exceed the 17% Aichi targets, whereas only one has over 30% in line with 2030 GBF targets (Fig. 2a). Relative to national protected area extent (9.4%), six ecoregions are currently well represented (Fig. 2a). Protected areas also encompassed 33% of Ethiopia’s multidimensional environmental space, defined as the range of climatic and environmental conditions summarized across 19 bioclimatic variables (Supplementary Fig. 5). Gaps in the network disproportionately occur in more accessible areas with more agriculture and higher population densities (Supplementary Results 2).

At the species level, across 2,067 species on the IUCN Red List, Ethiopia’s protected area network covers a higher average proportion of threatened species (*n* = 294) ranges than non-threatened (*n* = 1,773; Fig. 2b). However, threatened plants (*n* = 193) are less well represented with a significantly lower proportion of threatened plant species’ ranges covered by Ethiopia’s protected area network when compared with threatened mammals (*n* = 50, *P* < 0.001), birds (*n* = 36, *P* < 0.001) and herptiles (*n* = 15, *P* = 0.009; Dunn test with Bonferroni correction). Of the 31 critically endangered plant species in this study (30 of which are endemic), 25 are absent from Ethiopia’s protected area network and a further three have less than 5% of their range protected (Fig. 2b). The number of species with their extent of occurrence overlapping each protected area is shown in Supplementary Table 11.

### Protected area effectiveness

**Extended Data Fig. 2:**
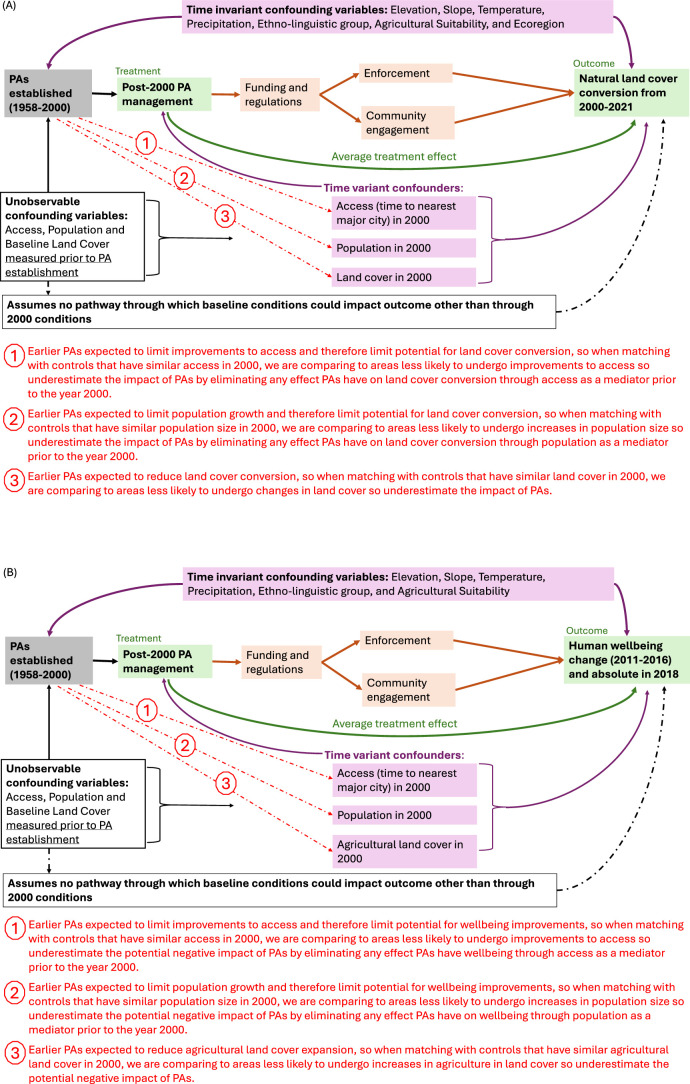
Directed acyclic graphs for the quasi-experimental design. Directed acyclic graphs show the confounding variables controlled for and assumptions made in determining the average treatment effect on the treated for (A) environmental and (B) wellbeing outcomes of protected areas. We use both confounding variables considered to be time invariant and some time variant confounders measured in the year 2000. We use the year 2000 as this represents the time immediately after the period of instability, which we assume acted as a reset for protected areas due to these areas being targeted for exploitation of resources during the Derg regime conflict (Extended Data Fig. 1). While the reset should limit the impact of controlling on covariates in 2000 on our results, we assume that any impact would be in the direction of underestimating rather than overestimating the true impact of protected areas by blocking potential mechanisms through which protected areas may impact land cover change or human wellbeing. This design assumes no hidden confounding variables, we test the assumption of no hidden confounders, allowing us to put bounds on our estimate of the treatment effect of protection.

We used a quasi-experimental approach to assess the effectiveness of Ethiopia’s protected area network across six measures—three environmental outcomes (forest, grassland and agricultural land cover change) and three social wellbeing outcomes (months of adequate food, dietary diversity and material wellbeing)—compared with an estimate of what would have happened if protection had not been put in place (the counterfactual). Using covariate-adjusted regression comparing statistically matched cells and households within and outside Ethiopia’s protected area network, we accounted for key environmental and socio-economic confounders (Extended Data Fig. 2 and Supplementary Table 5), including elevation, slope, temperature, precipitation, agricultural suitability, access to cities, population, ethno-linguistic groups and agriculture. For environmental outcomes we additionally included ecoregion and other baseline land cover variables (forest, grassland, majority land cover type). Descriptive statistics on the changes occurring across Ethiopia for both environmental and wellbeing outcomes, prior to statistical matching, are provided in Supplementary Results 3, with land cover changes shown in Supplementary Fig. 6.

**Fig. 3:**
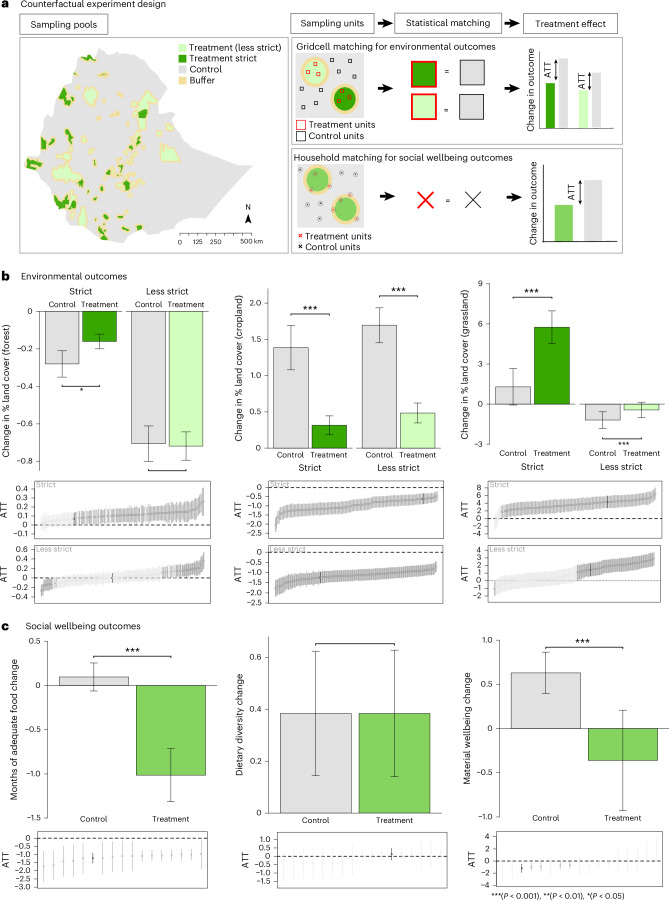
Effectiveness of Ethiopia’s protected area network. **a**, Summary of the counterfactual experiment design. **b**,**c**, Average changes for each effectiveness measure for treatments and controls shown in barcharts for environmental outcomes separately across statistically matched gridcell samples for strict (*n* = 4,702; 2,639 treated and 2,063 control units) and less-strict (*n* = 8,908; 4,454 treated and 4,454 control units) protected area matches (**b**) and for social wellbeing outcomes across statistically matched households (n = 802; 401 treated and 401 control units; **c**). Bars represent mean change in each outcome and error bars indicate a 95% CI calculated across all matched units. ATT values were estimated using covariate-adjusted linear regression on the matched samples, incorporating matching weights and subclass-clustered robust standard errors. Statistical significance of treatment–control differences was assessed using two-sided Wald *z*-tests of the treatment coefficient. For strict protected areas forest cover change, ATT = 0.071 (95% CI: 0.003–0.138), *z* = 2.04, *P* = 0.041; other significant effects had *P* < 0.001. ATTs for each effectiveness measure were then compared with results from 248 different matching specifications for environmental outcomes and 56 for social outcomes, with the main matching approach highlighted in black, other significant results in dark grey and non-significant results in light grey, and error bars showing standard error for the ATT, only models that produced a valid match where the maximum standardized mean difference for covariates was below the 0.25 threshold, and at least 75% of treatment cells were kept. Larger versions of these showing model choices made are available in Supplementary Fig. 7.

**Extended Data Fig. 3:**
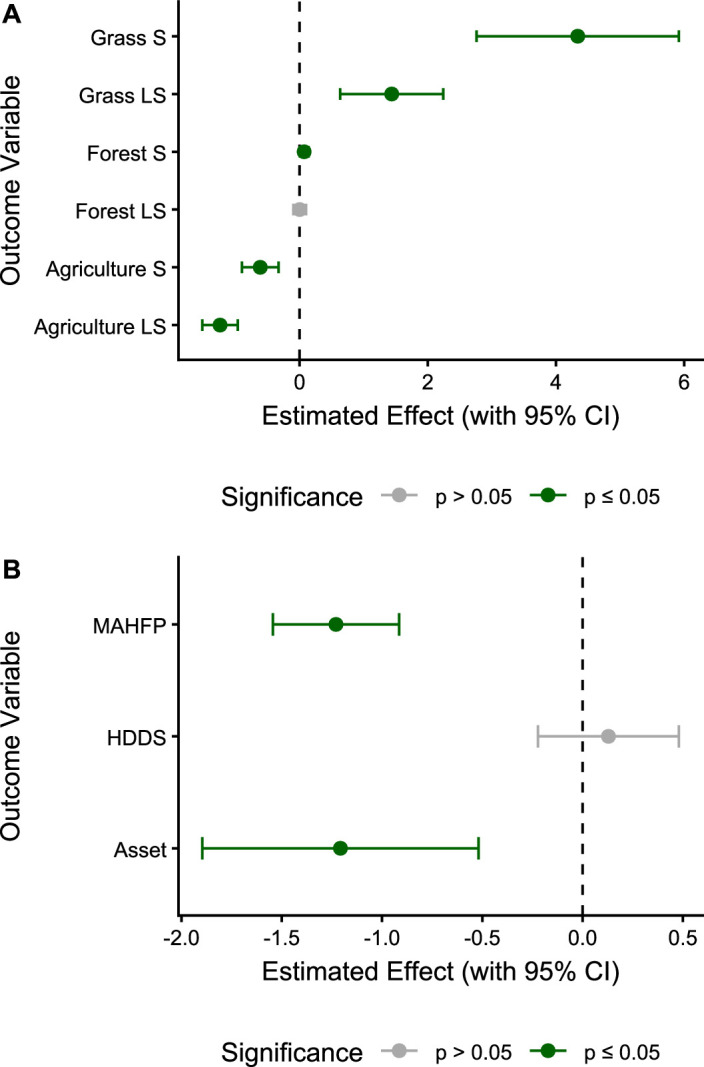
Average Treatment Effects on the Treated (ATT) across Ethiopia’s protected area network. ATT calculated using a covariate adjusted regression model on the matched samples, incorporating matching weights and subclass-clustered robust standard errors for (**A**) environmental outcomes separated into strict (S) and less strict (LS) protected areas, and (**B**) social wellbeing outcomes. For all outcomes except agricultural land cover change, as positive effect indicates the protected areas are performing better than matched controls. Statistical significance of treatment–control differences was assessed using two-sided Wald z-tests of the treatment coefficient. For strict protected areas forest cover change, ATT = 0.071 (95% CI: 0.003–0.138), z = 2.04, p = 0.041; other significant effects had p < 0.001.

Strict protected areas moderately reduced forest cover loss by 25% relative to controls (average treatment effect on the treated (ATT) = 0.07, 95% confidence interval (CI): 0.0003 to 0.14, Wald test statistic (*z*_4675_) = 2.04, *P* = 0.04), equating to approximately 30 km^2^ (CI: 1 to 59) of avoided deforestation. Strict protected areas also significantly reduced agricultural expansion by 44% (ATT = –0.61, 95% CI: –0.90 to –0.33, *z*_4675_ = –4.20, *P* < 0.001), corresponding to 262 km^2^ (CI: 140 to 384) of avoided agricultural expansion, and significantly increased grassland by 76% (ATT = 4.34, 95% CI: 2.76 to 5.91, *z*_4675_ = 5.39, *P* < 0.001), resulting in an additional 1,850 km^2^ (CI: 1178 to 2522) of grassland. Less-strict protected areas showed no significant effect on forest loss (ATT = 0.001, 95% CI: –0.10 to 0.10, *P* = 0.98), but did achieve a 73% reduction in agricultural expansion compared with controls (ATT = –1.24, 95% CI: –1.52 to –0.96, *z*_8884_ = –8.78, *P* < 0.001), equating to 795 km^2^ (CI: 615 to 974) of avoided agricultural expansion, and a 121% reduction in grassland loss (ATT = 1.44, 95% CI: 0.63 to 2.24, *z*_8884_ = 3.5, *P* < 0.001), corresponding to approximately 919 km^2^ (CI: 412 to 1426) less grassland lost (Fig. 3 and Extended Data Fig. 3a).

Although Ethiopia’s protected area network was effective at resisting land cover changes across measured environmental outcomes, this success was associated with substantial local costs for wellbeing. Treatment households close to protected areas experienced a significantly greater decline in perceived months of adequate food, with an average decline of a month compared with almost no change in matched control households (ATT = –1.23, 95% CI: –1.54 to –0.92, *z*_791_ = –7.66, *P* < 0.001). Assuming similar impacts across the 3.2 million households living within 10 km of a protected area in 2011 translates to approximately 3.9 (CI: 2.9 to 4.9) million fewer household-months of adequate food. Material wellbeing, measured as an asset index derived from principal component analysis, also declined significantly for households near protected areas (ATT = –1.21, 95% CI: –1.90 to –0.52, *z*_791_ = –3.44, *P* < 0.001), while it improved in matched control areas. In contrast, there was no significant difference in dietary diversity (ATT = 0.13, 95% CI: –0.22 to 0.48, *z*_791_ = 0.72, *P* = 0.47; Fig. 3 and Extended Data Fig. 3b).

Our results are robust to both unobserved confounders and arbitrary matching choices. Sensitivity analysis using sensemakr showed that, in all cases, an unobserved confounding variable would need to explain more of the residual variance of both the treatment and outcome than is explained by nine times the strength of an observed benchmark covariate, population size for environmental outcomes and agricultural suitability for social outcomes (robustness values for each outcome in each match are reported in Supplementary Table 12). To demonstrate robustness of our results to arbitrary matching choices, we tested 248 different matching model specifications for environmental outcomes, and 56 for wellbeing outcomes. Across valid matching specifications, between 87% and 100% (average 97%) of ATTs were in the same direction as our results for environmental outcomes where we found a significant effect. For human wellbeing outcomes, 100% of ATTs were in the same direction for months of adequate food and 70% for material wellbeing (Supplementary Fig. 7).

### Trade-offs between environmental and social outcomes

**Fig. 4:**
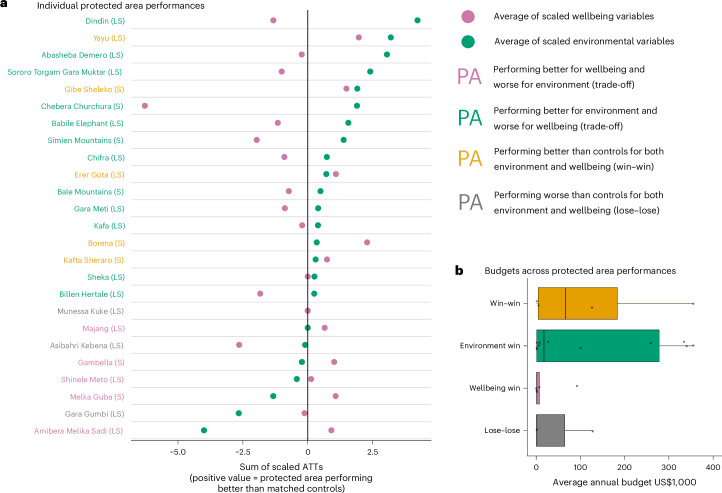
Trade-offs between biodiversity and poverty across Ethiopia’s protected areas. **a**, Sum of the ATT for each protected area across all wellbeing-related variables (pink) and environmental variables (green). Prior to summing each, any non-significant ATTs were set to 0, the ATTs were then divided by the number of years over which they were measured, scaled and transformed such that a positive value indicates better performance than the counterfactual. Protected area (PA) names are coloured according to whether they are performing better for biodiversity (green; *n* = 12) or poverty (pink; *n* = 5), those performing better than the counterfactual for both poverty and biodiversity, that is, win−win outcomes (orange, *n* = 5) and those performing worse than the counterfactual for both, that is, lose−lose (grey; *n* = 3). Brackets after each protected area name indicate if the protected area is strict (S) or less strict (LS). **b**, Spread of average annual budgets, in US dollars scaled to 2014 inflation rates, allocated to protected areas performing at different levels. Only protected areas assessed for both environment and wellbeing outcomes are included here. Boxplots show the median (centre line), the interquartile range (box bounds: 25th–75th percentiles) and whiskers extending to 1.5 × interquartile range.

Of the 25 individual protected areas that we assessed for all six effectiveness measures (a subset limited to those with surveyed households with 10 km), 68% demonstrated trade-offs between environmental and wellbeing outcomes (12 of the 17 protected areas that experienced trade-offs had positive environmental performance at the cost of social wellbeing), 20% experienced win–win outcomes and 12% experienced lose–lose outcomes (Fig. 4a). We report estimated ATTs for individual protected areas for each outcome variable after rebalancing covariates at the individual protected area level using linear model weights (Supplementary Tables 13 and 14, and Supplementary Fig. 8). For wellbeing outcome ATTs, the treated group comprises Living Standards Measurement Study-sampled households located within 10 km of that protected area and is not necessarily representative of protected area level population estimates. Despite the high proportion of protected areas showing trade-offs, environmental performance was not significantly associated with wellbeing performance. Comparing between individual protected areas, full model-averaged estimates (Supplementary Table 15) indicate improved environmental performance was associated with higher area-adjusted budgets (regression coefficient (*β*) = 0.54, *z* = 3.78, *P* < 0.001), less precipitation (*β* = −1.33, *z* = 4.07, *P* < 0.001) and less agricultural suitability (*β* = −0.42, *z* = 2.27, *P* = 0.02). For social wellbeing performance, full model-averaged estimates (Supplementary Table 15) showed no significant associations, the best-supported model included only agricultural suitability and here higher suitability was weakly associated with greater improvements in wellbeing (*β* = 0.68, *t* = 2.06, *P* = 0.051; *R*^2^ = 0.15).

### Stakeholder priorities

While a large increase in protected area coverage would be required to meet the area coverage component of 30-by-30, this is not a priority for stakeholders in Ethiopia. We asked 37 Ethiopian conservation professionals working in policy, research or practice (Supplementary Table 8) to rank three overarching priorities for Ethiopia’s protected area network: (1) expanding the network; (2) making the existing network more effective; and (3) carrying out additional research to guide improvements. Most respondents (77%) ranked effectiveness as their top priority, followed by research, with expansion ranked the lowest. Kendall’s coefficient of concordance indicated significant agreement between participants’ rankings of these priorities (*W* = 0.74, *χ*^2^ = 51.6, *P* < 0.001).

The Ethiopian conservation community recognized issues around protected area effectiveness. The trade-offs found in our analysis align somewhat with stakeholder perceptions of protected area effectiveness, which showed variation across different measures of effectiveness. The number of respondents who selected that protected areas are effective at reducing forest loss and conserving grassland were no different to that expected by chance (*χ*^2^_1_ = 0.03, adjusted *P* = 0.87 and *χ*^2^_1_ = 1.06, adjusted *P* = 0.61, respectively); however significantly more respondents than expected reported that protected areas were not effective at preventing agricultural expansion (*χ*^2^_1_ = 9.76, adjusted *P* = 0.009), reducing poverty (*χ*^2^_1_ = 7.26, adjusted *P* = 0.02) or improving food security (*χ*^2^_1_ = 8.00, adjusted *P* = 0.02).

Weak law enforcement, inadequate community engagement and land-use conflict were the three challenges selected most often by respondents as potentially threatening the effectiveness of Ethiopia’s protected area network. These were selected 22, 19 and 15 times, respectively. In concordance with this, the three actions for improving effectiveness selected most were strengthening policy and law enforcement, strengthening community engagement and enhancing partnerships and collaborations; selected 26, 26 and 20 times, respectively.

## Discussion

International biodiversity targets advocate for dramatic expansion of protected areas^2^. Yet, the sustainability, effectiveness and social acceptance of protected area expansion depends on how much expansion occurs, where it happens and how environmental benefits trade off with social wellbeing impacts. Our study provides one of the most comprehensive assessments of a highly biodiverse country’s progress towards 30-by-30, identifying where expansion would be needed to meet the target and the real-world, context-specific impacts of the existing protected area network.

Given the magnitude of socio-economic and environmental challenges Ethiopia has faced, and the limited resources available in their conservation sector^62^, the success we report of Ethiopia’s protected area network in terms of ecoregion and species representativeness and avoided land-use change is impressive. However, our quasi-experimental analysis provides compelling evidence that Ethiopia’s protected areas are resulting in substantial local trade-offs. While protected areas consistently reduce environmental degradation, they are associated with significantly worse food security and material wellbeing changes among nearby communities (Fig. 3). Only a small subset of Ethiopian protected areas delivered ‘win–win’ outcomes (Fig. 4), often in places where local livelihoods were compatible with conservation (Supplementary Text 1). These findings make an important contribution to ongoing debates about the extent to which protected areas can be expected to deliver ‘win–wins’ in terms of positive impacts on both environmental and social wellbeing outcomes^6,11,29,31,33,34,39^ and provide grounded evidence needed to inform protected area management or expansion decisions.

Ethiopia faces the challenge of meeting ambitious conservation targets while substantial proportions of its population experiences undernourishment and multidimensional poverty^53,63^. In such settings, agricultural development is justifiably a top policy priority^64,65^ that can conflict with conservation goals^66^. For example, while Ethiopia’s protected areas successfully limit agricultural expansion within their boundaries (a conservation gain), without increases in agricultural productivity^67^ this same restriction can exacerbate local food insecurity. With Ethiopia’s population size projected to nearly double from 119 million in 2020 to 225 million people by 2050^68^, and 30-by-30 requiring more than tripling of their current protected area estate, managing these tensions is central to the future of conservation in Ethiopia. Balancing conservation with the urgent needs of a growing and largely agrarian population^69^ will require a shift towards sustainable intensification: producing more food on less land without undermining the resilience of production systems^70,71^. This challenge is not unique to Ethiopia: protected areas conflict with agricultural and grazing land in many parts of the world^72,73^.

Expanding protected areas to pursue ecological representativeness may exacerbate trade-offs between environmental and social wellbeing outcomes. Ethiopia’s underrepresented ecoregions (Fig. 2) are located in areas facing higher human pressures (Supplementary Results 2). For example, the Ethiopian montane grasslands and woodlands represents one of the most agriculturally productive areas in the country^74^, and the Somali *Acacia*–*Commiphora* bushlands and thickets is among one of the most food insecure areas^75^. Expanding protected areas in these regions would probably incur high local opportunity costs, compounding existing livelihood challenges and trade-offs^10,76^. Consistent with this, we find that environmental outcomes of protected areas are worse in wetter and more agriculturally suitable areas where agricultural expansion and timber extraction probably produce better returns^77^, while wellbeing outcomes are marginally better where agricultural suitability is greater. While economic transformation and urbanization may reduce dependence on land-based livelihoods and help ease conservation–livelihood tension over time, future progress towards a more representative network will require careful spatial planning, using multi-objective spatial conservation prioritization tools to identify locations that help to deliver conservation goals at least cost to people^78,79^. Protected areas have often been established in areas with low opportunity costs^7,8^, meaning that many countries—particularly lower-income countries^80^—are likely to face similar challenges when considering ecologically representative protected area expansion.

Chronic underfunding and capacity shortfalls in protected areas around the world^81^ make it difficult to see how dramatic protected area expansion can be achieved in ways that deliver effective conservation without undermining local wellbeing. While we find that higher area-adjusted budgets are associated with improved environmental outcomes, they show no detectable relationship with wellbeing performance. This is consistent with evidence that protected area funding and management capacity (for example, enforcement and habitat management) underpin ecological effectiveness^82^, but that insufficient resources are being allocated to strategies that support livelihood improvements for surrounding communities^11,32,83^. Avoiding negative social impacts of protected areas will require additional, targeted social investments that go beyond core protected area budgets^39^. The Ethiopian protected area network is already severely underfunded^62^. In this context, prioritizing improvements to the existing network over expansion (as suggested by Ethiopian stakeholders) is sensible^84^. Realizing the full potential of Ethiopia’s protected area network will require greater capacity to work with local communities—both to reduce negative livelihood impacts and to unlock the broader opportunities and benefits that conservation can bring^32,34^. Without coordinated action across sectors and stakeholders^66,78,85^, more funding and improved local community involvement, delivering both biodiversity conservation and development goals risks being impossible^71,86,87^.

There are important caveats to our estimates of the impacts of Ethiopia’s protected areas. While Ethiopia’s protected areas were established over a long period, we use the year 2000 as the baseline in our quasi-experimental study design. This is because the year 2000 marks a major turning point, or ‘reset’, in Ethiopia’s political and conservation landscape (Extended Data Fig. 1). Using this baseline allows us to evaluate contemporary protected area performance by aligning the analysis with the governance, budgeting and reporting context under which conservation decisions are currently made, rather than conflating our analysis when conservation operated under a very different political context. This design also allows us to use higher-quality time-variant covariates measured in 2000. Our estimates therefore rely on the assumption that, conditional on the matched covariates, treated and control units would have followed similar trajectories in the absence of protection. Violation of this assumption could bias estimates; however, sensitivity analyses indicate that an unobserved confounder would need to be substantially stronger than the most influential observed covariates to overturn our conclusions. Alternative quasi-experimental approaches^88^ could potentially strengthen internal validity but would substantially restrict the scope for inference. For example, a difference-in-differences design would require protected areas established after 2000, accounting for only around one-quarter of Ethiopia’s protected areas. Restricting the analysis to this subset would not only reduce the sample size but would focus on newer, often smaller protected areas that are unlikely to be representative of the national system. Such an approach would therefore shift the estimand away from the performance of Ethiopia’s protected area network as currently implemented, which is central to national planning under the GBF. Given Ethiopia’s need to balance conservation targets with development priorities at the national scale, we therefore retain a system-wide assessment while transparently acknowledging and empirically testing the assumptions required by the matching design. Finally, our outcomes—land cover change, food security and material wellbeing—reflect where Ethiopia has reliable longitudinal data. As a result, species dynamics and ecosystem-service flows, which may provide broader-scale benefits, are not measured directly. We also note timing mismatches between environmental (2000–2020/21) and wellbeing (2011–2016) outcomes. Although the shorter wellbeing timeframe may miss longer-run effects, it uses the longest household panel available, which allows us to track the same households over time, reduces bias from migration and keeps both outcome sets within the same post-2000 policy regime. These design choices reflect data realities, but provide a transparent, reproducible foundation upon which future work can extend.

Translating global conservation targets, such as the GBF’s 30-by-30 target, into national realities presents substantial challenges that must be navigated across a wide variety of contexts and capacities^89,90^. To bridge the global–local divide, conservation must reflect economic and institutional realities, with governments balancing land-use trade-offs through cross-sector collaboration and inclusive, livelihood-aligned spatial planning. Too often, the benefits of protected areas are realized at much greater regional or global scales, while the costs are borne locally by vulnerable communities^11,33,35^. Ensuring that conservation contributes to local livelihoods and aligns with national development objectives is therefore essential for transforming global ambitions into actionable, equitable outcomes on the ground.

## Methods

### Protected area extent

We collated all Ethiopian protected areas from the WDPA^91^ and then revised these using the most recent information from Ethiopian Wildlife Conservation Authority (EWCA) and cross referenced with IUCN categories. Through this process we added 12 newly gazetted and one missing protected area, and removed 12 degazetted, one duplicated and two that had been amalgamated into other protected areas (the boundaries of which were updated; further details on gazettement years and area changes are provided in Supplementary Table 1). We also exclude from the WDPA database 57 NFPAs, which identify areas with important forest resources but do not meet the IUCN definition of a protected area and often have little natural forest remaining^92,93^. Of these NFPAs, 26 overlap at least partially with gazetted protected areas (Supplementary Fig. 1); for these, we retain the overlapping portions to ensure that all legally recognized protected areas are included. Using the revised dataset and associated metadata, we determined the contemporary area under protection, as of September 2024, and document the historic expansion of the protected area network in relation to Ethiopian conservation history and international targets (Extended Data Fig. 1). We also assessed whether newer protected areas have been established in areas of higher human pressure using Spearman’s rank correlation (Supplementary Methods 1). For year of establishment, we use the earliest record of the protected area either regionally or nationally, as this more closely reflects when on-the-ground protection began, whereas the designation year in the WDPA often refers to later legal updates or IUCN reclassifications.

### Ecological representativeness of Ethiopia’s protected area network

We assessed the percentage overlap of the protected area network across ecoregions using the RESOLVE terrestrial ecoregions dataset^61^ and compare this with the 30% GBF target (for 2030), 17% Aichi target (should have been achieved in 2020) and the current protected area extent. To highlight ecoregions that are of particular importance to be conserved within Ethiopia, we also identified ecoregions that Dinerstein et al. class as ‘nature imperilled’^61^ and calculate the proportion of their global extent that is found in Ethiopia. We then compare human and land-use pressures across ecoregions relative to their representation in protected areas (Supplementary Methods 2). Representativeness of Ethiopia’s protected area network in environmental space, defined as the range of climatic and environmental conditions across the country summarized through a principal component analysis of 19 bioclimatic variables, was also assessed (Supplementary Methods 3).

To assess species representation, we used IUCN Red List range data to calculate the proportion of each species’ range covered by protected areas. Birds, mammals and herptiles (amphibians and reptiles) have been widely assessed on the Red List, whereas vascular plants are comparatively under-evaluated^94^ and many lack IUCN Red List range data. We therefore created range estimates for assessed plant species that did not have range data on the IUCN Red List, using occurrence records (Supplementary Methods 4). This resulted in range data for 2,067 species (785 plants, 767 birds, 274 mammals and 241 herptiles). We determined the average proportion of range protected across taxonomic groups, separately for threatened (*n* = 294) and non-threatened (*n* = 1,773) species and used Kruskal–Wallis and post-hoc Dunn tests with Bonferroni correction to investigate how protected area coverage varied across taxa and threat status categories. For all critically endangered species assessed (*n* = 45), we also calculated the proportion of their global extent within Ethiopia, to highlight global priorities for conservation in Ethiopia. The species’ ranges were then used to estimate the number of species expected to occur within each of Ethiopia’s protected areas.

### Effectiveness of Ethiopia’s protected area network during the period 2000–2020

#### Outcomes

Here, we are interested in evaluating the effectiveness of protected area management since 2000 under Ethiopia’s current approach to conservation (Extended Data Fig. 1). We examined effectiveness of protected areas for both environmental and social wellbeing outcomes across a suite of six proxy indicators. Environmental outcomes included changes over time in forest (2000–2021), grassland (2000–2020) and agricultural (2000–2019) land cover (Supplementary Table 2 and Supplementary Methods 5). These were all measured as the change in percentage land cover using publicly available global remote sensing panel datasets aggregated at 1-km resolution across Ethiopia (time series based on data availability). Sankey diagrams (Supplementary Methods 6) showing overall changes inside and outside protected areas were produced using the MODIS Land Cover dataset^95^.

Wellbeing outcomes were changes from 2011 to 2016 for two indicators of food security—months of adequate household food provisioning (months of adequate food) and household dietary diversity status (dietary diversity)—and one indicator of material wellbeing (asset ownership; Supplementary Tables 2–4). Wellbeing outcomes were derived from the Living Standards Measurement Study Ethiopian Socio-economic Survey, a household-level panel survey where households were first visited in 2011/2012 and revisited in 2015/2016 with attrition, resulting in 3,699 households^96,97^. Measuring change using the panel data reduces bias from people immigrating and emigrating from an area; however, it does not fully eliminate bias due to attrition (Supplementary Methods 7).

To ensure we are not measuring changes in outcomes prior to protected area establishment and that included protected areas existed throughout the outcome measurement period, we excluded from the analysis protected areas established after 2000 (63 of 79 protected areas remained in the analysis). NFPAs (in the WDPA but not considered protected areas) were also analysed separately for forest cover outcomes (Supplementary Methods 8)

#### Quasi-experimental design

To estimate a causal effect of protection on the outcomes of interest we need a credible estimate of the counterfactual: what would have happened in areas had they not been designated as protected. Given protected areas are not randomly assigned in a landscape, we use a quasi-experimental matching design (further justification provided in Supplementary Methods 9), which controls for observed confounding variables likely to affect both exposure to the treatment (being protected during the period 2000–2020) and the outcome (the change in each indictor)^88,98^. Focusing the analysis on the post-2000 period aligns it with the policy context in which decisions are currently made. By assuming that there are no important unobserved confounders we can estimate the treatment effect of protection. We test the sensitivity to the assumption of no hidden confounders^99,100^, allowing us to put bounds on our estimate of the treatment effect of protection.

Directed acyclic graphs were used to visually represent and better understand the variables influencing exposure to the treatment and links to the outcomes of interest, and therefore to identify confounding variables that should be controlled for to isolate the treatment effect of protected area status (Extended Data Fig. 2). We match on confounders presumed to be time-invariant including elevation, slope, precipitation, temperature, agricultural suitability, ethno-linguistic group and ecoregion (Supplementary Tables 5 and 6). These variables are included specifically to reduce bias due to confounding. We also match on some additional time-variant covariates measured in 2000 (after protected area establishment but prior to our outcome measures): access, population, percentage forest cover, percentage grassland cover, percentage agricultural land cover and majority land cover type (Supplementary Tables 5 and 6). These variables were included to improve our estimates by accounting for additional variation in the outcomes. We use the year 2000 as this represents the period following high instability under the Derg regime and the subsequent targeted exploitation of protected areas after its fall, during which there was limited funding for conservation^101^ (Extended Data Fig. 1). This period effectively acted as a reset for protected area management in Ethiopia, before the relatively more stable post-2000 period where management has been more aligned with the goals of the Convention on Biological Diversity. While the reset should limit the impact of controlling on covariates measured in 2000 on our results, we assume that any impact would be in the direction of underestimating rather than overestimating the true impact of protected areas by blocking potential mechanisms through which protected areas may impact land cover change or human wellbeing^18^; further details on these assumptions are provided in Extended Data Fig. 2. We also test whether our results are driven by this assumption by iteratively excluding these covariates in alternative matching approaches (see ‘Sensitivity checks’).

#### Units of assessment

For environmental outcomes, data for covariates and outcomes were aggregated across each 1-km sampling unit^102^. Treatment units comprised gridcells completely within protected area boundaries and were categorized into two classes: strict (IUCN category II) and less strict (biosphere reserves and IUCN categories IV and VI). Protected areas in IUCN categories Ia, Ib III and V, and Other Effective area-based Conservation Measures, are not present in Ethiopia. We excluded gridcells that intersected a 10-km buffer zone around each protected area to avoid underestimating effects due to local leakage^20,103^. The remaining gridcells outside both protected areas and buffer zones were classified as potential control units. Using a gridded sampling technique, we checked a range of sampling densities (Supplementary Table 7) to identify the closest distance between gridcells that did not show spatial autocorrelation (2 km between each cell). We then sampled gridcells using a gridded sampling technique that ensured each gridcell was 2 km from another gridcell, and checked for spatial autocorrelation in treatment units using semi-variograms^103^.

For wellbeing outcomes, owing to household coordinates being randomly offset by 0–2 km to maintain participant confidentiality (Supplementary Methods 10), covariate data were aggregated across a 2-km buffer around each household unit. The sampling unit was individual households, and we compared households living near or within protected areas with those unaffected by protected areas. Households were classified as treatment units if their 2-km buffer overlapped a 10-km buffer zone around a protected area. Households further than 20 km from a protected area were classified as control units, ensuring controls were at least 10 km further from protected areas than treatment units. A map showing the locations of survey enumeration areas is provided in Supplementary Fig. 2.

#### Statistical matching

Assessing the effectiveness of protected areas by comparing them with unprotected areas is likely to produce biased results^19^. Effectiveness assessments that use statistical matching can help to overcome this spatial bias^104^ by selecting control units (for example, unprotected areas) that have similar baseline characteristics to the units experiencing treatment (for example, protected areas)^88,105^. Following ref. ^103^, we iteratively tested several matching methods and compared the resulting match quality before the deciding on the main matching specification using the R package MatchIt^106^. The modelling choices included variations of propensity score nearest neighbour matching and Mahalanobis distance matching with and without calipers and replacement. All models tested used exact matching for categorical covariates (ecoregion and majority land cover type). The quality of matches were compared to determine the best matching approach based on the proportion of treated units that were matched and the covariate balance achieved (using a threshold standardized mean difference of 0.25; refs. ^103,107^). Love plots showing the balance achieved across covariates (as the standardized mean difference between treatment and control samples) for each matching model choice tested are shown in Supplementary Fig. 3. The best match for environmental outcomes for strict treatment samples was nearest neighbour propensity score matching with 0.5 standard deviation calipers and replacement, which retained 93% of treatment units and a maximum standardized mean difference of 0.16. For less strict, the best match was Mahalanobis distance matching without replacement, which retained 98% of treated units and a maximum standardized mean difference of 0.13. For household outcomes, the best match was nearest neighbour propensity score matching with 1 standard deviation calipers without replacement, this retained 75% of treatment units and a maximum standardized mean difference of 0.11. Comparisons of pre- and post-match boxplots demonstrate the reduced variance of covariates between treatment and control units achieved through matching (Supplementary Fig. 4).

#### Treatment effect

Using the three matched datasets (strict protected areas, less-strict protected areas and households across all protected areas), we estimate the ATT for each outcome using a covariate-adjusted regression model. This represents the average difference in the change in each outcome between matched treated and control units, after adjusting for covariates. By combining both matching and regression adjustment, we obtain more accurate and robust estimates than either matching or regression alone^108^. We applied weights from the matching procedure and clustered by subclass according to the matched data structure to calculate robust standard errors^109^. Statistical significance was determined using two-sided Wald *z*-tests of the treatment coefficient. For all outcome variables (except change in agricultural land), a positive ATT would indicate that protected areas are performing better than matched controls. We converted ATTs into relative percentage changes by dividing each ATT by the mean change in the control group, to report the proportional effect of protection relative to expected land cover change in the absence of protection. Finally, for environmental outcomes, we estimated the total area of avoided loss attributable to protection by multiplying the ATT by the total treated area. Likewise, to estimate the aggregate social effect of protected areas on local communities, we multiplied the ATT for social outcomes by the estimated total number of households living within 10 km of a protected area in 2011 (calculated using gridded population estimates and the average household size of surveyed households; see Supplementary Methods 11).

#### Sensitivity checks

The sensitivity of the results to hidden bias due to the presence of unobserved confounding variables was assessed^100^ with the R package sensemakr^110^. This approach identifies the proportion of residual variance of both the treatment and the outcome that would need to be explained by an unobserved confounder to nullify the treatment effect, and compares this with the strength of a benchmark observed covariate^99^.

To provide further validation, we compared our estimates of the ATT with results from 248 alternative model specifications for strict and less-strict matching, and 56 for household matching^111,112^, to confirm whether they are robust to arbitrary modelling choices. Comparison models differed in the combination of covariates used, keeping all time-invariant confounders and cycling though different combinations of the covariates measured in 2000, the distance measure (propensity score matching or Mahalanobis), caliper sizes (0.25, 0.5 or 1 standard deviation) and whether replacement was allowed or not.

### Identifying trade-offs between environmental and human wellbeing outcomes

Treatment units for individual protected areas were extracted from the matched datasets and covariates were rebalanced against the control units using linear model weights using the R package lmw^113^. This weights the data to achieve approximate balance between covariates across treatment and control units, using a uniform risk increase weighting method. Weighted outcome models were estimated using the lmw_est() function and ATTs were calculated for individual protected areas as the difference in the weighted means for each outcome variable between treatment and control groups.

To evaluate the trade-offs between environmental and wellbeing outcomes, we set non-significant ATTs to zero and scaled significant ATTs for each outcome variable, with negative values indicating that protected areas performed worse than their matched controls and positive values indicating that protected areas performed better (the ATTs for agricultural land change were inverted to aid interpretation). The scaled values for the three environmental variables and three wellbeing variables were then summed to produce single environmental performance and wellbeing performance value, and we identify which protected areas perform worse than the counterfactual for both environmental and wellbeing outcomes (lose–lose), experience trade-offs (win–lose) or perform better for both (win–win).

We assessed correlates of variation in protected area performance using data on main ecoregions, management strictness and non-governmental organization involvement (Supplementary Table 8) as well as average temperature, precipitation, elevation, agricultural suitability, agricultural land cover, accessibility (data sources in Supplementary Table 5) and area-adjusted budgets. Protected area budget data (Fig. 4b) were obtained from the EWCA as average annual budgets (in US dollars adjusted to 2014 inflation levels). To account for nonlinear scaling of costs across protected area sizes^114,115^, we modelled budget as a function of area and used the residuals as an area-adjusted measure of financial input. Continuous predictors were standardized, and variables with high multicollinearity (variance inflation factor >5) were excluded. We then modelled both environmental and wellbeing performance outcomes separately to maintain as much data as possible (as fewer protected areas were assessed for wellbeing outcomes, due to not all protected areas having households surveyed in the Living Standards Measurement Survey). For each outcome, we fitted linear mixed-effects models with ecoregion as a random intercept and compared them with fixed-effects models using Akaike information criterion (AIC) and likelihood ratio tests. Using the better fitted base model, we then performed model selection and averaging with the package MuMIn^116^, ranking models by AIC and averaging across all models with AIC < 2. Residuals were examined for normality and homoscedasticity. Analyses examining correlates of variation in protected area performance are intended as exploratory, as ATT estimates are themselves subject to uncertainty that is not fully propagated into second-stage models.

### Understanding priorities of Ethiopian conservation practitioners

We surveyed Ethiopian conservation researchers, practitioners and policymakers on the priorities and challenges in making progress towards 30-by-30, as well as their perceptions of protected area effectiveness (Supplementary Methods 12; approved by the University of Kent Conservation Ethics Committee: Ethics ID 20251741251220900). We specifically targeted those working directly or indirectly in protected area policy, management or research using a purposive, opportunistic, snowball sampling approach^117^. Participation was voluntary and informed consent was obtained from all respondents prior to data collection. We obtained 37 responses from stakeholders representing non-governmental organizations, private companies and research institutes/universities, with the majority in governmental bodies. The largest proportion of respondents were aged 31–40 (41%), male (86%) and educated to Masters level (57%; Supplementary Table 9). These characteristics reflect the demographic and professional composition of the sampled conservation practitioner community rather than the general population. We used Kendall’s coefficient of concordance, using the R package irr^118^, to assess levels of rank-order agreement for prioritizing overarching goals for Ethiopia’s protected area network; and chi-squared tests (with Holm–Bonferroni correction) to determine overall perceptions of success for each measure of effectiveness. All analyses were conducted in R version 4.2.1.

### Reporting summary

Further information on research design is available in the Nature Portfolio Reporting Summary linked to this article.

## Supplementary information

Supplementary Information Supplementary Methods 1–12, Supplementary Results 1–4, Supplementary Text 1, Supplementary Figs. 1–10 and Supplementary Tables 1–16.

Reporting Summary

Peer Review File

## Data Availability

Updated protected area shapefiles are available via GitHub at https://github.com/SCJago/protected_area_performance/tree/main/data. Protected area budget data are available in categorical format in Supplementary Table 9; continuous numerical budget data requests should be directed to the Ethiopian Wildlife Conservation Authority. Species’ range data are available for download from the IUCN Red List, either via a manual search (https://www.iucnredlist.org/search) or through the spatial database (https://www.iucnredlist.org/resources/spatial-data-download). Point data for plant species without IUCN Red List ranges can also be obtained from the same sources. Occurrence data for Ethiopia’s endemic plant species are stored in the Endemic Plants of Ethiopia database on RBG Kew BRAHMS Online. BRAHMS data requests should be directed to the relevant contact listed on this webpage: https://brahmsonline.kew.org/kewbol/Websites. All open source datasets used are referenced in the Article or its Supplementary Information.

## Code availability

Code used in this manuscript is available via GitHub at https://github.com/SCJago/protected_area_performance/tree/main/scripts.
