# Supplementary material for: Trade-offs between nature and people in Ethiopia’s protected areas demonstrate challenges in translating global conservation targets into national realities
Source: Nat Ecol Evol. 2026 May 12;10(6):1057–70. doi: 10.1038/s41559-026-03047-9 (PMC13253355; doi:10.1038/s41559-026-03047-9)
Supplement: Supplementary file 1 — Supplementary Methods 1–12, Supplementary Results 1–4, Supplementary Text 1, Supplementary Figs. 1–10 and Supplementary Tables 1–16. [file 41559_2026_3047_MOESM1_ESM.pdf]

nature ecology & evolution

Supplementary information https://doi.org/10.1038/s41559-026-03047-9

Trade-offs between nature and people in Ethiopia's protected areas demonstrate challenges in translating global conservation targets into national realities

In the format provided by the authors and unedited

Contents

Supplementary methods.. 4

Supplementary Methods 1 Assessing whether newer protected areas were established in areas of higher human pressure 4

Supplementary Methods 2 Assessing whether expanding protection into underrepresented ecoregions would result in greater human and land-use pressures.. 4

Supplementary Methods 3 Representativeness of Ethiopia's protected area network in multidimensional environmental space.. 4

Supplementary Methods 4 Generating plant species ranges.. 5

Supplementary Methods 5 Understanding grassland changes 5

Supplementary Methods 6 Sankey landcover changes 6

Supplementary Methods 7 Socio-economic survey household attrition 6

Supplementary Methods 8 National Forest Priority Area counterfactual analysis 6

Supplementary Methods 9 Justification for the use of matching methods 6

Supplementary Methods 10 Socio-economic survey household offset information 7

Supplementary Methods 11 Estimating impacted populations 7

Supplementary Methods 12 Stakeholder questionnaire.. 7

Supplementary results 12

Supplementary Results 1 Newer protected areas are being established in areas of higher human pressure 12

Supplementary Results 2 Human and land-use pressures across under- and over- represented ecoregions.......... 12

Supplementary Results 3 Overall land cover changes.. 12

Supplementary Results 4 Interpreting grassland changes.. 12

Supplementary text 14

Supplementary Text 1 Context for win-win sites.. 14

Supplementary figures.. 15

Supplementary Figure 1 The overlap of National Forest Priority Areas (NFPAs) with gazetted protected areas .. 15

Supplementary Figure 2 Map of enumeration area locations from households surveyed in both the 2011 and 2016 Living Standards Measurement Survey[29,30] (333 enumeration areas comprising 3917 households). The control pool (2560 households) are more than 20km from a protected area, treatment pools (721 households) are less than 10km from a protected area and buffer pools (636 households) are in between. 16

Supplementary Figure 3 Comparison of statistical matching specifications. Comparing the covariate balance achieved and sample unit retention (shown in in the legend) across six statistical matching specifications for (A) statistical matching for strict protected areas, (B) statistical matching for less strict, and (C) household matching. Different coloured points represent different matching specification. The matching specifications selected for further analysis were the specifications with the highest sample unit retention which achieved covariate balance below the threshold of 0.25 (dashed line). PSM = propensity score matching, NN = nearest neighbour. 18

Supplementary Figure 4 Comparison of treatment (green boxes) and control (grey boxes) covariate values for (A) strict pre-match data, (B) strict post-match data, (C) less strict pre-match data, (D) less strict post-match data, (E) household pre-match data and (F) household post-match data.. 19

Supplementary Figure 5 Representativeness of Ethiopia's environmental space within the protected area network. (A) shows a principal component analysis plotting Ethiopia's background environmental space (grey) and the environmental conditions found within the protected area network (green), with alpha hulls where alpha is set

to 0.3. 33% of the Ethiopia's environmental space matched the environment found within protected areas. (B) shows the PCA converted into geographical space, where green highlights areas which match the environmental conditions already found within the protected area network. Variable loadings for the first and second principal component axes are shown in (C) and (D) respectively, where dashed lines represent the value expected if all contributions were uniform. 20

Supplementary Figure 6 Landcover changes in each 1x1km gridcell that occurred between 2000 and 2021 (A) inside PAs, (B) outside PAs, (C) inside PAs excluding gridcells where the landcover remained constant and (D) outside PAs excluding gridcells where the landcover remained constant. 21

Supplementary Figure 7 Comparing results from the primary matching approach to 248 different matching model specifications for each matching treatment and corresponding outcome variables (A-I). The result from the primary matching approach is shown in black. For all comparison models, those where there was a significant difference between treatments and controls are shown in dark grey and those with no significant difference are shown in light grey. Average Treatment Effect on the Treated (ATT) were estimated using covariate-adjusted linear regression on the matched samples, incorporating matching weights and subclass-clustered robust standard errors. Statistical significance of treatment–control differences was assessed using two-sided Wald z-tests of the treatment coefficient. Results from models which did not achieve sufficient balance (standardised mean difference for all covariates <0.25) or sample size (at least 75% of treatment units matched) during matching were removed from the figures to reduce noise. 26

Supplementary Figure 8 Maps showing the performance of each protected area compared to matched controls. A protected area is found to be performing better than matched controls if its average treatment effect (ATT) is significantly positive (agriculture ATTs were inverted to conform with this), and worse if the ATT is significantly negative 27

Supplementary Figure 9 National Forest Priority Area (NFPA) counterfactual analysis comparing forest loss from 2000-2021 within NFPAs and in matched control areas outside of both NFPAs and protected areas 28

Supplementary Figure 10 Sankey diagrams showing landcover changes from and to grassland (2001-2020) in statistically matched units (A) treatment units for strict PAs, (B) control units for strict PAs, (C) treatment units for less strict PAs, and (D) control units for less strict PAs. Gridcells which remained grassland and gridcells which changed between land cover types that were not grassland were excluded for easier interpretation. 29

Supplementary tables 30

Supplementary Table 1 List of protected areas removed and added cleaning the World Database of Protected Areas dataset for Ethiopia with updated information from the Ethiopian Wildlife Conservation Authority, along with their metadata and the reason for the change. 30

Supplementary Table 2 Measures of effectiveness. Table showing the three environmental (forest, grassland and agricultural land cover change) and three social wellbeing (months of adequate food, dietary diversity, material wellbeing) outcome variables used to understand protected area effectiveness. All outcome variables were calculated as a change over their corresponding time period. Expectation refers to the expected direction of change for PAs compared to matched counterfactuals in the case the PAs are effective. 31

Supplementary Table 3 Food items considered in determining household dietary diversity status 32

Supplementary Table 4 Asset items for calculating material wellbeing 32

Supplementary Table 5 Covariates used for statistical matching relating to each directed acyclic graph (DAG) in Extended Data Fig 2 A and B. Data for all covariates resampled to 1km resolution for gridcell matches and to a 2km buffer around each household for household matches. Further information on the theory for including each covariate is included in Supplementary Table 6. 33

Supplementary Table 6 Theory on how each confounding variable may influence both treatment and outcomes from literature review focused on the Ethiopian context. Darker colours indicate stronger expected impact on the outcome. 35

Supplementary Table 7 Semivariance produced at different sampling densities for gridcells 37

Supplementary Table 8 Involvement of external project funding or non-governmental organisations (NGOs) with protected areas. This compiles accessible information available for each protected area on whether it was associated with any external funding between 2000 and 2020. 38

Supplementary Table 9 Demographic information for questionnaire respondents. N is number of respondents and % is the percentage of respondents. 40

Supplementary Table 10 Updated full list of protected areas and associated metadata. Earliest year represents the earliest record (to the best of the Ethiopian Wildlife Conservation Authorities knowledge) of the protected area either regionally or nationally, including if the area used to be under a different type of protection or different name, rather than the date it was designated on the World Database of Protected Areas. Budget group indicates whether the budget fell within the bottom quartile (Low), interquartile range (Mid) or upper quartile (High). True budget data can be obtained from the Ethiopian Wildlife Conservation Authority. This dataset represents the protected area shapefile in September 2024, this is continually being updated. 41

Supplementary Table 11 Number of species with range overlapping each protected area 43

Supplementary Table 12 Matching is robust to the presence of an unobserved confounding variable. Outputs from sensitivity analyses conducted using the R package *Sensemakr* for each outcome variable in each matching group. The robustness value (RV) represents the percentage of the residual variance of both the treatment and the outcome that an unobserved confounder would need to explain to bring the estimated effect to zero. This is then compared to a benchmark covariate, for environmental outcomes we used population size as the benchmark, and for social outcomes we used agricultural suitability. We only tested these bounds up to 9 times the strength of the benchmark. 45

Supplementary Table 13 Individual protected area environmental outputs showing the average treatment effect on the treated (ATT), t-statistic (t) and significance (p); significant p-values are shown in bold. For forest and grassland, a positive ATT indicates better performance while for agriculture a negative ATT indicates better performance. 46

Supplementary Table 14 Individual protected area social wellbeing outputs showing the average treatment effect on the treated (ATT), t-statistic (t) and significance (p); significant p-values are shown in bold. A positive ATT indicates better performance. 48

Supplementary Table 15 Model averaged predictors of protected area environmental and wellbeing performance across models where (ΔAIC < 2) demonstrate significant predictors for environmental performance are area-adjusted budget, precipitation and agricultural suitability, while for wellbeing performance none are significant. 49

Supplementary Table 16 Changes from grassland to savanna and shrubland are associated with bush encroachment. Results from a linear regression with cattle, sheep and goat densities as predictors of cells which change from grassland to savanna or shrubland (Adjusted $R^2 = 0.006$, $F_{(3, 4836)} = 4.12$, $p = 0.006$) 49

Supplementary references 50

Supplementary methods

Supplementary Methods 1 Assessing whether newer protected areas were established in areas of higher human pressure

The protected area network was divided into 1km gridcells and sampled such that we maintained 10% of the gridcells in each protected area and these were a minimum of 2km apart. We then extracted variables assumed to indicate human pressure including population[1], agricultural land cover[2], accessibility[3], and elevation[4]. We used the year 2000 for time variant variables to avoid confounding the results by population growth or development over time. Relationships between protected area year of establishment and each human pressure variable were assed using Spearman's rank correlation. Correlation coefficients and associated p-values were reported to indicate the strength and significance of relationships.

Supplementary Methods 2 Assessing whether expanding protection into underrepresented ecoregions would result in greater human and land-use pressures

We classified each ecoregion as either under-represented or over-represented based whether the proportion of its extent within protected areas was below or above 9.4%, respectively (9.4% represents current protected area land coverage in Ethiopia. We then sampled 250 points within each group and the number of points allocated to each ecoregion was proportional to ecoregion area multiplied by its deviation from the 9.4% benchmark. At each sampled point, we extracted variables indicative of potential pressures including population[1], agricultural land cover[2], agricultural suitability, accessibility[3], and elevation[4]. To compare the groups while respecting the spatial structure, we used blocked permutation test using the R package *coin*, which repeatedly shuffles group labels while preserving local spatial structure to generate a null distribution. We used 999 permutations and report two-sided p-values with Benjamini–Hochberg FDR correction.

Supplementary Methods 3 Representativeness of Ethiopia's protected area network in multidimensional environmental space

A principal component analysis (PCA) was performed on 19 environmental variables from CHELSA Bioclim [5] to plot 100,000 random points sampled from across Ethiopia into the environmental space defined by these variables. Additionally, we sampled 9400 random points from within Ethiopia's protected area boundaries. Sample sizes were proportional to the extent of Ethiopia's protected area network. The protected area points were projected into multidimensional space using the *predict* function and overlaid onto the PCA plot to visualise the distribution of protection across Ethiopia's environmental space. Using the R package *alphahull*, we then delineated flexible boundaries around the plotted points. Multiple values of alpha were visually inspected to find the best compromise between overfitting and underfitting. We set the alpha of both the background and protected area network's environmental space to 0.3 and calculated the area of each's hull. The percentage of Ethiopia's environmental space represented within the protected area network was then calculated. Variable loadings of the first and second principal components are reported in Supplementary Figures S5C and S5D, respectively. The coordinates of the points where then plotted in geographical space to identify the locations containing underrepresented environmental conditions.

Supplementary Methods 4 Generating plant species ranges

Using occurrence records from the IUCN Red List and the Royal Botanic Gardens Kew's Botanical Research And Herbarium Management System [6] database, we created range estimates for plant species that did not have range data on the IUCN Red List. For species with three or more occurrence records, we used the subLocRapoport function from the rCAT package to generate ranges and used the default buffer width (mean branch length of the Euclidean Minimum spanning tree). For species with 1 or 2 occurrence records, we manually assigned a 5 km buffer to each point.

Supplementary Methods 5 Understanding grassland changes

Using the statistically matched units (1km gridcells), we produced sankey diagrams highlighting what land cover grassland had changed from or to. Units where grassland remained grassland or where changes did not involve grassland were excluded from the diagrams. Sankey diagrams are displayed separately for strict and less strict PAs and their respective matched controls. Where grassland changed to savanna or shrubland, it was important to identify whether this was due to bush encroachment (a negative impact) or savanna recovery (a positive impact). Many of Ethiopia's ecoregions are classified as containing more than one land cover type (e.g. Ethiopian montane grasslands and woodlands or Ethiopian xeric grasslands and shrublands) making it challenging to determine which natural baseline one would target without archaeobotanical evidence. Bush encroachment may be a consequence of several potentially interacting drivers, with the primary putative drivers including changes to herbivore management and fire regimes or increasing atmospheric carbon dioxide [7]. Fire is mostly limited to the western lowland ecoregions (East Sudanian savanna and Saharan flooded grasslands) and only up to 8% of the land area is burned annually [8], CO_2 enrichment is homogenous, while Ethiopia has the highest densities of domestic herbivores in Africa [9]. We therefore focused on the potential role of domestic herbivory in vegetation changes via overgrazing and densities of grazers versus browsers. Grazing can impact the cover and distribution of grasses, altering fire regimes and competitive interactions resulting in an increased cover in woody plants (Archer et al. 2017). This is often seen with high cattle densities, but not with high goat and sheep densities which, as browsers, consume more woody vegetation. Therefore, those cells that converted from grassland to savanna/shrubland, despite having high sheep and goat densities would most likely not be due to bush encroachment, whereas cells with high cattle densities could be a putative driver of shifts to woodier plant communities.. To decipher whether the main driver was bush encroachment (i.e. high cattle densities) or savanna recovery (high goat and/or sheep densities), we used a linear regression to identify the relationship between cells which changed from grassland to savanna or shrubland and the density of cattle, sheep and goats. If conversion of grassland to savanna or shrubland is due to bush encroachment, we would expect to see a positive relationship with cattle density and a no relationship or a negative relationship with sheep and goat density. Livestock densities (number of animals per 10km pixel) in 2015 were obtained from the Gridded Livestock of the World database v4 and resampled to our matching units [10].

Supplementary Methods 6 Sankey landcover changes

Land cover was derived from the MODIS Land Cover Type (MCD12Q1) Version 6 [11] dataset, resampled to 1km for the years 2001 and 2020. Sankey diagrams illustrate land cover changes from 2001 to 2020 for protected and unprotected gridcells, plotted via *networkD3* [12]. The percentage of forest, grass and agricultural land cover remaining the same was then compared inside and outside PAs.

Supplementary Methods 7 Socio-economic survey household attrition

Attrition rates between survey years were low and similar (14% and 12% respectively) between households close to protected areas (within 10km) and further away (more than 20km). While attrition can bias estimates, the low rates and small difference suggest only limited potential for this. Additionally, there is no significant difference in months of adequate food in 2011 between households that were lost over survey rounds and those that remained ($t_{595.6}$ = 1.61, p = 0.11).

Supplementary Methods 8 National Forest Priority Area counterfactual analysis

The statistical matching approach was repeated for National Forest Priority Areas (NFPAs). 1km gridcells within NFPA boundaries were classified as treatment units and gridcells outside of a 10km buffer zone around NFPAs and outside other protected areas were classified as control units. Treatment and control units were matched using the same covariates as the statistical matching for strict and less strict PAs (excluding baseline grassland and baseline agriculture, as the only outcome being assessed was forest change). Post-matching analysis confirmed covariate balance (standardised mean differences of less than 0.25) and robust sample sizes (100% of treatment units) had been achieved with nearest Mahalanobis matching with replacement. Average change in forest cover from 2000-2021 was the compared between matched treatment and control cells (Supplementary Figure S9).

Supplementary Methods 9 Justification for the use of matching methods

Protected areas are not randomly located and are often biased towards areas with lower opportunity costs, which have particular biophysical and socioeconomic characteristics which may also influence the outcomes of interest[13]. Simple comparisons between protected and unprotected areas are therefore inherently biased. Evaluating the causal impacts of protected areas, requires constructing a credible counterfactual (what would have happened if protection had not been put in place) which can be achieved through the use of quasi-experimental methods. Common approaches included statistical matching, difference-in-differences, synthetic control, instrumental variables and regression discontinuity and each of which relies on specific assumptions[14,15].

The suitability of different quasi-experimental approaches depends on the nature of the intervention being assessed, the available data and the spatial scale of inference [14,15]. In Ethiopia, protected areas have been established gradually over decades through context-specific decisions rather than a consistent eligibility threshold. There is no variable that plausibly influences protected areas designation but does not affect the outcomes of interest (land cover and human wellbeing changes) other than through protection. This rules out a valid instrumental variables[16] or regression discontinuity methods[17]. Synthetic control methods are best suited to discrete treatment event such as a

policy or a single protected area with a specific start date [14]. Difference-in-differences approaches require temporally consistent pre-and post-intervention data at comparable spatial resolution for both treated and untreated units [18], which were unavailable for our outcomes.

Among quasi-experimental methods, statistical matching combined with post-matching covariate-adjusted regression was the most appropriate design for our data and research questions. This approach is widely used in conservation impact evaluation [15,19], and provides a transparent and replicable way to reduce selection bias by constructing a comparable sample of treatment and control units that are statistically similar based on observable covariates. To ensure robust causal inference from matching, we followed established best practice guidelines[14,15]: all covariates likely to influence both protected area placement and the outcomes were included; balance was assessed post-matching and post-matching; covariate-adjusted regression was applied to reduce residual bias; and multiple matching algorithms were applied to test sensitivity to specification choice. While matching cannot account for potential unobserved confounders and is sensitive to the matching specification used, we explicitly assess these limitations. We conducted sensitivity analyses using *sensemakr* [20] to quantify the amount of variance in the treatment and outcome an unobserved confounder would need to explain compared to a benchmark observed covariate before it nullified the result.

Supplementary Methods 10 Socio-economic survey household offset information

The GPS locations of households surveyed in the Ethiopian Socioeconomic Survey are offset by 0-2km with 1% being offset by 10km. This ensures the known range for all points is 10km but limits the noise added by offsets. Additionally, offsets are constrained within Ethiopia's administrative zones.

Supplementary Methods 11 Estimating impacted populations

Using the average treatment effects for social wellbeing outcomes across the matched treatment (protected) and control households, we estimate the likely number of people impacted using the UN-adjusted gridded population count [1] at 1km resolution in 2011 (the start of the survey period used) summed across all locations within 10km of a protected area. To ensure a conservative estimate we first determined the highest population density at any of the surveyed household locations, and then removed any gridcells with a higher population than this before summing population counts. We also merged any overlapping buffers into a single polygon to avoid double counting. To convert this to the household level, we used the average number of people per household from the household survey which was 4.6.

Supplementary Methods 12 Stakeholder questionnaire

This questionnaire was sent to target respondents via email or taken as physical copies (in cases where internet access was problematic). Prior to being asked for consent, potential respondents were made aware of the purpose of the questionnaire, how their responses would be used, and assured it would be treated anonymously and confidentially. This process was approved through an ethics board (Ethics ID of 20251741251220900).

Questionnaire: Challenges, Opportunities, Methods, and Specific Information on Ethiopia's Protected Area Network
በኢትዮጵያ ጥብቅ ስፍራዎች ላይ የሚያጋጥሙ ፈተናዎች/ ተግዳሮቶች፣ እድሎች፣ ዘዴዎች እና ልዩ መረጃዎች

Thank you for participating in this questionnaire. Your valuable insights will contribute to understanding the challenges, opportunities, and methods to enhance the effectiveness of Ethiopia's protected area network. Please provide your feedback by answering the following questions. በዚህ ጥያቄ ላይ ስለተሳተፋችሁ እናመሰግናለን። ጠቃሚ አስተያየቶቻችሁን የኢትዮጵያ ጥብቅ ስፍራዎች ውጤታማነትን ለማጎልበት የሚያስችሉ ትንተናዎችን፣ እድሎችን እና ዘዴዎችን ለመረዳት አስተዋፅኦ ያበረክታሉ። እባኮ የሚከተሉትን ጥያቄዎች በመመለስ አስተያየታችሁን ይስጡ።

Section 1: **Demographic Information የዴሞግራፊ መረጃ**

1. How old are you? እድሜዎ ስንት ነው?

<21	
21-30	
31-40	
41-50	
51-60	
61-70	
>70	

2. What is your gender? ፆታዎ ምንድን ነው?

Male ወንድ	
Female ሴት	

3. What is your highest level of education? የእርስዎ ከፍተኛ የትምህርት ደረጃ ምንድን ነው?

Primary school የመጀመሪያ ደረጃ ትምህርት	
Secondary school ሁለተኛ ደረጃ ትምህርት	
Bachelor's degree የባችለር ዲግሪ	
Masters degree ማስተርስ ዲግሪ	
PhD ዶክትሬት	
Other (please specify) ሌላ (እባክዎን ያመልክቱ): ___________________________________	

4. What type of organisation do you work for? በምን አይነት መስሪያ ቤት ውስጥ ነው የሚሰሩት?

University ዩኒቨርሲቲ	
Research institute የምርምር ተቋም	
NGO መንግስታዊ ያልሆነ ድርጅት	
Private/consultant የግል/አማካሪ	
National government ብሔራዊ መንግሥት	
Regional government ክልላዊ መንግስት	
Other (please specify) ሌላ (እባክዎን ያመልክቱ):_____________________________________	

Section 2: **Future of Ethiopia's protected areas የኢትዮጵያ ጠባቂ አካባቢዎች ወደፊት**

1. What should be the goals of Ethiopia's protected areas over the next 20 years? በሚቀጥሉት 20 ዓመታት የኢትዮጵያ ጥብቅ ስፍራዎች ግባቸው ምን መሆን አለበት?

Please rank 1-3 with 1 being the highest priority and 3 being the lowest እባክዎ ደረጃ 1-3 ይስጡ 1 ከፍተኛ ቅድሚያ እና 3 ዝቅተኛ መሆን

Expanding the protected area network<br>ጥብቅ ስፍራዎችን ማስፋት	
Making the existing protected area network more effective<br>አሁን ያሉትን ጥብቅ ስፍራዎች ውጤታማ ማድረግ	
Carrying out additional research to understand how to improve the protected area network<br>ጥብቅ ስፍራዎችን እንዴት ማሻሻል እንደሚቻል ለመረዳት ተጨማሪ ምርምር ማካሄድ	

Section 3: **Effectiveness of Ethiopia's protected area network የኢትዮጵያ ጥብቅ ስፍራውች ይዘት ውጤታማነት**

2. Do you feel that Ethiopia's protected areas are effective at የኢትዮጵያ ጥበቃ ቦታዎች የተደረገላቸው አካባቢዎች ከስር በተዘረዘሩት መስኮች ውጤታማ እንደሆኑ ይሰማችኋል:

	Yes (አዎ)	No (አይ)	I don't know (አላውቅም)
Reducing forest loss<br>የደን መጥፋት መቀነስ			
Preventing agricultural expansion with protected area boundaries<br>በጥብቅ ስፍራ ድንበሮች አካባቢ የእርሻ መስፋፋትን መከላከል			
Conserving grassland<br>የሳር መሬት ጥበቃ			
Reducing poverty among neighbouring communities<br>በአጎራባች ማህበረሰቦች መካከል ያለውን ድህነት መቀነስ			
Increasing food security among neighbouring communities<br>በአጎራባች ማህበረሰቦች መካከል የምግብ ዋስትና እየጨመረ መጥቷል			

Section 4: **Challenges ተፈታታኝ ሁኔታዎች/ ተግዳሮቶች**

3. What are the three biggest challenges which threaten the effectiveness of Ethiopia's protected area network? የኢትዮጵያ ጥብቅ ስፍራዎችን ውጤታማነት ስጋት ላይ የሚጥሉት ሶስት ትልልቅ ፈተናዎች ምንድን ናቸው?

Select only three most important *በጣም አስፈላጊ የሆኑትን ሶስት ብቻ ይምረጡ እና*

Inadequate community engagement<br>በቂ ያልሆነ የማህበረሰብ ተሳትፎ	
Limited public awareness<br>የህዝብ ግንዛቤ ውስንነት	
Land use conflict<br>የመሬት አጠቃቀም ግጭት	
Lack of collaboration between stakeholders<br>በባለድርሻ አካላት መካከል ያለው ትብብር አለመኖር	
Weak law enforcement<br>የህግ ማስክብር ድክመትደካማ ሕግ አስከባሪ	
Inadequate financial resources<br>በቂ የገንዘብ ሀብት አለማግኘት	
Climate change የአየር ንብረት ለውጥ	
Inadequate representation of threatened species<br>አደጋ ላይ የወደቁ ዝርያዎች በበቂ ሁኔታ አለመወከል	
Alien and Invasive species<br>መጤ እና ወራሪ ዝርያዎች	

Expansion of agriculture<br>የእርሻ መስፋፋት	
Settlement and Urbanisation<br>የሰፈራና ከተማ መስፋፋት	
Political conflict<br>የፖለቲካ ግጭት	
Lack of food security among local communities<br>በአካባቢው ማህበረሰብ ዘንድ የምግብ ዋስትና እጥረት	
Free Grazing within protected area boundaries<br>በጥብቅ ቦታዎች ውስጥ ልቅ ግጦሽ	
Poverty in neighbouring areas<br>በአጎራባች አካባቢዎች ድህነት	
Other<br>ሌላ	

4. If other please specify ሌላ ካለ እባክዎን ይግለጹ:

____

Section 5: **Priorities ቅድሚያ የሚሰጣቸው**

5. What are the three most important actions which should be prioritised to improve the effectiveness of Ethiopia's protected area network? የኢትዮጵያን ጥብቅ አካባቢዎች ውጤታማነት ለማሻሻል ቅድሚያ ሊሰጣቸው የሚገቡ ሶስት አስፈላጊ እርምጃዎች ምንድን ናቸው?

Select only three most important. *በጣም አስፈላጊ የሆኑትን ሶስት ብቻ ይምረጡ እና*

Strengthening community engagement<br>የህብረተሰቡን ተሳትፎ ማጠናከር	
Improving public awareness<br>የህዝብን ግንዛቤ ማሻሻል	
Enhancing partnerships/collaborations with local communities, NGOs, and academic institutions<br>የአካባቢው ማህበረሰብ ከመንግስታዊ ካልሆኑ ድርጅቶችና ከትምህርት ተቋማት ጋር አጋርነትን/ትብብርን ማጎልበት	
Developing sustainable nature-based tourism<br>ተፈጥሮ-ላይ የተመሠረተ ዘላቂ ቱሪዝም ማዳበር	
Strengthening policy and enforcing the law around protected area management<br>ጥበቃ በተደረገለት አካባቢ አስተዳደር ዙሪያ ፖሊሲንና የህግ ትግበራን ማጠናከር	
Expanding and establishing new protected areas<br>አዳዲስ ጥብቅ ቦታዎችን ማስፋትእና ማቋቋም	
Incorporating traditional ecological knowledge and practices into protected area management<br>ባህላዊ እና ሥነ-ምህዳራዊ ዕውቀቶችንን እና ተግባራትን ወደ አካባቢ ጥበቃ አስተዳደር ማምጣት	
Increasing funding resources<br>የገንዘብ ድጋፍ እየጨመረ መሄድ	
Promoting research to inform evidence-based conservation strategies<br>በማስረጃ ላይ የተመሰረቱ የጥበቃ ስልቶችን ለማሳወቅ ምርምርን ማስፋፋት	
Improving food security<br>የምግብ ዋስትና ማሻሻል	
Reducing poverty<br>ድህነትን መቀነስ	
Reducing conflict	

ግጭትን መቀነስ	
Strengthening capacity building and training for park rangers and staff<br>የአቅም ግንባታ እና ስልጠና ለአካባቢ ጥበቃ ሰራተኞች መስጠትን ማጠናከር	
Implementing robust monitoring and evaluation systems<br>ጠንካራ ክትትል እና ግምገማ ስርዓት መተግበር	
Other ሌላ	

6. If other please specify ሌላ ካለ እባክዎን ይግለጹ:

____

Supplementary results

Supplementary Results 1 Newer protected areas are being established in areas of higher human pressure

Spearman's rank correlations indicate that more recently established protected areas tend to be located in areas with higher population density ($r_s = 0.24$, $p < 0.001$), and more agricultural land ($r_s = 0.24$, $p < 0.001$). They are also weakly associated with shorter travel times to cities ($r_s = –0.045$, $p < 0.001$) and higher elevations ($r_s = 0.096$, $p < 0.001$) indicating they newer protected areas are generally being established in areas of higher human pressure.

Supplementary Results 2 Human and land-use pressures across under- and over- represented ecoregions

Blocked permutation tests show that underrepresented ecoregions had higher agricultural land cover (Underrepresented = 19.68, Overrepresented = 9.76; p = 0.002, FDR-adjusted p = 0.005), shorter travel time to cities (Underrepresented = 538.14, Overrepresented = 638.92; p = 0.002, FDR-adjusted p = 0.005), and higher population density (Underrepresented = 64.37, Overrepresented = 47.14; p = 0.030, FDR-adjusted p = 0.050). We found no evidence of differences for agricultural suitability (FDR-adjusted p = 0.47) or elevation (FDR-adjusted p = 0.47).

Supplementary Results 3 Overall land cover changes

Analysis of land cover changes across the whole landscape from 2001-2020 (Supplementary figure S6) showed that natural land cover classes were more likely to remain stable inside protected areas. For example, a larger proportion of forest remained as forest inside protected areas (88%) compared to outside (76%). Grassland also remained more stable inside protected areas (92% compared to 84% outside). By comparison, a larger proportion of cropland reverted to natural vegetation inside protected areas (64%) compared to outside (20%). Across all surveyed households, months of adequate food declined by half a month in households within 10km of a protected area while remaining stable in households more than 20km from a protected area; and material wellbeing remained stable for households close to protected areas while improving further away. Conversely dietary diversity increased 15% more among households close to protected areas.

Supplementary Results 4 Interpreting grassland changes

The most dominant landcover changes either to or from grassland were with cropland, savanna and shrubland (Supplementary Figure S10). In the context of biodiversity conservation, changes from cropland to grassland is interpreted as positive impact, while changes from grassland to cropland is negative. Changes from grassland to savanna or shrubland could either be a) savanna recovery from grassland (where loss of grassland would be a positive impact in PAs) or bush encroachment as a result of grassland degradation (where loss of grassland would be a negative impact in PAs). We found significant positive correlations between matching units which changed from grassland to either savanna or shrubland and the density of cattle, and negative relationships with density of sheep and goat (Supplementary Table 16). This suggests that loss of grassland due to bush encroachment is more likely than savanna recovery as sheep and goat are expected to reduce woody plant cover, while cattle are expected to

reduce grass cover, while increasing woody plant cover. Bush encroachment transitions are negative from a conservation perspective and therefore loss of grassland in this case is viewed as a negative. In strict PAs, we saw greater increase in grassland in PAs compared to matched control (main text Fig 3) and sankey diagrams demonstrate that the majority of increases in grassland were from cropland (Supplementary Figure S10A) which suggests abandonment of farms may be the main driver and this is a positive impact. In less strict PAs we saw less decline in grassland compared to matched controls (main text Fig. 3), and the majority of this was to savanna or shrubland (Supplementary Figure S10C), through bush encroachment. This may be due to livestock foraging occurring more frequently in less strict compared to strict protected areas (although at lower intensities than outside), suggesting that although woody cover is still increasing in less strict protected areas, management is having a positive impact at reducing the extent and/or rate of change.

Supplementary text

Supplementary Text 1 Context for win-win sites

We identify five protected areas experiencing win-win outcomes: Yayu Biosphere Reserve, Gibe Sheleko National Park, Erer-Gota Controlled Hunting Area, Borena National Park, and Kafta Sheraro National Park. Below we summarise plausible descriptive reasons consistent with these results.

Yayu Biosphere Reserve

Households in Yayu obtain substantial income for forest coffee and other non-timber forest products [21]. This aligns both livelihood and conservation incentives for maintaining forest. Additionally it has a high budget (Supplementary Table 10) and associations with the international climate initiative with a project focused on restoring degraded coffee landscapes in Ethiopia [22]

Gibe Sheleko National Park

Gibe Sheleko is located in an area of high agricultural suitability[23] which may help communities to maintain food security with less expansion of agricultural land. Local livelihoods include honey bee rearing and eucalyptus plantations, and non-timber forest products and are recognised by local communities as a benefit from the park, improving their perceptions of it [24].

Erer-Gota Controlled Hunting Area

Ethiopia’s controlled hunting area framework allows community revenue shares, and contributes to the rural economies neighbouring the parks[25] It is located in a hot arid region of Ethiopia that more suited to pastoralist lifestyles reducing pressure of agricultural conversion[26].

Borena National Park

Borena is also located in an arid region dominated by pastoralist livelihoods[26]. In Borana zone, Gadaa based institutions historically organised pasture use and participatory rangeland management is prioritised[27]. It also has a comparatively high budget (Supplementary Table 10).

Kafta Sheraro National Park

Kafta Sheraro also has a high budget (Supplementary Table 10) which likely supports better environmental outcomes and it is has a flagship transboundary elephant population making it a national priority and attracting external support[28].

Supplementary figures

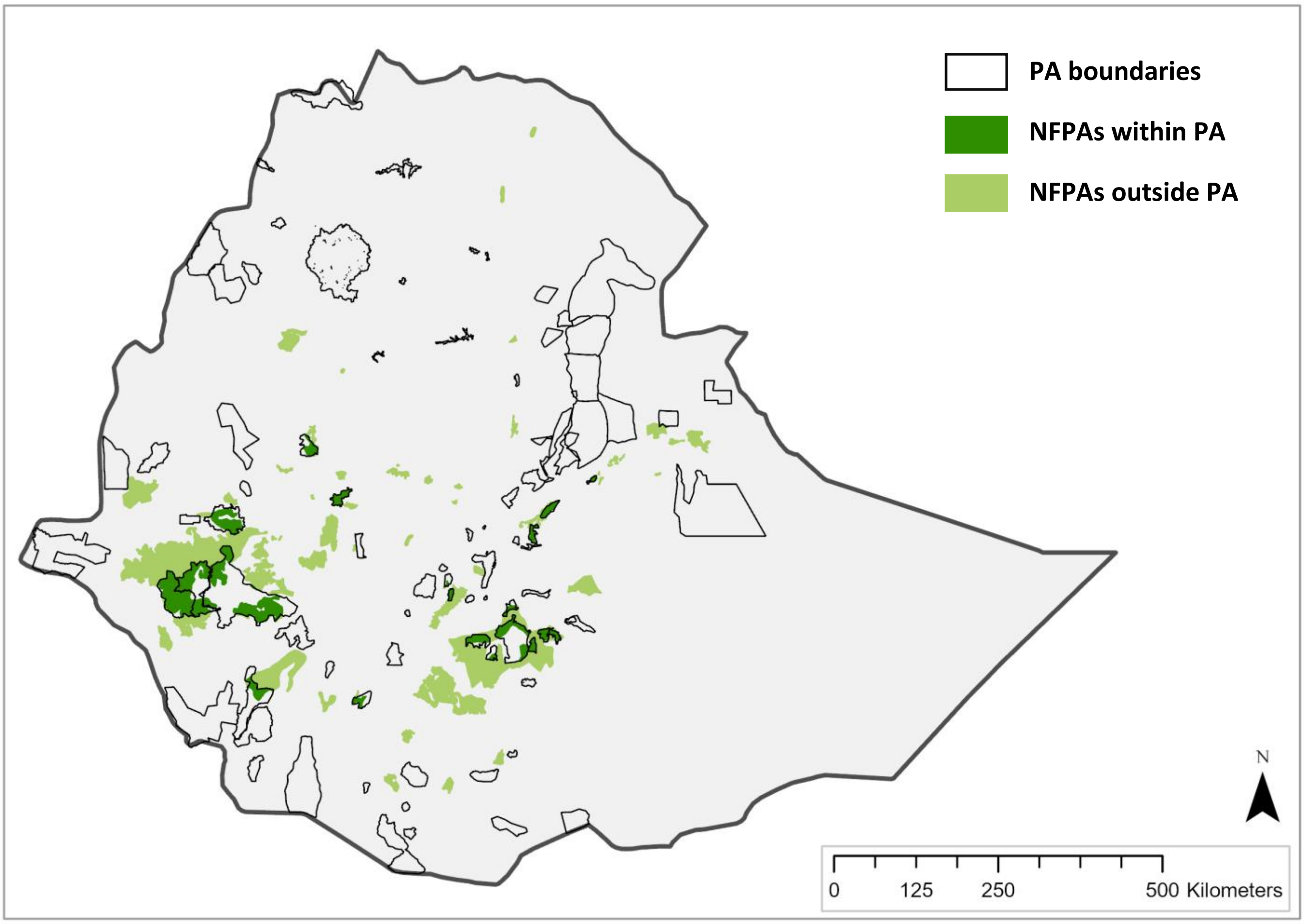

Supplementary Figure 1 The overlap of National Forest Priority Areas (NFPAs) with gazetted protected areas

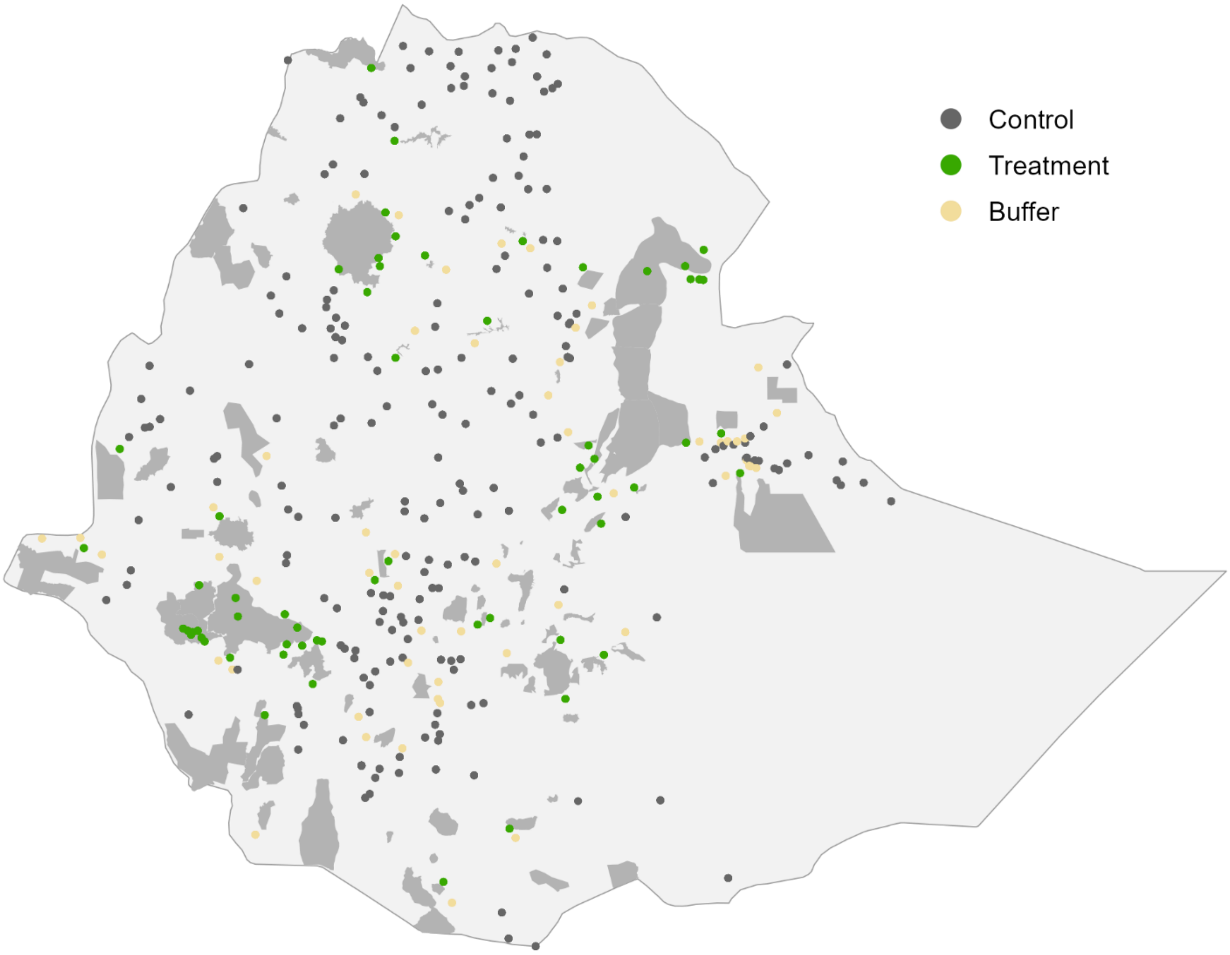

Supplementary Figure 2 Map of enumeration area locations from households surveyed in both the 2011 and 2016 Living Standards Measurement Survey[29,30] (333 enumeration areas comprising 3917 households). The control pool (2560 households) are more than 20km from a protected area, treatment pools (721 households) are less than 10km from a protected area and buffer pools (636 households) are in between.

A

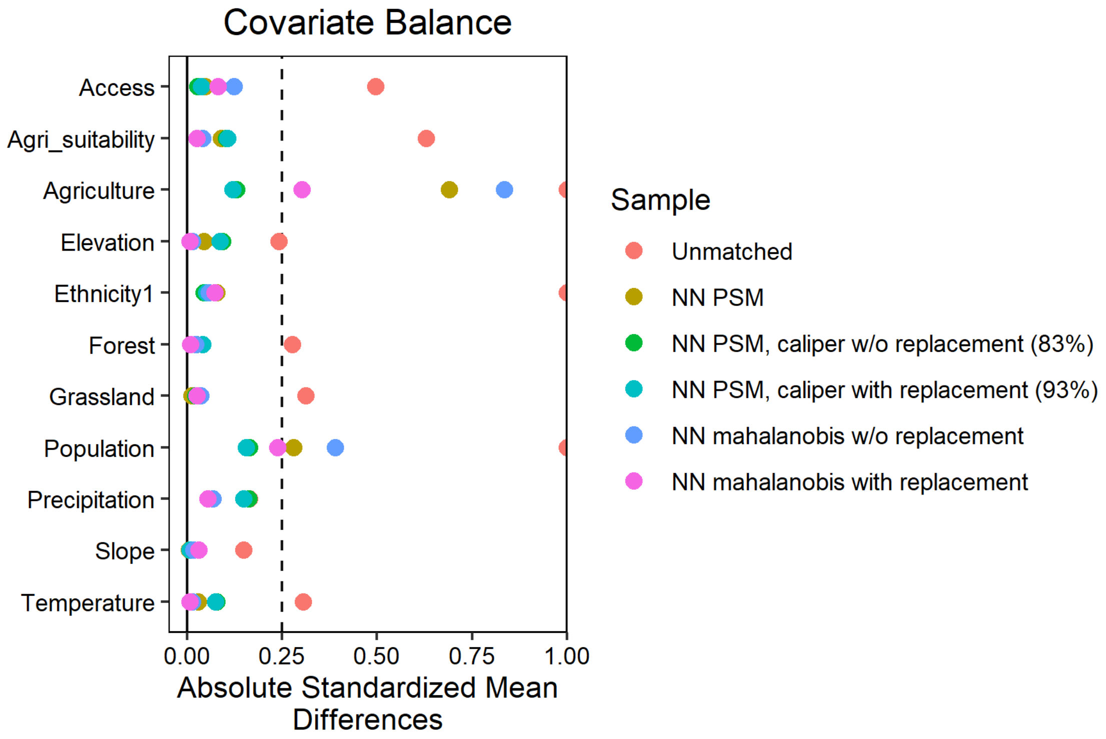
Covariate Balance
Access
Agri_suitability
Agriculture
Elevation
Ethnicity1
Forest
Grassland
Population
Precipitation
Slope
Temperature
0.00
0.25
0.50
0.75
1.00
Absolute Standardized Mean Differences
Sample
Unmatched
NN PSM
NN PSM, caliper w/o replacement (83%)
NN PSM, caliper with replacement (93%)
NN mahalanobis w/o replacement
NN mahalanobis with replacement

B

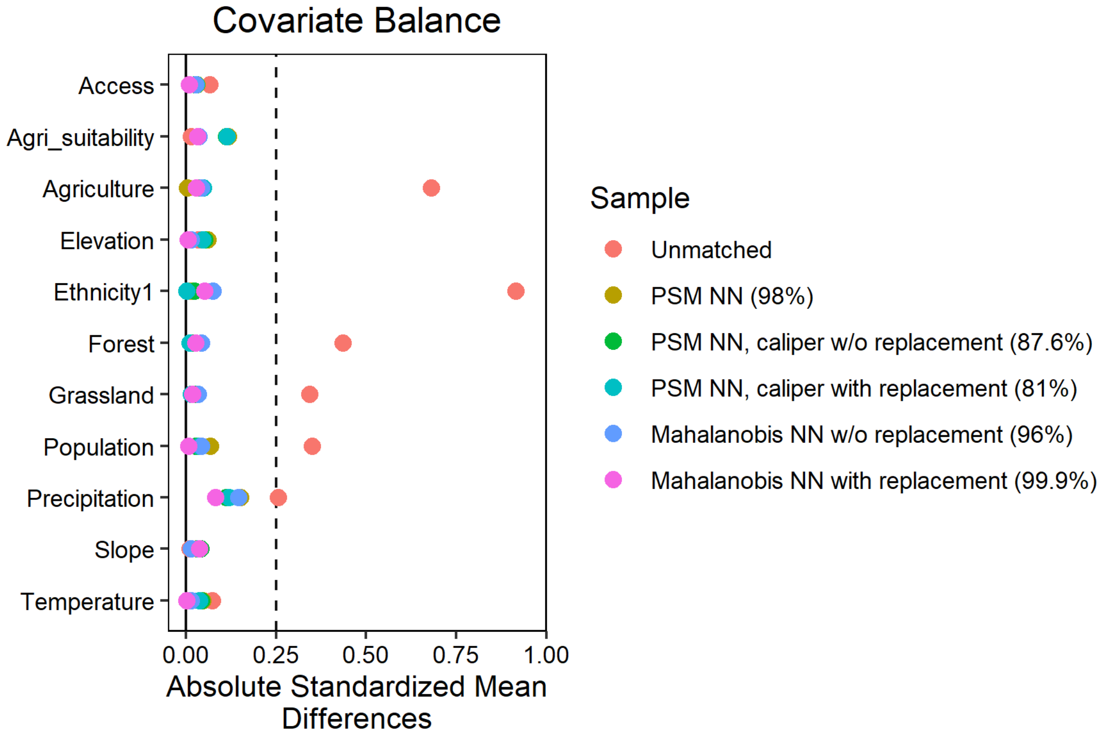
Covariate Balance
Access
Agri_suitability
Agriculture
Elevation
Ethnicity1
Forest
Grassland
Population
Precipitation
Slope
Temperature
0.00
0.25
0.50
0.75
1.00
Absolute Standardized Mean Differences
Sample
Unmatched
PSM NN (98%)
PSM NN, caliper w/o replacement (87.6%)
PSM NN, caliper with replacement (81%)
Mahalanobis NN w/o replacement (96%)
Mahalanobis NN with replacement (99.9%)

C

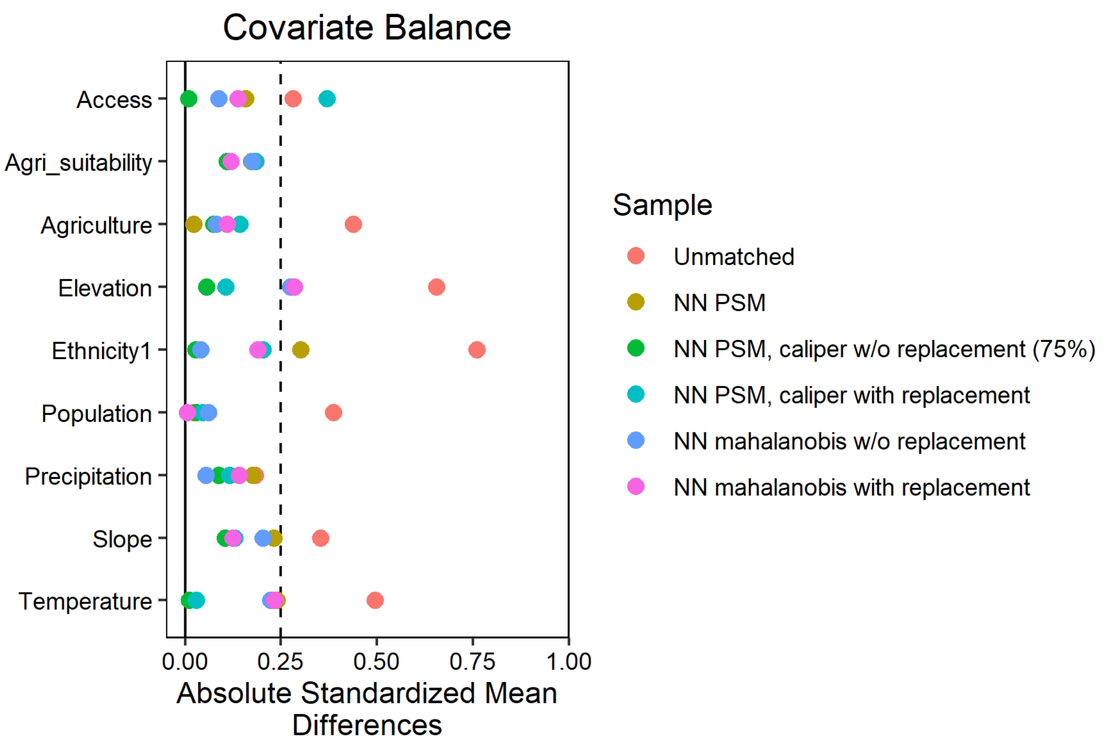

Supplementary Figure 3 Comparison of statistical matching specifications. Comparing the covariate balance achieved and sample unit retention (shown in in the legend) across six statistical matching specifications for (A) statistical matching for strict protected areas, (B) statistical matching for less strict, and (C) household matching. Different coloured points represent different matching specification. The matching specifications selected for further analysis were the specifications with the highest sample unit retention which achieved covariate balance below the threshold of 0.25 (dashed line). PSM = propensity score matching, NN = nearest neighbour.

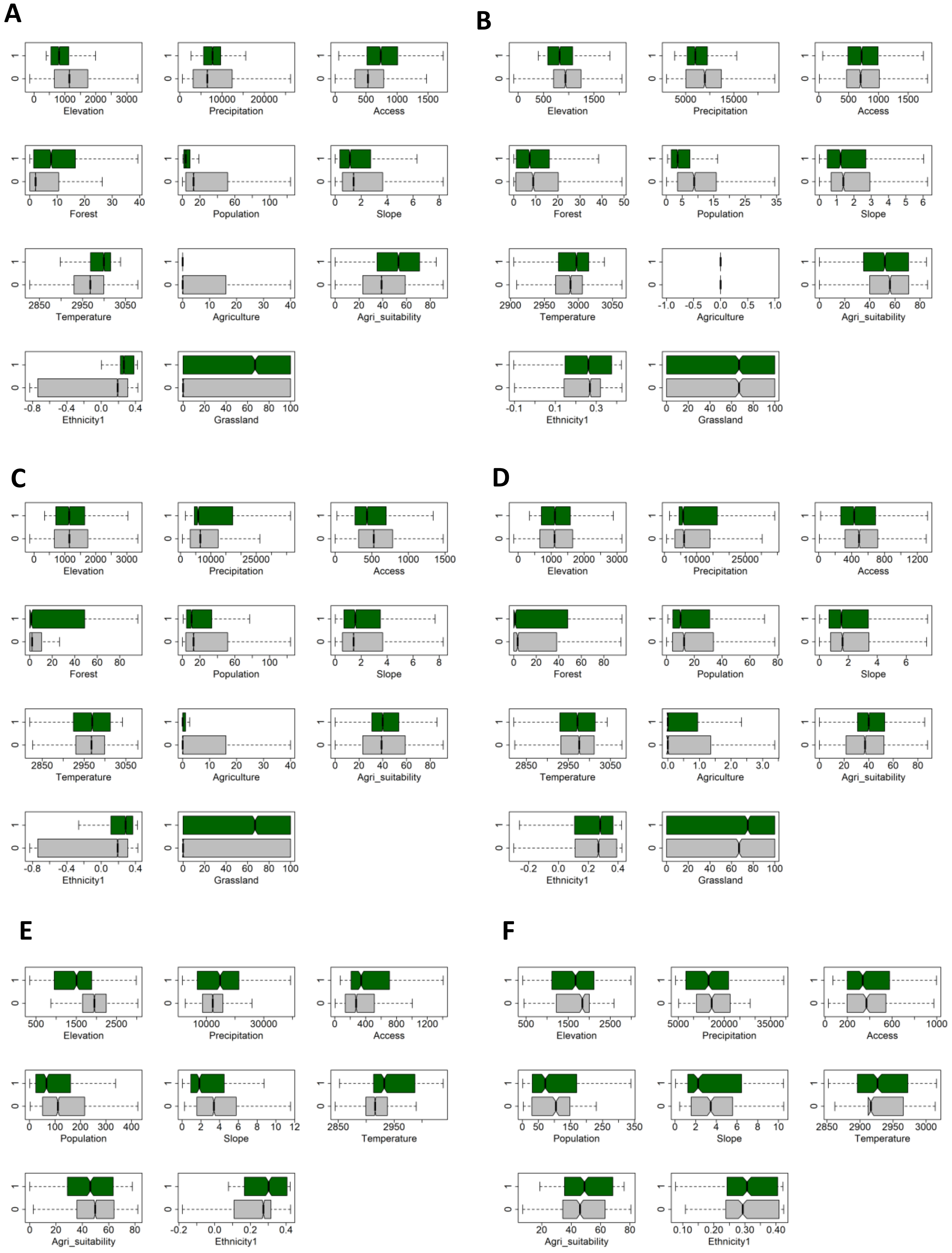

Supplementary Figure 4 Comparison of treatment (green boxes) and control (grey boxes) covariate values for (A) strict pre-match data, (B) strict post-match data, (C) less strict pre-match data, (D) less strict post-match data, (E) household pre-match data and (F) household post-match data.

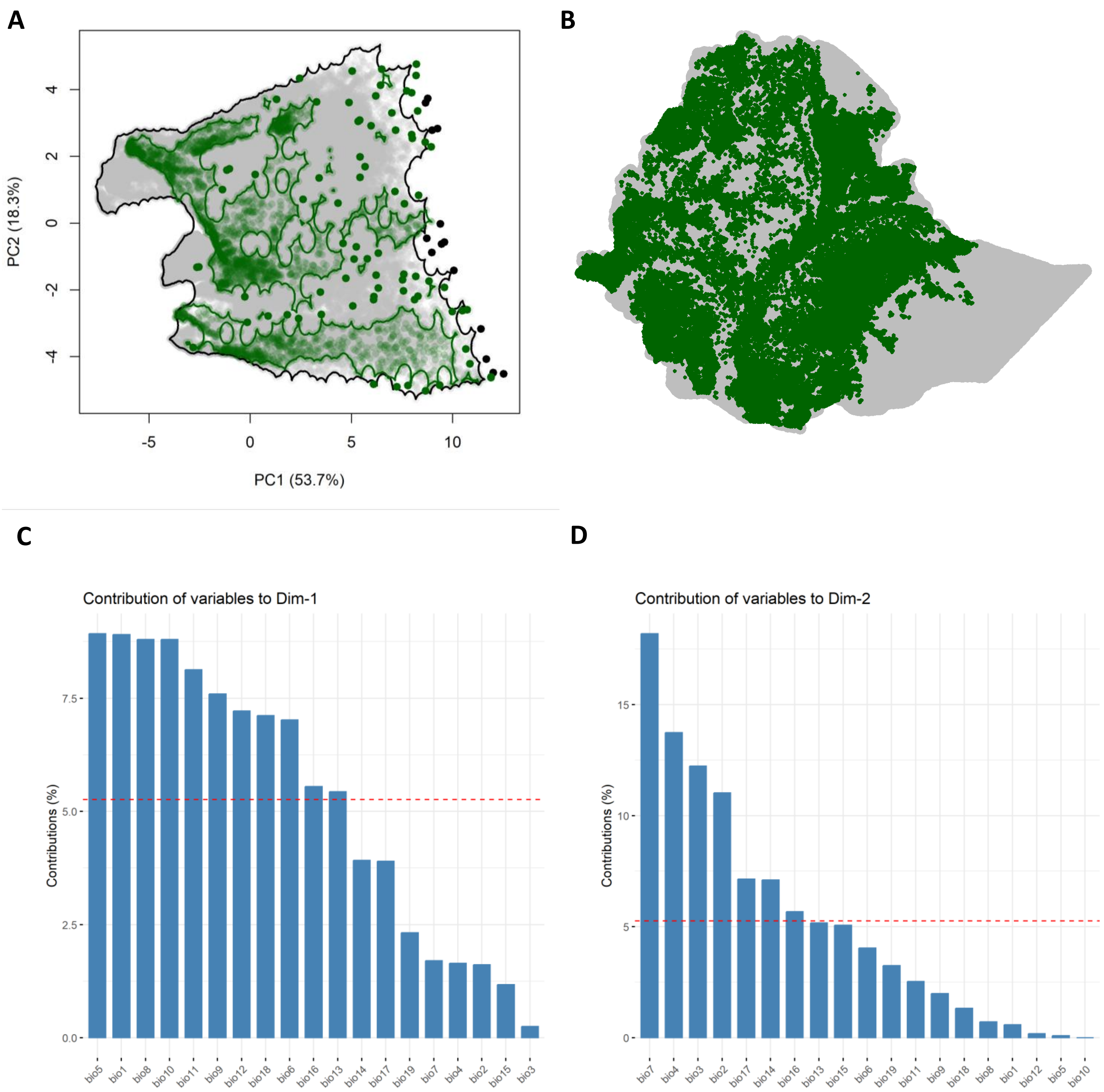

Supplementary Figure 5 Representativeness of Ethiopia's environmental space within the protected area network. (A) shows a principal component analysis plotting Ethiopia's background environmental space (grey) and the environmental conditions found within the protected area network (green), with alpha hulls where alpha is set to 0.3. 33% of the Ethiopia's environmental space matched the environment found within protected areas. (B) shows the PCA converted into geographical space, where green highlights areas which match the environmental conditions already found within the protected area network. Variable loadings for the first and second principal component axes are shown in (C) and (D) respectively, where dashed lines represent the value expected if all contributions were uniform.

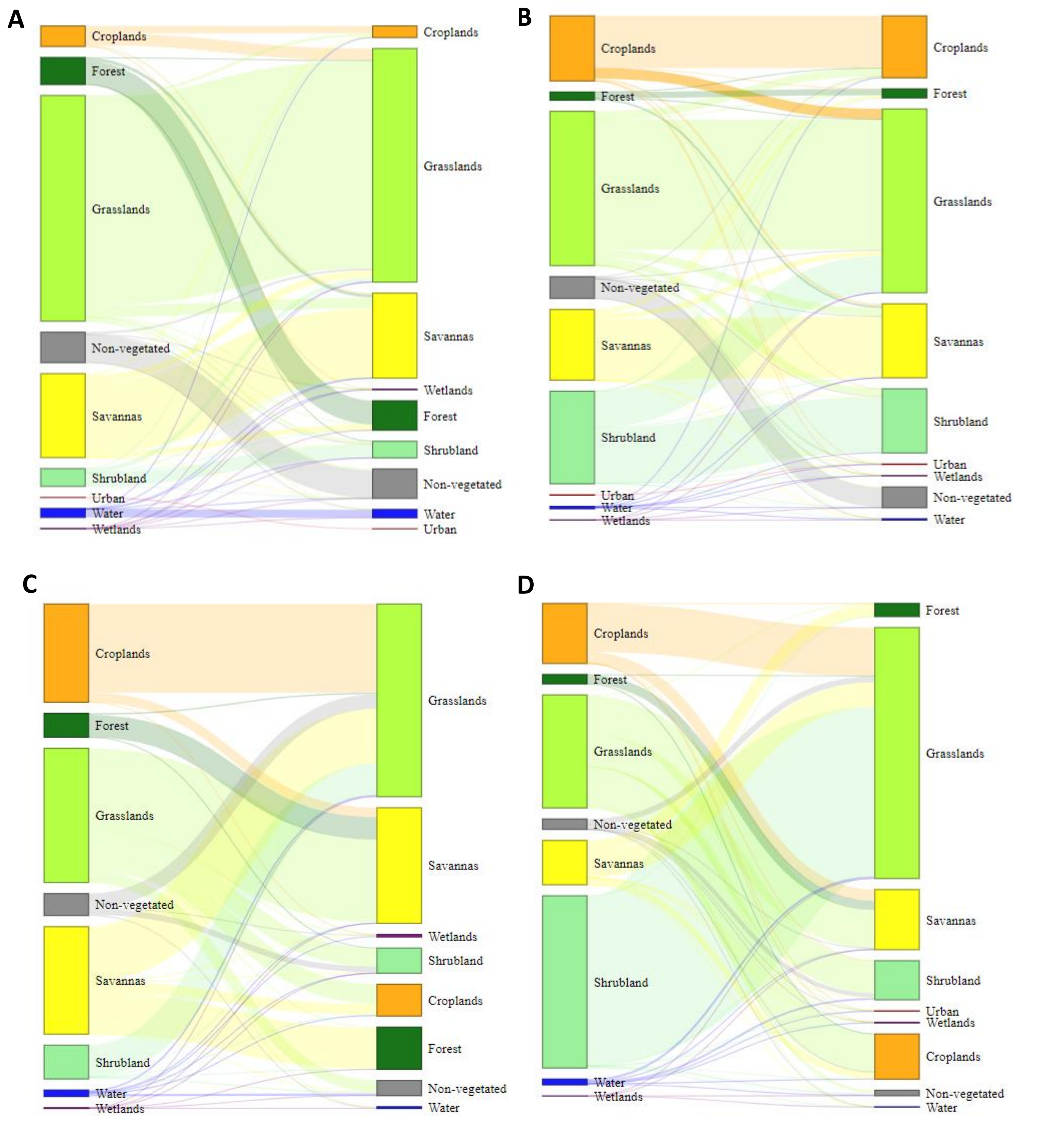

Supplementary Figure 6 Landcover changes in each 1x1km gridcell that occurred between 2000 and 2021 (A) inside PAs, (B) outside PAs, (C) inside PAs excluding gridcells where the landcover remained constant and (D) outside PAs excluding gridcells where the landcover remained constant.

(**A**) Matching treatment: **Strict PAs**; outcome variable: **Forest** (207 models with sufficient balance and sample size)

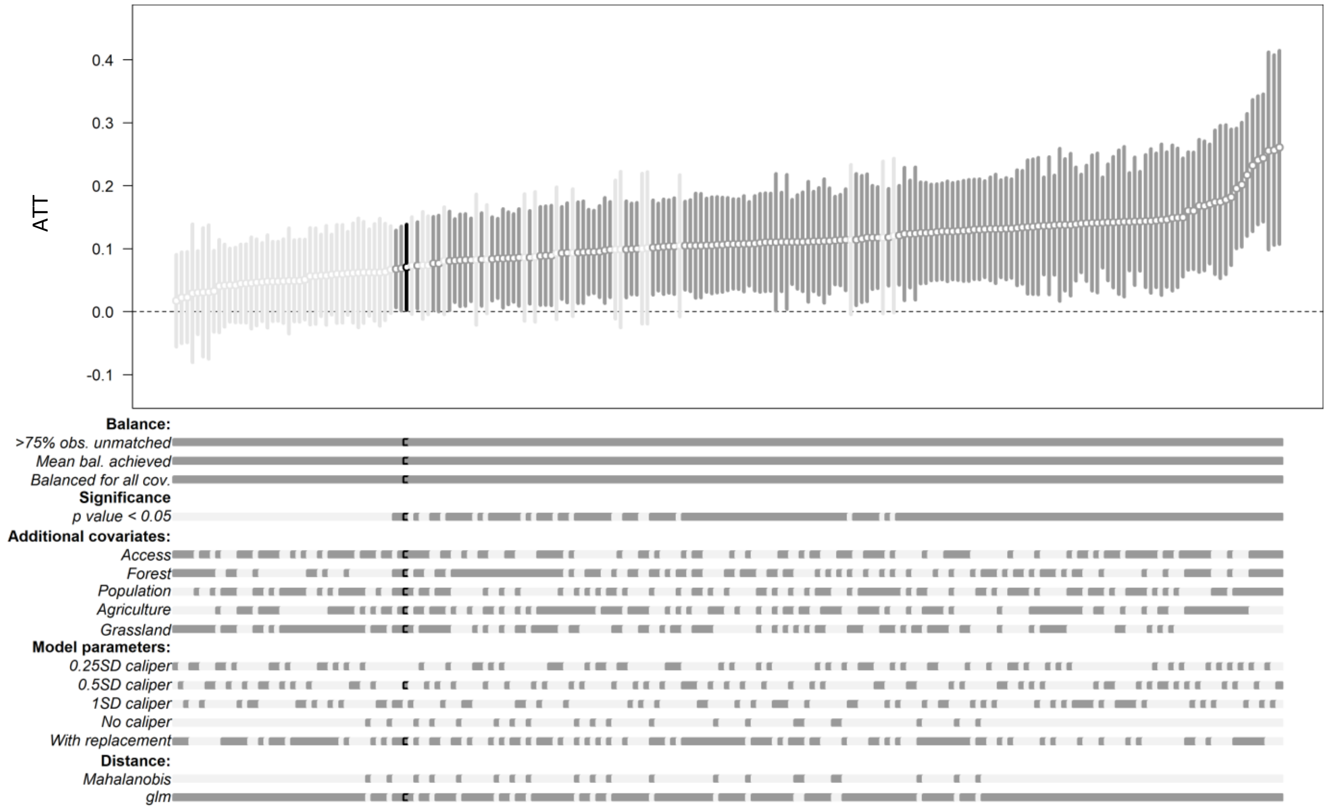

(**B**) Matching treatment: **Less strict PAs**; outcome variable: **Forest**; (248 models with sufficient balance and sample size)

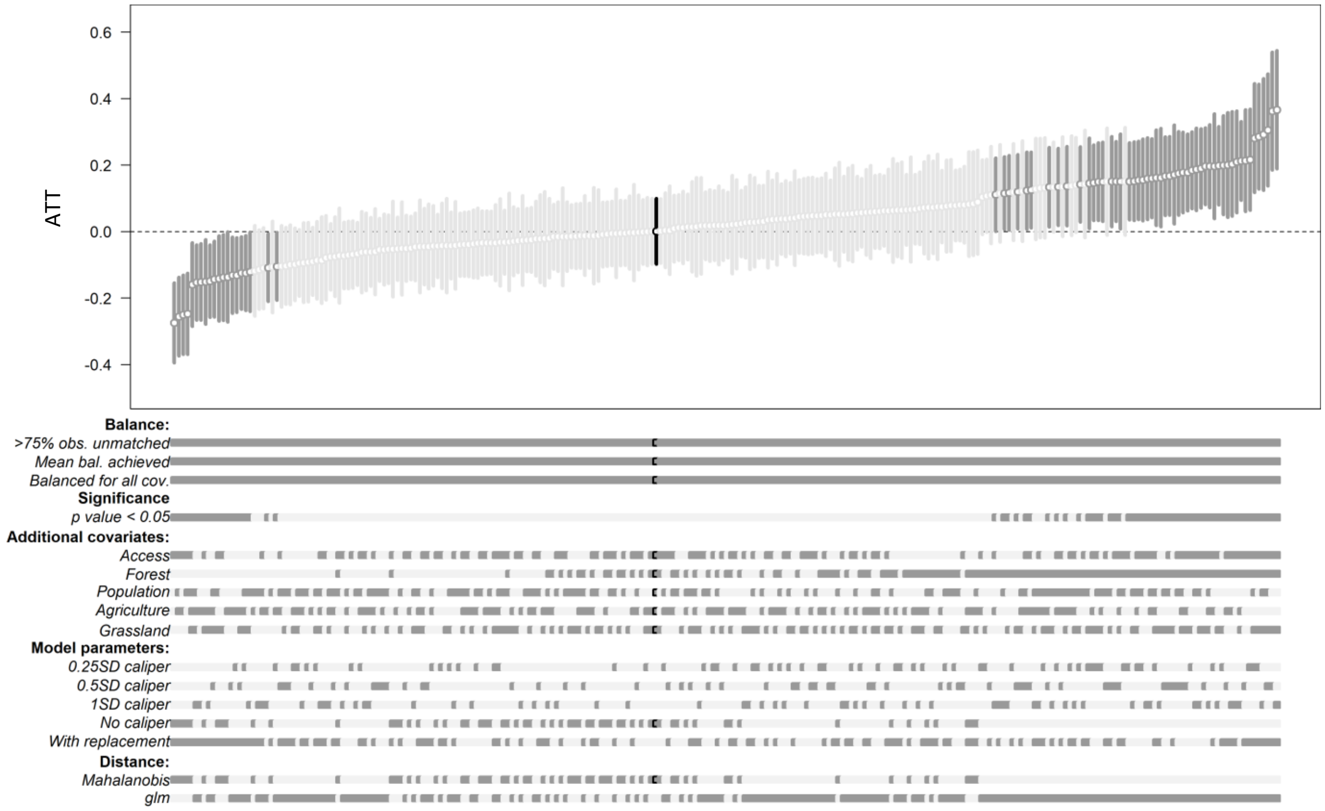

(**C**) Matching treatment: **Strict PAs**; outcome variable: **Grassland** (207 models with sufficient balance and sample size)

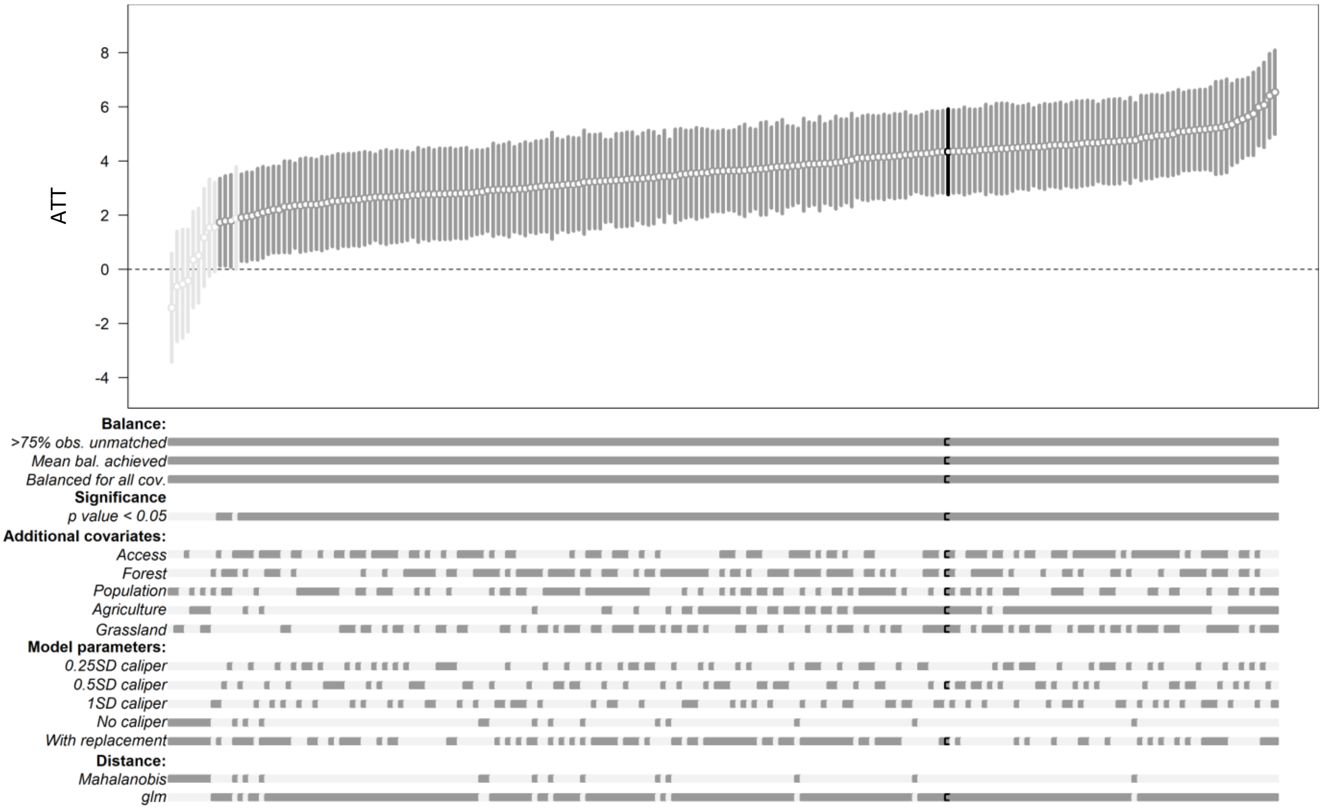

(**D**) Matching treatment: **Less strict PAs**; outcome variable: **Grassland** (248 models with sufficient balance and sample size)

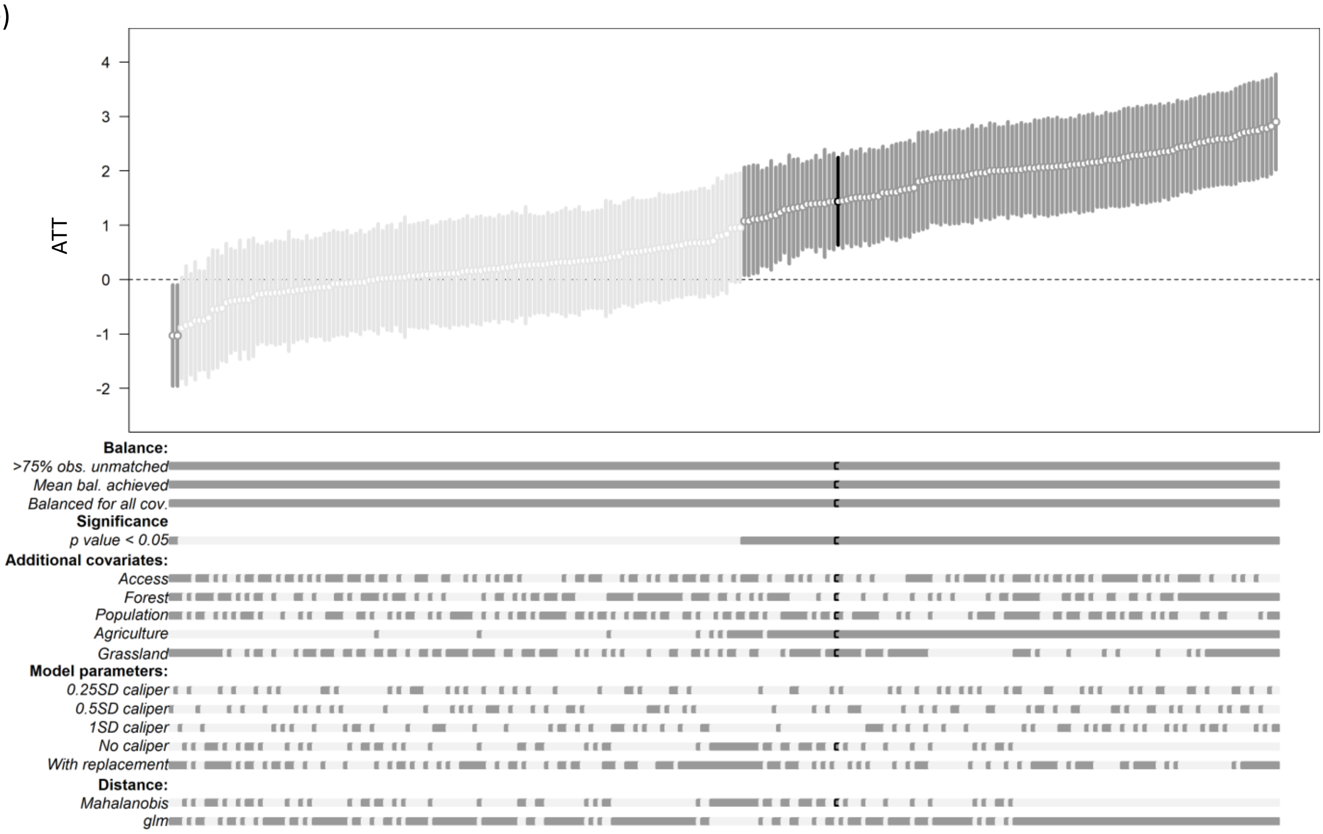

(**E**) Matching treatment: **Strict PAs**; outcome variable: **Agriculture** (207 models with sufficient balance and sample size)

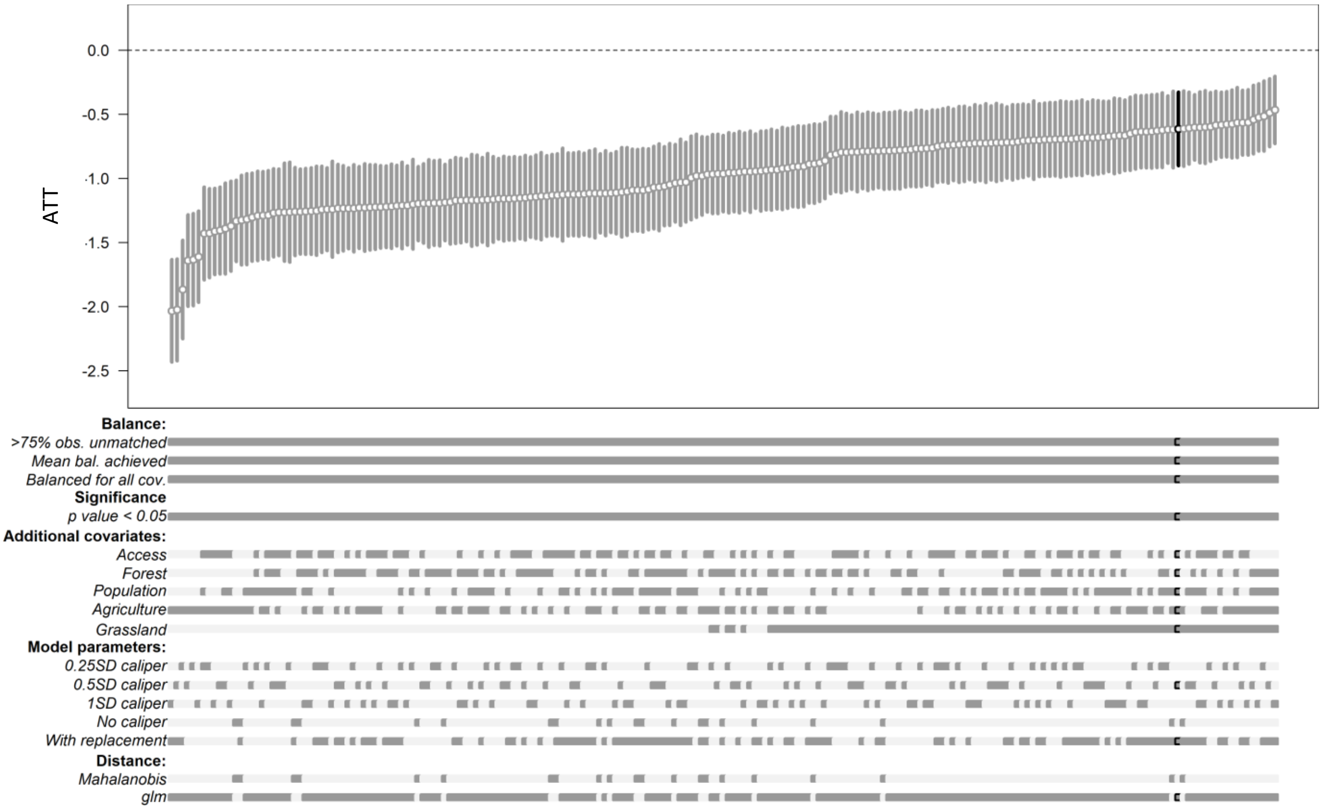

(**F**) Matching treatment: **Less strict PAs**; outcome variable: **Agriculture** (248 models with sufficient balance and sample size)

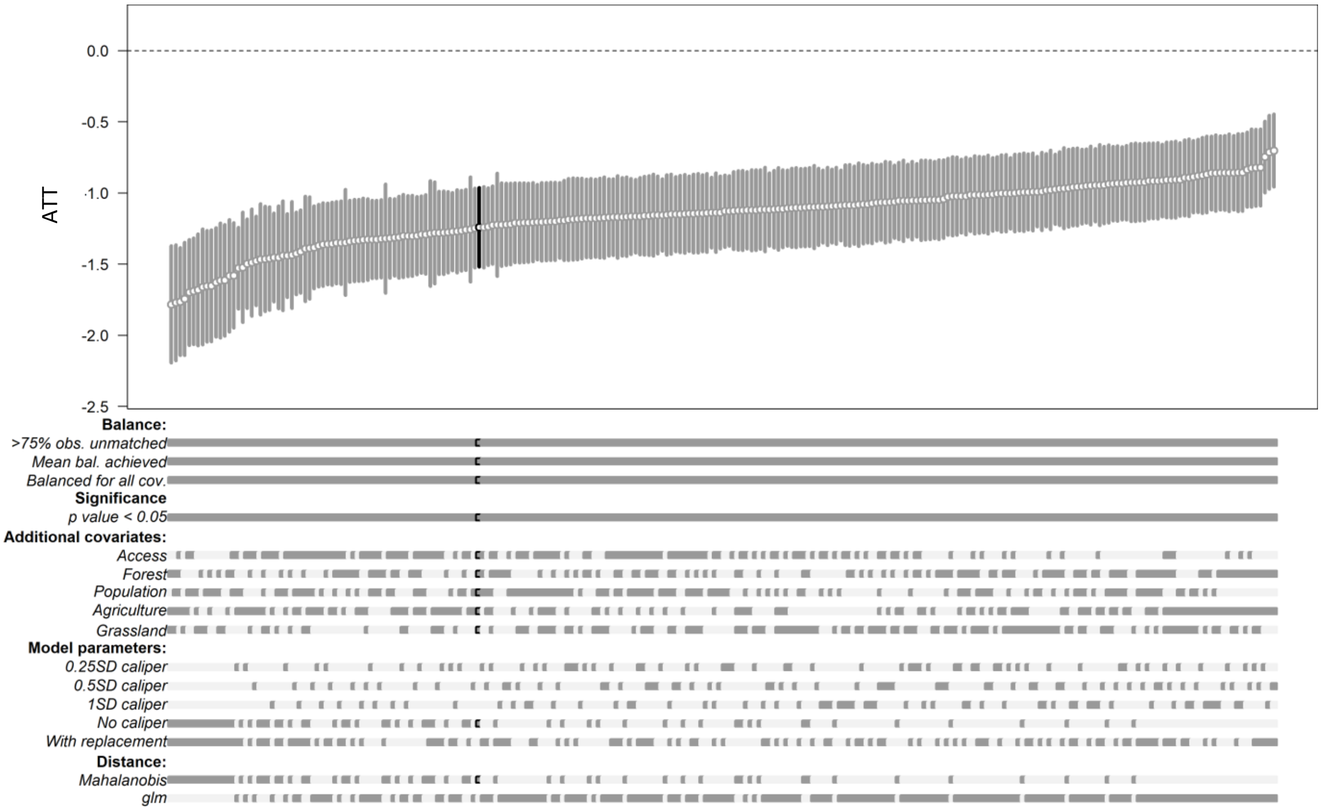

(**G**) Matching treatment: **Households near PAs**; outcome variable: **Months of adequate food** (17 models with sufficient balance and sample size)

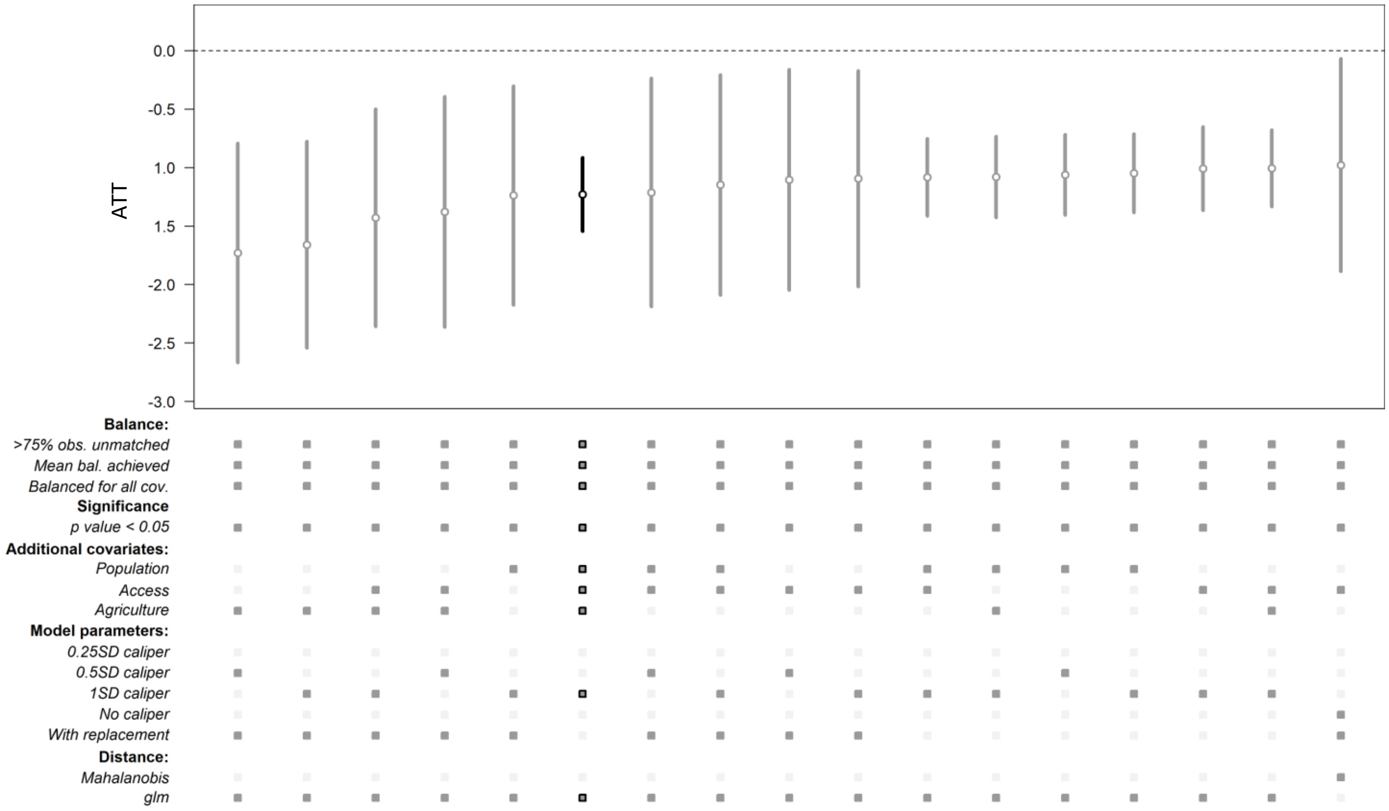

(**H**) Matching treatment: **Households near PAs**; outcome variable: **Dietary diversity** (17 models with sufficient balance and sample size)

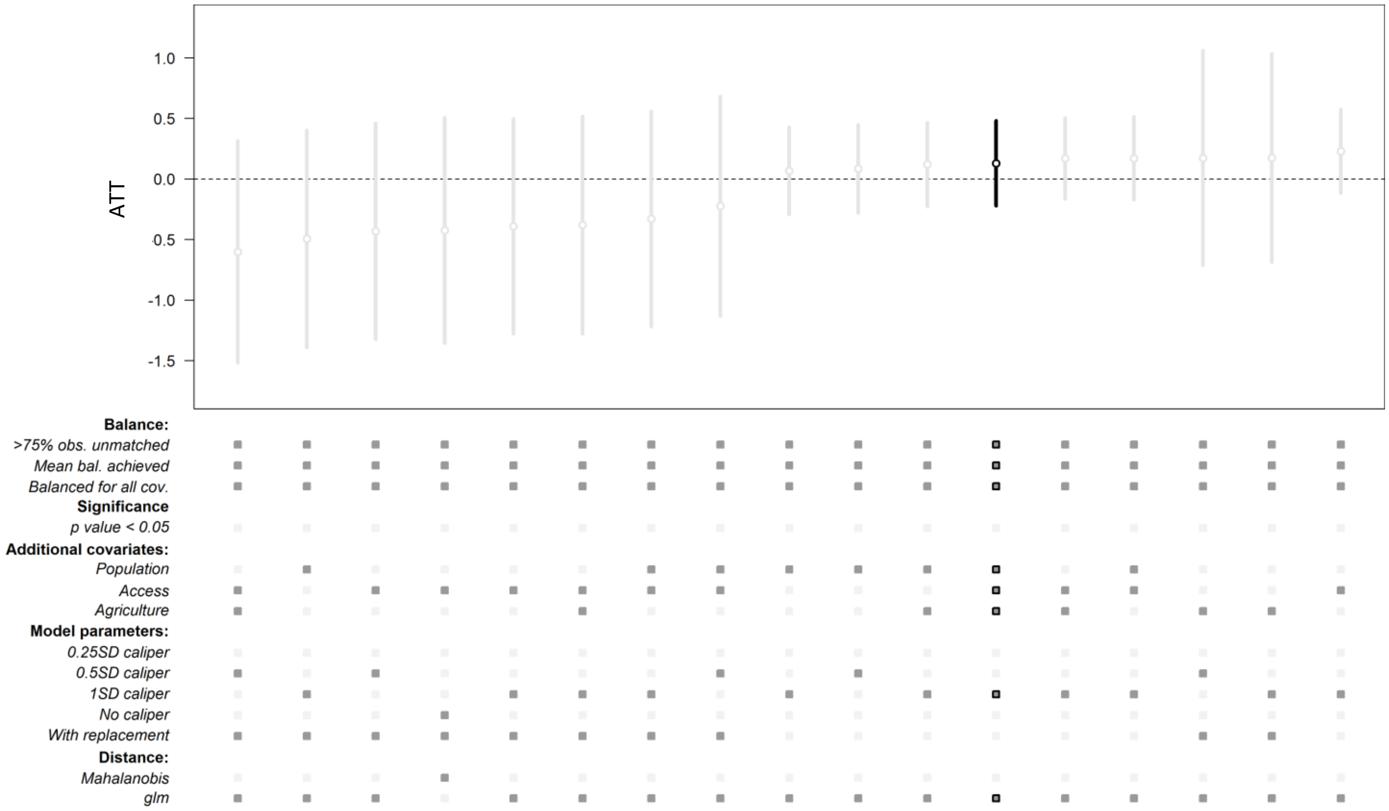

(**I**) Matching treatment: **Households near PAs**; outcome variable: **Material wellbeing** (17 models with sufficient balance and sample size)

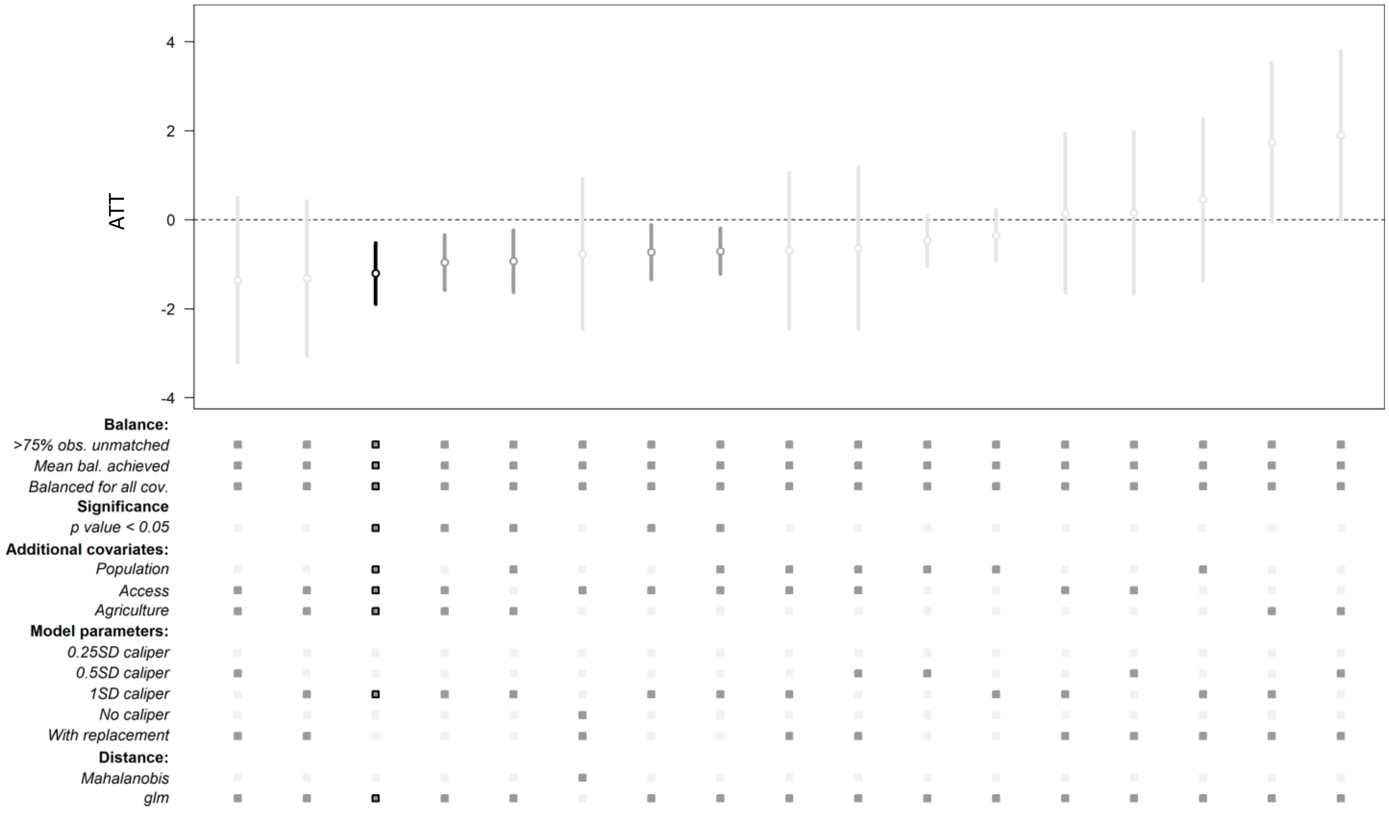

Supplementary Figure 7 Comparing results from the primary matching approach to 248 different matching model specifications for each matching treatment and corresponding outcome variables (A-I). The result from the primary matching approach is shown in black. For all comparison models, those where there was a significant difference between treatments and controls are shown in dark grey and those with no significant difference are shown in light grey. Average Treatment Effect on the Treated (ATT) were estimated using covariate-adjusted linear regression on the matched samples, incorporating matching weights and subclass-clustered robust standard errors. Statistical significance of treatment–control differences was assessed using two-sided Wald z-tests of the treatment coefficient. Results from models which did not achieve sufficient balance (standardised mean difference for all covariates <0.25) or sample size (at least 75% of treatment units matched) during matching were removed from the figures to reduce noise.

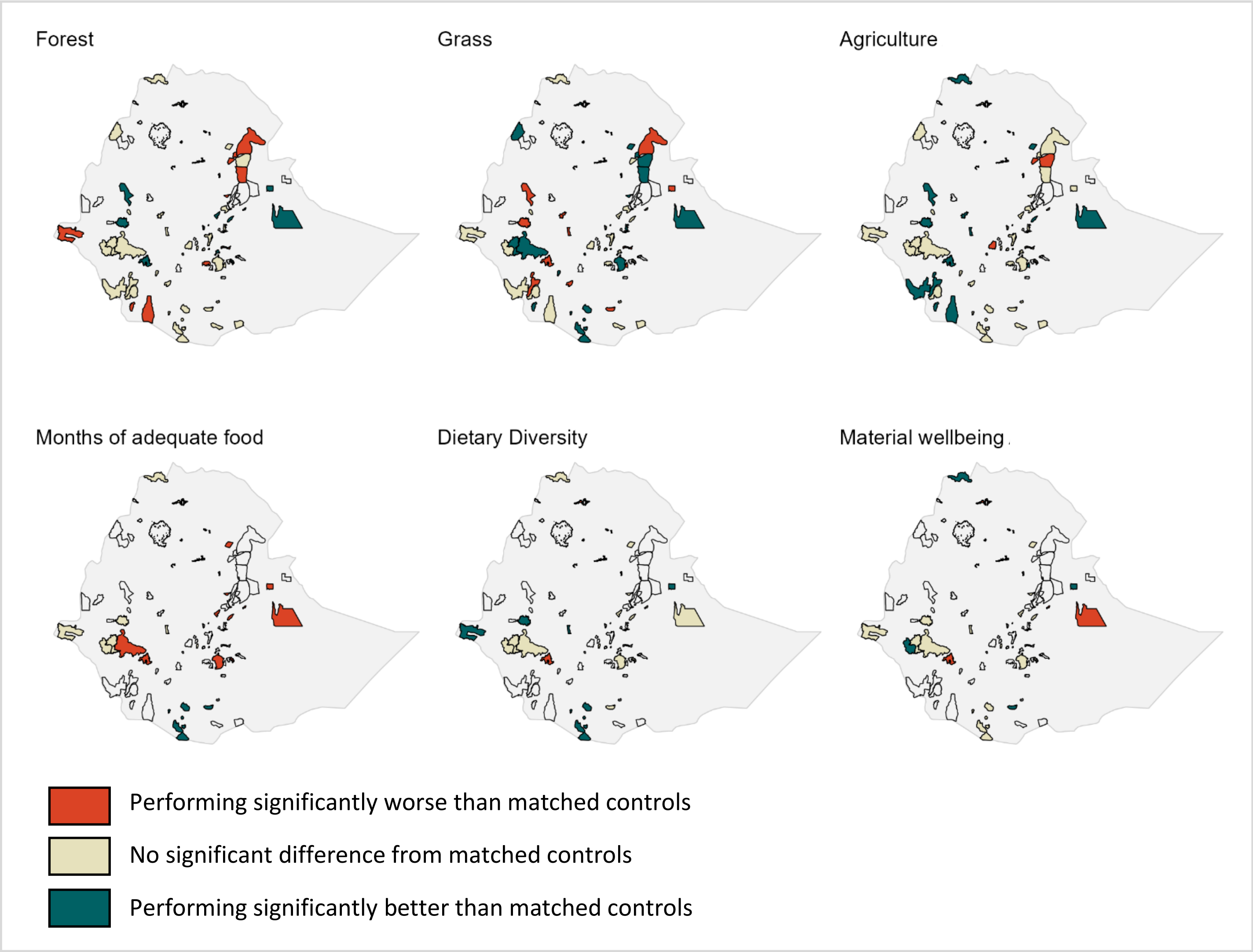

Supplementary Figure 8 Maps showing the performance of each protected area compared to matched controls. A protected area is found to be performing better than matched controls if its average treatment effect (ATT) is significantly positive (agriculture ATTs were inverted to conform with this), and worse if the ATT is significantly negative.

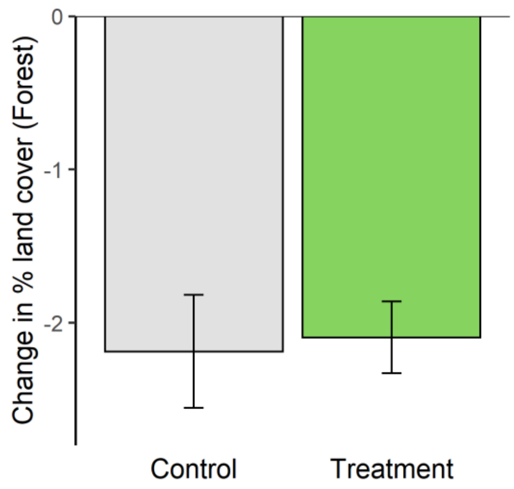

Supplementary Figure 9 National Forest Priority Area (NFPA) counterfactual analysis comparing forest loss from 2000-2021 within NFPAs and in matched control areas outside of both NFPAs and protected areas

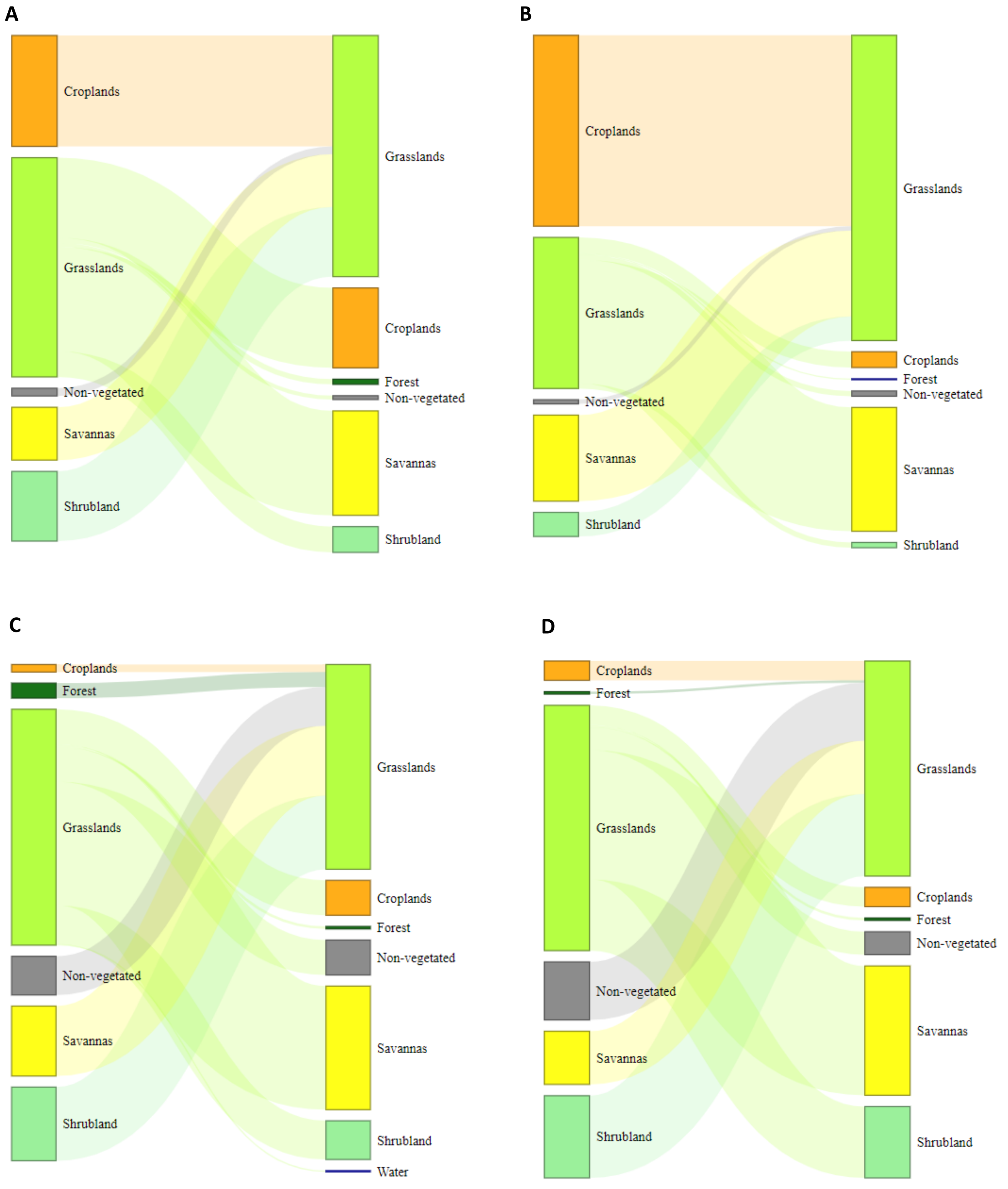

Supplementary Figure 10 Sankey diagrams showing landcover changes from and to grassland (2001-2020) in statistically matched units (A) treatment units for strict PAs, (B) control units for strict PAs, (C) treatment units for less strict PAs, and (D) control units for less strict PAs. Gridcells which remained grassland and gridcells which changed between land cover types that were not grassland were excluded for easier interpretation.

Supplementary tables

Supplementary Table 1 List of protected areas removed and added cleaning the World Database of Protected Areas dataset for Ethiopia with updated information from the Ethiopian Wildlife Conservation Authority, along with their metadata and the reason for the change.

Name	Designation	IUCN	Additional information
**Removed PAs – degazetted**			
Akobo	Controlled hunting area	VI	5870 km^2
Awash West	Controlled hunting area	VI	11821 km^2
Awash West	Wildlife Reserve	IV	1494 km^2
Boyo swamp	Controlled hunting area	VI	324 km^2
Dabus valley	Controlled hunting area	VI	1909 km^2
Donkoro Chaka	National Park	II	78 km^2
Eastern Hararghe	Controlled hunting area	VI	33009 km^2
Mizan-Teferi	Controlled hunting area	VI	3146 km^2
Segen Valley	Controlled hunting area	VI	396 km^2
Tedo	Controlled hunting area	VI	2379 km^2
**Removed PAs – encompassed into another PA**			
Omo West	Controlled hunting area	VI	Encompassed into Omo (Omo expanded from 3885 – 5144 km^2)
Yabello	Sanctuary	II	Encompassed into Borena (Borena downsized from 45821 – 3726 km^2)
**Removed PAs – duplicate**			
Simien National Park	World heritage site	VI	Duplicate of Simien Mountains National Park
**Added PAs - gazetted**			
Abune Yosef Zigit Abohoy Gara	Community Conservation Area	VI	80 km^2, gazetted in 2014
Anole Amude	Controlled Hunting Area	VI	102 km^2, gazetted in 2018
Bakusa	National Park	II	518 km^2, gazetted in 2012
Choke Mountain	Community Conservation Area	VI	60 km^2, gazetted in 2011
Godebie	National Park	II	186 km^2, gazetted in 2017
Guna Mountain	Community Conservation Area	VI	46 km^2, gazetted in 2016
Tana	UNESCO Biosphere Reserve		6972 km^2, gazetted in 2015
Majang	UNESCO Biosphere Reserve		2258 km^2, gazetted in 2017
Mahbere Silasie	Community Conservation Area	VI	191 km^2, gazetted in 2017
Menze Guassa	Community Conservation Area	VI	77 km^2, gazetted in 2011
Sheka	UNESCO Biosphere Reserve		2334 km^2, gazetted in 2012
Yayu	UNESCO Biosphere Reserve		1670 km^2, gazetted in 2010
**Added PAs – missing in WDPA**			
Shedeme Berbere	Controlled Hunting Area	VI	183 km^2, gazetted in 1988
Borena Sayint Worehimano	National Park	II	152 km^2, gazetted in 2007

Supplementary Table 2 Measures of effectiveness. Table showing the three environmental (forest, grassland and agricultural land cover change) and three social wellbeing (months of adequate food, dietary diversity, material wellbeing) outcome variables used to understand protected area effectiveness. All outcome variables were calculated as a change over their corresponding time period. Expectation refers to the expected direction of change for PAs compared to matched counterfactuals in the case the PAs are effective.

Outcome variable	Dataset	Time	Details	Expectation
**Forest** cover change	Global Forest Change v 1.9 datasets: *treecover2000* and *lossyear* [31,32]	2000-2021	Using the *treecover2000* dataset, we defined each 30 x 30 m pixel as forested if they had >40% tree coverage, where trees were defined as vegetation taller than 5 m. Then using the *lossyear*, we identified pixels which remained forested in 2021. Binary values for pixels were then aggregated across 1 x 1 km gridcells to calculate percentage forest cover in 2000 and in 2021.	Less decrease
**Grassland** cover change	MODIS V6 [11]	2000-2020	Grassland is defined as land cover dominated by herbaceous annuals (<2 m) and was based on University of Maryland land cover classifications at 500 m resolution. Datasets for 2000 and 2020 were aggregated across 1 x 1 km gridcells to calculate percentage grassland cover in both years.	Greater increase or less decrease (see Supplementary Methods S2 and Supplementary Results S2)
**Agricultural** land cover change	Global Land Analysis and Discovery [2]	2000-2019	Agricultural land cover is defined as land dominated by annual or perennial herbaceous crops at 30 x 30 m resolution. Using datasets for 2000-2003 and 2016-2019, binary values for pixels were then aggregated across 1 x 1 km gridcells to calculate percentage agricultural land cover in both years.	Less increase
**Months of Adequate Household Food Provisions** (months of adequate food) change	World Bank Living Standards Measurement Study (LSMS), Ethiopian Socioeconomic Surveys [29,30]. (Further information is available in Supplementary methods)	2011-2016	MAHFP calculated from panel data in 2011 and 2016 as the number of months in the year the household had enough food to eat [33].	Greater increase or less decrease
**Household Dietary Diversity Score** (dietary diversity) change			HDDS calculated from panel data in 2011 and 2016 as the number of different food items (from a list of 16 items shown in Supplementary Table 3) consumed by the household in the week prior to the survey [33].	Greater increase or less decrease
**Material wellbeing** status (proxied through a weighted asset ownership score) change			Material wellbeing was proxied through weighted asset ownership scores were calculated from panel data in 2011 and 2016 for each household. This was based on the number of different assets they owned (from a list 24 asset items shown in Supplementary Table 4), weighted using a principal component analysis [34]	Greater increase or less decrease

Supplementary Table 3 Food items considered in determining household dietary diversity status

**Food item**
Enjera (teff)
Other cereal (e.g., rice/sorghum/millet/wheat)
Potatoes
Pasta
Sugar/sugar products (e.g., honey/jam)
Beans/lentils/nuts
Vegetables
Fruits
Beef/sheep/goat/pork
Poultry
Eggs
Fish
Oils/fats/butter
Dairy (e.g., milk/yoghurt/cheese)
Condiments (e.g., spices/salt/pepper)
Kocho/Bula (enset)

Supplementary Table 4 Asset items for calculating material wellbeing

**Asset**
Bicycle
Cart (animal drawn)
Cart (hand pushed)
CD/VCD/DVD/Video Deck
Fixed line telephone
Gabi (blanket)
Gejera (machete)
Geso (pick axe)
Kerosene stove
Machid (sickle)
Mattress and/or bed
Mitad (flatbread stove)
Mobile telephone
Plough (modern)
Plough (traditional)
Radio
Satellite Dish
Shelf for storing goods
Sofa set
Television
Wardrobe
Water pump
Water storage pit
Wrist watch/clock

Supplementary Table 5 Covariates used for statistical matching relating to each directed acyclic graph (DAG) in Extended Data Fig 2 A and B. Data for all covariates resampled to 1km resolution for gridcell matches and to a 2km buffer around each household for household matches. Further information on the theory for including each covariate is included in Supplementary Table 6.

DAG	Covariate	Description	Data type	Unit	Data source
A & B	**ELEVATION**	Altitude	Continuous	m.a.s.l	Global 3- Arc-Second Elevation (GTOPO30) [4]
A & B	**SLOPE**	Slope	Continuous	Degrees	Global 3- Arc-Second Elevation (GTOPO30) [4]
A & B	**TEMPERATURE**	Average annual temperature (1981-2010)	Continuous	°C	Climatologies at high resolution for the earth's land surface areas (CHELSA) [5]
A & B	**PRECIPITATION**	Average annual rainfall (1981-2010)	Continuous	kg/m^2	Climatologies at high resolution for the earth's land surface areas (CHELSA) [5]
A & B	**AGRICULTURAL SUITABILITY**	Historical average suitability based on rainfed conditions (1980-2009)	Continuous		[35]
A	**ECOREGION**	Dominant ecoregion type	Categorical		[36]
A & B	**ETHNO-LINGUISTIC GROUPS**	First component of a PCA of proportion of people in each group	Continuous		[37]
A & B	**ACCESS**	Travel time to nearest city (>50,000 people) in 2000	Continuous	Minutes	Global Accessibility Map [3]
A & B	**POPULATION**	Population in 2000	Continuous	Number of people per $1km^2$ grid cell	[1]
A	**FOREST**	Baseline forest cover in 2000	Continuous	%	[32]
A & B	**AGRICULTURE**	Baseline agricultural land in 2003	Continuous	%	Global Land Analysis and Discovery [2]
A	**GRASSLAND**	Baseline grassland in 2000	Continuous	%	MODIS V6 [38]
A	**LAND**	Majority land cover type in 2000	Categorical		MODIS V6 [38] – Reclassified into broader categories

Supplementary Table 6 Theory on how each confounding variable may influence both treatment and outcomes from literature review focused on the Ethiopian context. Darker colours indicate stronger expected impact on the outcome.

Covariate	Potential Effect on Treatment Selection	Potential effect on environmental outcomes			Potential effect on wellbeing outcomes		
		Forest Cover Change	Agricultural Land Cover Change	Grassland Cover Change	Months of Adequate Household Food Provisioning Change	Household Dietary Diversity Change	Asset Ownership Change
Elevation	PA locations are often biased towards high elevation areas, as this land is often less accessible and less able to be used for human settlement and agriculture [39]. Joppa and Pfaff [40] found elevation to be a significant and positive factor explaining protected area locations in Ethiopia.	Moderate impact. Higher elevations may have some protective effect for forest due to being less accessible for logging or less suitable for agriculture, but this varies based on local practices [41]. In coffee growing regions, deforestation is greater at high elevations as they are not suitable for coffee growth, while lower elevations are somewhat protected due to providing shade for coffee plants [42].	Moderate impact. Lower elevations are often preferred for agriculture [43] due to improved soil conditions and access to markets so agricultural expansion may be more likely.	Minimal impact. Grasslands at lower elevations may be more vulnerable to conversion to agriculture [43].	Minimal impact. Households at higher elevations are likely to have greater travel time to markets and fewer alternative livelihood options, which may impact food provisioning [44].	Moderate impact. Farm crop composition and diversity has been shown to be influenced by elevation, and this is likely to be closely related household dietary diversity, particularly in rural areas where subsistence farming is a common livelihood [45].	Minimal impact. Households at higher elevations are likely to have greater travel time to markets and fewer alternative livelihood options, which may impact how much income they can generate for asset accumulation [44].
Slope	PAs are also often biased towards steeper slopes as these areas are often less accessible and have fewer opportunity costs for alternative uses such as agriculture [40].	Moderate impact. Steep slopes resist deforestation due to difficulty in clearing; low slopes are more easily converted to other land uses and preferable for agriculture [41,42].	Moderate impact. Steep slopes limit agriculture and grazing due to reduced accessibility, reducing expansion [43].	Minimal impact. Flat terrain may promote grassland conversion for agricultural purposes [43].	Moderate impact. Slope has been shown to impact soil nutrients in Southern Ethiopia with significantly lower organic carbon on total nitrogen in lower slopes [46]. This in turn influences food production and food security.	Minimal impact. Farmer decisions regarding on farm crop diversity are influenced by slope [45].	Minimal impact. Slope has been shown to impact soil nutrients in Southern Ethiopia with significantly lower organic carbon on total nitrogen in lower slopes [46]. This in turn influences food production and production income.
Precipitation (1981-2010)	Areas with high precipitation tend to support denser forests and biodiversity which may make them more likely to be established as PAs [39]. Species richness was found to be significantly positively associated with PA placement in Ethiopia [40].	Moderate impact. Wetness influences agricultural suitability which in turn can influence likelihood of forest clearance for agriculture [41], particularly as much forest disturbance is driven by smallholder agriculture which is more reliant on rain [47].	Strong impact. Wetness influences agricultural suitability which in turn can influence likelihood of agricultural expansion [41], particularly as subsistence farming is the most common livelihood in Ethiopia and is more reliant on rain [47].	Moderate impact. Grasslands in moderately wet regions may be targeted for agriculture, while very arid areas are less likely to be converted [41,48].	Moderate impact. Higher precipitation can increase crop productivity, improving food provisioning particularly as the majority of agriculture in Ethiopia is rain-fed [49].	Moderate impact. Higher historical average rainfall is positively correlated with crop diversity on Ethiopian farms [50] which impacts dietary diversity	Minimal impact. Higher precipitation can increase crop productivity, improving yields and available product to sell for additional income [49].
Temperature (1981-2010)	Temperature influences vegetation types as well as suitability of areas for agriculture. Both of these are likely to influence whether a PA is designated in the area [39].	Moderate impact. In Ethiopia, extreme high temperatures are associated with crop damage and a farmer response to deforest and expand agriculture for food security [51].	Moderate impact. Where high temperatures reduce crop yield, agricultural expansion may occur to maintain food production levels [51,52].	Minimal impact. Temperatures will impact suitability for cropland expansion, and so areas with temperatures more suited for agriculture may be more likely to be converted from grassland to agriculture [53].	Moderate impact. Extreme high temperatures in Ethiopia have been found to be associated with increase crop damage and reduced crop production and value [51].	Minimal impact. historical mean temperatures are associated with lower crop diversity on Ethiopian farms [50] which in turn influences dietary diversity.	Minimal impact. Higher temperatures have been shown to have a negative relationship with agricultural yields and income [54].
Agricultural Suitability	PAs are often biased towards areas less suitable for agriculture as there are fewer opportunity costs for alternative uses [40].	Moderate impact. Greater agricultural suitability can influence the likelihood of forest clearance for agriculture [41].	Strong impact. Agricultural suitability can influence likelihood of agricultural expansion [41].	Moderate impact. Greater agricultural suitability can influence the likelihood of grassland conversion to agriculture [41].	Strong impact. Agricultural suitability takes into account a variety of soil properties. Households living in areas with soil fertility are more likely to be able to produce adequate food for consumption [55].	Moderate impact. Soil fertility (an important component of agricultural suitability) has a statistically significant negative relationship with crop diversification in Ethiopia, with farmers more likely to have more diverse crops on more degraded less suitable land [56].	Moderate impact. suitability takes into account a variety of soil properties. Rural households living in areas with soil fertility are more likely to be able to produce higher yields which may increase the amount of products that can be sold on the market to generate additional income [57]. People leaving in areas with low agricultural potential have also been found to be more marginalised [58].

Ecoregion	Certain ecoregions (e.g., forests) may be prioritised in PA designation due to their respective conservation value. High biodiversity areas are a priority in Ethiopia's NBSAP [59].	Moderate impact. An area dominated by a non-forest ecoregion type at baseline is less likely to experience changes in forest cover.	Moderate impact. Ecoregion type affects suitability for agriculture conversion [60].	Moderate impact. Ecoregion type influences how likely it is an area can convert to or from grassland. E.g., savanna/ or shrubland is more associated with grassland changes [60].	Minimal impact.	Minimal impact.	Minimal impact.
Ethnolinguistic group	Ethiopia's ethnolinguistic groups have generally determined administrative region borders. Many of Ethiopia's protected areas are managed by regional rather than federal authorities. Regional authorities can request designation of new protected areas, and therefore locations may depend on priorities of different ethnolinguistic groups.	Moderate impact. Different groups have different cultural practices - e.g. pastoralism, strong or weak utilisation from the forest. Regional states have their own proclamations related to forestry and its sustainable use and conservation [59]	Moderate impact. Different groups have different cultural practices - e.g. pastoralism, strong or weak utilisation from the forest. Regional policies can influence expansion. For example land rental duration and size depends on the laws of the respective regional state [61].	Moderate impact. Different groups have different cultural practices - e.g. pastoralism, strong or weak utilisation from the forest. According to Ethiopia's NBSAP [59] several regions are integrating measures to rehabilitate degraded montane grasslands.	Moderate impact. Different ethnolinguistic groups in Ethiopia have different indigenous agrisystems and cultures relating to food production, which can have impacts on food security [62]. For example, households in enset-based agrisystems have been found to be less impacted by drought and receive less food aid than other farming systems in Ethiopia [63,64].	Moderate impact. Ethiopia's ethnolinguistic groups closely align with administrative regions. Dietary diversity has been shown to significantly differ across regions which may be related to access to diverse food markets, employment opportunities of cultural habits for consuming diverse foods [65].	Moderate impact. Ethnic diversity and ethnicity have been found to be important factors in explaining differences in wealth [66]. Additionally, hotspots of marginality in Ethiopia are more homogenous in terms of ethnic group [58].
Access (travel time to major city, 2000)	Remote areas are more likely to be protected due to lower economic opportunity costs. Joppa and Pfaff [40] found distance to urban areas to be significantly positively associated with PA placement in Ethiopia.	Strong impact. More accessible areas often experience more deforestation due to reduced travel time to markets which can boost agriculture conversion and logging [47].	Strong impact. Better access can promote expansion of large scale agriculture as it reduces transportation costs to markets and less accessible areas may have higher costs of clearing the land therefore reducing agricultural expansion [52].	Moderate impact. Accessible grasslands may be at risk of conversion, but this depends on local demand for land [67].	Moderate impact. Access to towns in Ethiopia is negatively associated with total cultivated area which is likely to impact food provisioning ability [68]. Remote areas might also experience reduced food provisioning due to limited market access [44].	Minimal impact. Limited access to urban markets in remote areas has been shown to reduce dietary diversity in Ethiopia [44].	Strong impact. Positive relationships between access to markets and household wellbeing have been found in Ethiopia [44].
Population (2000)	Areas with high human populations are generally more developed with land dedicated to alternative uses making them less likely to be established as protected areas due to opportunity costs and social conflicts [39].	Strong impact. Higher population densities are often in towns and cities with greater possibilities to generate income through non-farming related jobs so are less associated with forest clearance for agriculture [42]. Lower population densities were found to lead to higher forest loss in the Albertine rift [69].	Strong impact. Population density is likely to affect the demand for farmland [52]. In areas close to cities it may reduce agricultural expansion as people switch to alternative livelihoods, whereas in less accessible areas it may lead to expansion to meet subsistence demands [42].	Moderate impact. High population densities are likely to be associated with increased livestock grazing promoting bush encroachment on grasslands [70].	Moderate impact. In rural areas of Ethiopia, higher population density is associated with smaller farm sizes and lower farm income per hectare [71] which is likely to reduce food security.	Minimal impact. High populations are often found in urban areas. Living in an urban area has been found to be positively associated with household dietary diversity in Ethiopia [72].	Moderate impact. High population areas are often found in urban locations, and poverty status and change are driven by different factors in urban compared to rural areas in Ethiopia [73].
Baseline Forest Cover (2000)	High baseline forest cover areas may be prioritised for protection to conserve forest ecosystems, biodiversity, and carbon stocks and are considered priorities for conservation in Ethiopia's National Biodiversity Strategic Action Plan [59].	Moderate impact. More intact forest is likely to be less accessible and therefore less targeted for logging and clearance for agricultural activities [47].	Moderate impact. Lower baseline forest cover may indicate fragmented landscapes which are more likely to be targeted for agricultural expansion [74].	Minimal impact. Areas with high baseline forest cover are unlikely to undergo grassland changes.	Minimal impact.	Minimal impact.	Minimal impact.
Baseline Grassland Cover (2001)	Grassland-rich areas may be chosen for PAs to preserve unique ecosystems and biodiversity.	Minimal impact.	Moderate impact. Higher baseline grassland cover may indicate greater suitability of land for agriculture conversion [75].	Moderate impact. Baseline grassland cover affects how much grass can be gained/lost.	Minimal impact.	Minimal impact.	Minimal impact.
Baseline Agriculture (2000)	Areas with minimal agriculture are more likely to be established as PAs to avoid disrupting land use and economic opportunities. Joppa and Pfaff [40] found agricultural suitability to be significantly negatively associated with PA placement in Ethiopia.	Minimal impact. Forest areas fragmented with agriculture are more likely to be targets for further conversion to agriculture [74].	Strong impact. Baseline agricultural land coverage affects how much agricultural expansion can occur.	Minimal impact. Areas with high baseline agriculture may have more potential for grassland restoration.	Moderate impact. Areas under large scale farming have been shown to reduce local household food security in several regions of Ethiopia due to limiting land available for smallholders, outsourcing of employees and export of food into national and international markets [76,77].	Minimal impact. Small (less intensive) farms have been shown to have higher crop richness than larger farms, which may affect dietary diversity [78].	Moderate impact. Village level crop areas in Ethiopia are positively correlated with farm-related income which will impact asset accumulation [79].

Supplementary Table 7 Semivariance produced at different sampling densities for gridcells

Distance between sampled units (km)	Mean semivariance	Maximum semivariance	Minimum semivariance
0	24.34	51.59	2.46
2	2.09	3.30	2.58
4	2.97	3.21	2.45
6	3.13	3.34	2.65

Supplementary Table 8 Involvement of external project funding or non-governmental organisations (NGOs) with protected areas. This compiles accessible information available for each protected area on whether it was associated with any external funding between 2000 and 2020.

Protected Area	NGO present	NGO names
Abasheba Demero	No	
Abjata Shala Lakes	Yes	Farm Africa; Wetlands International; United Nations Development Program (Global Environment Fund)
Abune yosef Zigit Abohoy Gara	No	
Adaba Dodola	Yes	GIZ
Afdem-Gewane	No	
Alitash	Yes	United Nations Development Program (Global Environment Fund)
Aluto	No	
Amibera-Melika sadi	No	
Anole Amude	No	
Arba Gugu	No	
Arsi Mountains	Yes	Climate Chance, Rufford Foundation
Asibahri Kebena	No	
Awash	Yes	United Nations Development Program (Global Environment Fund), GIZ
Babile Elephant	Yes	United Nations Development Program (Global Environment Fund)
Bakusa	No	
Bale Mountains	Yes	United Nations Development Program (Global Environment Fund); Frankfurt Zoological Society; Ethiopian Wolf Conservation Programme
Bejmiz	No	
Beroye	No	
Besemena Odo-bulu	No	
Billen-Hertale	No	
Borena	Yes	SOS Sahel International
Borena sayint Worehimano	Yes	Ethiopian World Conservation Programme
Chebera Churchura	Yes	GIZ; Global Environment Fund
Chelbi	No	
Chifra	No	
Choke Mountain	Yes	Global Environment Fund
Deddessa	No	
Dembel Ayisha Adigala	No	
Dhati Welel	No	
Dindin	No	
Erer-Gota	No	
Gambella	Yes	United Nations Development Program (Global Environment Fund); African Parks
Gara Gumbi	No	
Gara Meti	No	
Gassera Wabe	No	
Gelila Dura	No	
Geralle	Yes	United Nations Development Program (Global Environment Fund);

Gewane	Yes	CARE climate change
Gibe Sheleko	No	
Godebie	No	
Guna Mountain	Yes	Global Environment Fund
Hadar	No	
Hallaydeghe-Asebot	Yes	United Nations Development Program (Global Environment Fund); KFW
Hanto	No	
Haro Aba Diko	No	
Hurufa-Soma	No	
Jibat	No	
Kafa	Yes	NABU
Kafta Sheraro	Yes	United Nations Development Program (Global Environment Fund)
Liban Plain	No	
Loka Abaya	Yes	SOS Sahel International
Mago	Yes	Global Environment Fund
Mahbere Silasie	No	
Majang	Yes	Swedish International Development Cooperation Agency (Farm Africa, TechnoServe and MELCA-Ethiopia)
Mao-Komo	No	
Maze	No	
Melka Guba	No	
Menze Guassa	No	
Milleserdo	No	
Munessa Ambagoda-Sade	No	
Munessa Kuke	No	
Murulle	No	
Nanigadhera	No	
Nech Sar	Yes	United Nations Development Program (Global Environment Fund); GIZ
Omo	Yes	United Nations Development Program (Global Environment Fund)
Senkele Swaynes Hartebeast	Yes	United Nations Development Program (Global Environment Fund), Farm Afric;
Shedeme Berbere	No	
Sheka	Yes	NABU
Shinele Meto	No	
Simien Mountains	Yes	United Nations Development Program (Global Environment Fund;, KFW; AWF
Sororo Torgam Gara Muktar	No	
Tama	No	
Tana	Yes	NABU
Telalak Dewe	No	
Tulu Lafto-Sedden	No	
Urgan-Bula	No	
Weyib Valley	No	
Yangudi Rassa	Yes	United Nations Development Program (Global Environment Fund)
Yayu	Yes	Critical Ecosystem Partnership Fund (CEPF); NABU

Supplementary Table 9 Demographic information for questionnaire respondents. N is number of respondents and % is the percentage of respondents.

Demographic	N	%
**Age**		
<21	0	0.0
21-30	1	2.7
31-40	15	40.5
41-50	11	29.7
51-60	9	24.3
61-70	0	0.0
>70	1	2.7
**Sex**		
Male	32	86.5
Female	5	13.5
**Education**		
Primary	1	2.7
Secondary	0	0.0
Bachelors	6	16.2
Masters	21	56.8
PhD	9	24.3
**Organisation**		
Research	5	11.6
NGO	6	14.0
Private	2	4.7
Government	30	69.8

Supplementary Table 10 Updated full list of protected areas and associated metadata. Earliest year represents the earliest record (to the best of the Ethiopian Wildlife Conservation Authorities knowledge) of the protected area either regionally or nationally, including if the area used to be under a different type of protection or different name, rather than the date it was designated on the World Database of Protected Areas. Budget group indicates whether the budget fell within the bottom quartile (Low), interquartile range (Mid) or upper quartile (High). True budget data can be obtained from the Ethiopian Wildlife Conservation Authority. This dataset represents the protected area shapefile in September 2024, this is continually being updated.

Name	Designation	IUCN	Area (km^2)	Earliest Year	Budget
Abasheba Demero	Control Hunting Area	VI	178	1994	Low
Abjata Shala Lakes	National Park	II	813	1963	High
Abune yosef Zigit Abohoy Gara	Community Conservation Area	VI	81	2014	Low
Adaba Dodola	Control Hunting Area	VI	514	2000	High
Afdem-Gewane	Control Hunting Area	VI	4718	1973	Low
Alitash	National Park	II	2667	1997	High
Aluto	Control Hunting Area	VI	89	2006	Low
Amibera-Melika sadi	Control Hunting Area	VI	111	1973	Low
Anole Amude	Control Hunting Area	VI	102	1973	Low
Arba Gugu	Control Hunting Area	VI	338	1995	Low
Arsi Mountains	National Park	II	930	1973	Mid
Asibahri Kebena	Control Hunting Area	VI	167	1973	Low
Awash	National Park	II	590	1958	High
Babile Elephant	Sanctuary	IV	8804	1962	High
Bakusa	National Park	II	518	2012	Mid
Bale Mountains	National Park	II	2148	1962	High
Bejmiz	National Park	II	1836	2015	Low
Beroye	Control Hunting Area	VI	356	2013	Low
Besemena Odo-bulu	Control Hunting Area	VI	242	1930	Mid
Billen-Hertale	Control Hunting Area	VI	825	1973	Low
Borena	National Park	II	3724	1966	High
Borena sayint Worehimano	National Park	II	152	1930	High
Chebera Churchura	National Park	II	1265	1997	High
Chelbi	Wildlife Reserve	IV	4303	1973	Low
Chifra	Control Hunting Area	VI	545	1998	Low
Choke Mountain	Community Conservation Area	VI	60	2011	Low
Deddessa	National Park	II	2343	1970	Low
Dembel Ayisha Adigala	Control Hunting Area	VI	908	2010	Low
Dhati Welel	National Park	II	1040	2010	Mid
Dindin	Control Hunting Area	VI	286	1992	High
Erer-Gota	Control Hunting Area	VI	2630	1973	Low
Gambella	National Park	II	4621	1966	High
Gara Gumbi	Open Hunting Area	VI	49	1973	Low
Gara Meti	Open Hunting Area	VI	319	1973	Low
Gassera Wabe	Control Hunting Area	VI	230	1964	Low
Gelila Dura	Open Hunting Area	VI	52	1973	Low
Geralle	National Park	II	1767	1974	High
Gewane	Wildlife Reserve	IV	3008	1973	Low
Gibe Sheleko	National Park	II	321	1930	Low
Godebie	National Park	II	187	2016	Mid

Guna Mountain	Community Conservation Area	VI	46	2016	Mid
Hadar	Control Hunting Area	VI	377	1973	Low
Hallaydeghe-Asebot	National Park	II	1099	1973	Mid
Hanto	Control Hunting Area	VI	206	1991	Mid
Haro Aba Diko	Control Hunting Area	VI	244	2000	Low
Hurufa-Soma	Control Hunting Area	VI	231	2000	High
Jibat	Control Hunting Area	VI	367	1988	Mid
Kafa	Biosphere Reserve	VI	7406	1973	Low
Kafta Sheraro	National Park	II	2196	1999	High
Liban Plain	Sanctuary	IV	97	1930	Low
Loka Abaya	National Park	II	546	2001	Low
Mago	National Park	II	1870	1971	Mid
Mahbere Silasie	Community Conservation Area	VI	191	2016	Mid
Majang	Biosphere Reserve	VI	2258	1973	Mid
Mao-Komo	National Park	II	2320	2016	Low
Maze	National Park	II	202	1983	High
Melka Guba	National Park	II	547	1930	Low
Menze Guassa	Community Conservation Area	VI	77	1600	Mid
Milleserdo	Wildlife Reserve	IV	7305	1973	Low
Munessa Ambagoda-Sade	Control Hunting Area	VI	163	1963	Mid
Munessa Kuke	Control Hunting Area	VI	110	1993	High
Murulle	Control Hunting Area	VI	504	1973	Low
Nanigadhera	Control Hunting Area	VI	192	2018	Low
Nech Sar	National Park	II	415	1966	High
Omo	National Park	II	5160	1959	Mid
Senkele Swaynes Hartebeast	Sanctuary	IV	53	1964	High
Shedeme Berbere	Control Hunting Area	VI	184	1988	Mid
Sheka	Biosphere Reserve	VI	2334	1973	Mid
Shinele Meto	Control Hunting Area	VI	641	2000	Low
Simien Mountains	National Park	II	411	1959	High
Sororo Torgam Gara Muktar	Control Hunting Area	VI	73	2000	Mid
Tama	Community Conservation Area	IV	1948	1973	High
Tana	Biosphere Reserve	VI	6972	2011	Mid
Telalak Dewe	Control Hunting Area	IV	503	1972	Low
Tulu Lafto-Sedden	Sanctuary	IV	563	1988	Mid
Urgan-Bula	Control Hunting Area	VI	78	2000	Low
Weyib Valley	Control Hunting Area	VI	350	2013	Low
Yangudi Rassa	National Park	II	3047	1969	Mid
Yayu	Biosphere Reserve	VI	1670	1988	High

Supplementary Table 11 Number of species with range overlapping each protected area

Protected area	Birds	Herptiles	Mammals	Plants	Total
Abasheba Demero	369	51	83	217	720
Abjata Shala Lakes	476	58	90	277	901
Abune Yosef Zigit Abohoy Gara	278	29	58	121	486
Adaba Dodola	340	63	88	237	728
Afdem-Gewane	443	57	86	222	808
Alitash	298	29	67	121	515
Aluto	449	54	88	259	850
Amibera-Melika sadi	438	57	87	204	786
Anole Amude	455	58	91	274	878
Arba Gugu	375	56	80	237	748
Arsi Mountains	465	70	107	302	944
Asibahri Kebena	416	49	84	168	717
Awash	457	63	85	229	834
Babile Elephant	373	77	80	257	787
Bakusa	306	29	66	116	517
Bale Mountains	393	69	94	242	798
Bejmiz	312	29	68	120	529
Beroye	370	54	87	202	713
Besemena Odo-bulu	361	52	85	220	718
Billen-Hertale	427	50	83	183	743
Borena	468	78	94	291	931
Borena sayint Worehimano	320	36	66	138	560
Chebera Churchura	429	62	99	287	877
Chelbi	459	78	116	243	896
Chifra	295	30	53	121	499
Choke Mountain	320	45	74	144	583
Deddessa	386	55	89	222	752
Dembel Ayisha Adigala	321	69	52	159	601
Dhati Welel	331	43	74	173	621
Dindin	378	57	77	215	727
Erer-Gota	411	57	74	230	772
Gewane	355	44	77	151	627
Gambella	348	68	95	169	680
Gara Gumbi	430	56	81	210	777
Gara Meti	459	60	87	239	845
Gassera Wabe	374	48	73	218	713
Gelila Dura	327	36	73	131	567
Geralle	304	76	62	185	627
Gibe Sheleko	421	56	78	253	808
Godebie	272	26	53	117	468
Guna Mountain	309	35	65	134	543
Hadar	285	33	59	115	492
Hallaydeghe-Asebot	444	60	88	208	800
Hanto	350	55	82	215	702
Haro Aba Diko	360	55	86	224	725
Hurufa-Soma	356	56	87	221	720
Jibat	407	55	80	252	794
Kafa	457	74	109	335	975
Kafta Sheraro	327	29	58	147	561
Liban Plain	323	59	62	211	655
Loka Abaya	472	63	98	270	903

Mago	445	73	117	225	860
Mahbere Silasie	319	29	55	130	533
Majang	403	61	100	236	800
Mao-Komo	327	46	74	156	603
Maze	424	65	97	246	832
Melka Guba	354	68	63	208	693
Menze Guassa	326	33	67	139	565
Milleserdo	312	38	61	121	532
Munessa Ambagoda-Sade	433	54	93	269	849
Munessa Kuke	391	55	86	265	797
Murulle	403	71	108	181	763
Nanigadhera	343	50	71	215	679
Nech Sar	472	72	102	264	910
Omo	463	75	117	228	883
Senkele Swaynes Hartebeast	443	58	89	246	836
Shedeme Berbere	371	51	85	219	726
Sheka	412	67	99	273	851
Shinele Meto	367	59	64	221	711
Simien Mountains	343	37	67	179	626
Sororo Torgam Gara Muktar	355	59	71	202	687
Tama	464	71	119	258	912
Tana	433	55	81	184	753
Telalak Dewe	306	33	63	127	529
Tulu Lafto-Sedden	371	48	86	240	745
Urgan-Bula	345	55	80	237	717
Weyib Valley	367	54	77	213	711
Yangudi Rassa	311	37	66	124	538
Yayu	382	60	91	250	783

Supplementary Table 12 Matching is robust to the presence of an unobserved confounding variable. Outputs from sensitivity analyses conducted using the R package *Sensemakr* for each outcome variable in each matching group. The robustness value (RV) represents the percentage of the residual variance of both the treatment and the outcome that an unobserved confounder would need to explain to bring the estimated effect to zero. This is then compared to a benchmark covariate, for environmental outcomes we used population size as the benchmark, and for social outcomes we used agricultural suitability. We only tested these bounds up to 9 times the strength of the benchmark.

**Matching group**	**Outcome variable**	**RV (%)**	**Benchmark covariate [RV (%)]**	**Explanatory power required by unobserved covariate to bring the outcome to zero, compared to the observed power of the benchmark**
Strict	Forest	2.81	Population [0.04]	> 9x
	Grassland	6.40		> 9x
	Agriculture	8.04		> 9x
Less Strict	Forest	NA	Population [0.08]	NA (effect is already zero)
	Grassland	3.37		> 9x
	Agriculture	8.84		> 9x
Household	Months of adequate food	2.35	Agricultural suitability [0.01]	> 9x
	Dietary diversity	NA		NA (effect is already zero)
	Material wellbeing	13.31		> 9x

Supplementary Table 13 Individual protected area environmental outputs showing the average treatment effect on the treated (ATT), t-statistic (t) and significance (p); significant p-values are shown in bold. For forest and grassland, a positive ATT indicates better performance while for agriculture a negative ATT indicates better performance.

Protected area	Forest			Agriculture			Grassland		
	ATT	t	p	ATT	t	p	ATT	t	p
Abasheba Demero (LS)	2.78	5.36	**<0.001**	-0.89	-0.26	0.796	-0.54	-0.12	0.906
Abjata Shala Lakes (S)	-0.20	-1.63	0.103	7.78	2.08	**0.038**	11.95	1.90	0.058
Adaba Dodola (LS)	-1.25	-2.14	**0.033**	-1.82	-1.48	0.138	-2.48	-0.59	0.558
Afdem Gewane (LS)	-0.02	-0.29	0.771	-0.35	-1.67	0.096	0.43	0.31	0.759
Alitash (S)	0.03	0.38	0.706	-0.08	-0.24	0.813	46.99	18.73	**<0.001**
Amibera Melika sadi (LS)	-0.42	-3.37	**0.001**	-0.56	-1.89	0.059	-54.63	-3.72	**<0.001**
Anole Amude (LS)	0.67	2.30	**0.022**	-1.54	-0.60	0.550	7.09	1.79	0.074
Arba Gugu (LS)	-0.28	-0.53	0.596	-2.67	-1.25	0.212	13.04	3.10	**0.002**
Arsi Mountains (S)	0.15	0.76	0.447	3.68	1.88	0.060	0.54	0.08	0.933
Asibahri Kebena (LS)	-0.59	-4.30	**<0.001**	-0.71	-1.87	0.061	8.38	2.45	**0.015**
Awash (S)	-0.06	-1.44	0.150	-0.19	-0.65	0.519	4.85	1.96	0.050
Babile Elephant (LS)	0.10	2.43	**0.015**	-2.76	-8.99	**<0.001**	3.43	4.11	**<0.001**
Bale Mountains (S)	0.52	0.58	0.564	1.96	1.42	0.155	7.56	3.03	**0.003**
Besemena Odo bulu (LS)	1.88	4.03	**<0.001**	-3.26	-2.72	**0.007**	-2.44	-0.61	0.540
Billen Hertale (LS)	-0.29	-2.76	**0.006**	-0.48	-2.00	**0.046**	5.41	2.31	**0.021**
Borena (S)	-0.18	-1.43	0.152	-0.38	-0.76	0.445	5.34	3.03	**0.002**
Chebera Churchura (S)	1.10	4.83	**<0.001**	-2.19	-2.70	**0.007**	-4.51	-2.10	**0.036**
Chelbi (LS)	-0.62	-4.95	**<0.001**	-2.17	-4.83	**<0.001**	1.69	1.13	0.258
Chifra (LS)	0.10	1.83	0.067	-0.56	-2.86	**0.004**	7.49	10.23	**<0.001**
Deddessa (S)	0.53	2.00	**0.046**	-2.99	-4.24	**<0.001**	-5.13	-3.24	**0.001**
Dindin (LS)	0.93	2.49	**0.013**	-7.22	-3.11	**0.002**	11.29	1.67	0.095
Erer Gota (LS)	0.09	1.21	0.225	-0.57	-3.21	**0.001**	7.11	5.10	**<0.001**
Gambella (S)	-0.21	-2.20	**0.028**	-0.09	-0.46	0.645	-3.68	-1.27	0.204
Gara Gumbi (LS)	-0.56	-4.05	**<0.001**	-1.46	-1.40	0.160	-31.48	-3.07	**0.002**
Gara Meti (LS)	0.36	2.62	**0.009**	-0.10	-0.17	0.862	-5.93	-0.99	0.324
Gassera Wabe (LS)	-1.07	-2.42	**0.016**	-1.36	-0.95	0.341	-13.80	-2.53	**0.011**
Gelila Dura (LS)	-0.17	-0.68	0.498	-0.49	-0.76	0.445	2.64	0.34	0.732
Geralle (S)	0.02	0.51	0.608	-0.60	-1.90	0.057	-4.82	-1.33	0.183
Gewane (LS)	-0.39	-4.14	**<0.001**	-0.30	-1.36	0.173	7.22	7.67	**<0.001**
Gibe Sheleko (S)	-0.17	-0.95	0.342	-7.00	-2.37	**0.018**	-18.67	-3.73	**<0.001**
Hadar (LS)	-0.42	-3.96	**<0.001**	-0.30	-1.12	0.263	7.22	9.49	**<0.001**
Hallaydeghe Asebot (S)	-0.09	-1.62	0.105	-0.22	-0.72	0.469	6.79	3.35	**0.001**
Hanto (LS)	1.24	2.62	**0.009**	-1.52	-1.11	0.267	-6.56	-1.13	0.259
Haro Aba Diko (LS)	-1.24	-0.87	0.382	1.68	0.61	0.543	0.43	0.26	0.796
Hurufa Soma (LS)	-1.39	-1.82	0.069	-1.71	-1.94	0.052	1.27	0.90	0.370
Jibat (LS)	0.51	1.37	0.171	-4.45	-2.64	**0.008**	-4.81	-2.47	**0.014**
Kafa (LS)	-0.39	-1.56	0.119	-0.45	-0.80	0.422	6.06	5.59	**<0.001**
Kafta Sheraro (S)	-0.07	-1.17	0.244	-0.68	-2.28	**0.023**	-4.63	-1.24	0.217
Liban Plain (LS)	0.20	1.36	0.174	11.27	0.91	0.364	0.92	0.15	0.880
Mago (S)	-0.07	-0.71	0.476	-0.61	-1.44	0.151	0.28	0.09	0.931
Majang (LS)	-0.88	-1.48	0.138	-0.24	-0.49	0.622	1.93	1.95	0.051
Maze (S)	-0.92	-1.22	0.221	-1.26	-0.96	0.339	31.95	3.25	**0.001**

Melka Guba (S)	0.03	0.48	0.634	0.70	1.66	0.097	-20.50	-3.70	**<0.001**
Menze Guassa (LS)	0.51	0.96	0.335	-8.33	-1.36	0.172	9.53	1.76	0.078
Milleserdo (LS)	-0.12	-3.06	**0.002**	0.33	1.73	0.084	-4.01	-3.79	**<0.001**
Munessa Ambagoda Sade (LS)	-1.44	-3.14	**0.002**	10.87	2.53	**0.011**	0.64	0.07	0.946
Munessa Kuke (LS)	-1.83	-0.70	0.481	6.19	0.95	0.345	10.55	1.31	0.189
Murulle (LS)	-0.31	-2.14	**0.033**	-2.11	-3.73	**<0.001**	14.57	6.88	**<0.001**
Nech Sar (S)	0.20	1.36	0.175	-1.08	-1.39	0.165	-24.96	-2.95	**0.003**
Omo (S)	0.13	1.37	0.170	-2.31	-4.69	**<0.001**	-2.09	-1.00	0.316
Senkele Swaynes Hartebeast (LS)	0.57	1.30	0.193	-1.89	-0.96	0.338	17.41	6.00	**<0.001**
Shedeme Berbere (LS)	0.02	0.05	0.961	-2.13	-2.78	**0.006**	-4.38	-2.05	**0.040**
Sheka (LS)	-0.72	-1.58	0.114	-0.50	-0.60	0.548	3.97	2.32	**0.020**
Shinele Meto (LS)	0.29	3.02	**0.003**	-0.30	-0.97	0.331	-11.42	-3.78	**<0.001**
Simien Mountains (S)	-0.35	-1.10	0.271	7.04	1.48	0.139	21.34	2.08	**0.038**
Sororo Torgam Gara Muktar (LS)	2.19	4.87	**<0.001**	-21.69	-1.67	0.095	8.43	0.72	0.470
Tama (LS)	0.16	1.00	0.316	-3.18	-5.38	**<0.001**	-5.74	-3.05	**0.002**
Telalak Dewe (LS)	-0.36	-3.48	**<0.001**	0.60	1.98	**0.048**	3.37	1.28	0.202
Tulu Lafto Sedden (LS)	-2.08	-2.33	**0.020**	-1.83	-1.90	0.057	-2.34	-2.01	**0.045**
Urgan Bula (LS)	0.21	0.50	0.617	-3.03	-1.60	0.109	-0.45	-0.03	0.976
Yangudi Rassa (S)	-0.03	-0.64	0.522	0.63	2.64	**0.008**	3.70	2.16	**0.031**
Yayu (LS)	2.03	8.56	**<0.001**	-2.46	-3.25	**0.001**	-1.94	-2.42	**0.016**

Supplementary Table 14 Individual protected area social wellbeing outputs showing the average treatment effect on the treated (ATT), t-statistic (t) and significance (p); significant p-values are shown in bold. A positive ATT indicates better performance.

Protected area	Months of adequate food			Dietary diversity			Material wellbeing		
	ATT	t	p	ATT	t	p	ATT	t	p
Abasheba Demero (LS)	-0.73	-2.36	**0.019**	0.23	0.34	0.733	-0.15	-0.14	0.893
Amibera Melika sadi (LS)	-1.61	-3.26	**0.001**	2.01	2.16	**0.031**	1.20	1.30	0.195
Asibahri Kebena (LS)	-8.30	-6.03	**<0.001**	1.13	1.63	0.104	0.35	0.49	0.625
Babile Elephant (LS)	-2.87	-3.05	**0.002**	-0.65	-1.00	0.320	-0.66	-2.25	**0.025**
Bale Mountains (S)	-2.29	-3.17	**0.002**	0.34	0.36	0.719	-1.10	-1.80	0.073
Billen Hertale (LS)	-5.75	-2.31	**0.021**	-0.18	-0.15	0.879	2.59	1.74	0.083
Borena (S)	1.26	2.36	**0.019**	2.66	4.08	**<0.001**	0.51	1.28	0.201
Borena sayint Worehimano (S)	0.04	0.07	0.943	-0.74	-0.76	0.448	-3.23	-3.71	**<0.001**
Chebera Churchura (S)	-0.81	-2.26	**0.024**	-1.90	-3.12	**0.002**	-12.75	-5.10	**<0.001**
Chifra (LS)	-2.83	-3.05	**0.002**	0.45	0.74	0.459	1.04	1.87	0.062
Dindin (LS)	-4.16	-3.05	**0.002**	-2.29	-1.71	0.089	1.67	1.36	0.173
Erer Gota (LS)	0.13	0.41	0.679	1.53	2.17	**0.030**	0.54	0.90	0.370
Gambella (S)	0.39	1.88	0.061	1.43	2.44	**0.015**	-2.43	-0.91	0.365
Gara Gumbi (LS)	-5.89	-7.86	**<0.001**	2.47	7.59	**<0.001**	0.11	0.33	0.745
Gara Meti (LS)	-2.77	-3.20	**0.001**	1.06	1.23	0.218	1.11	1.58	0.116
Gibe Sheleko (S)	-0.02	-0.04	0.968	2.10	3.37	**0.001**	0.59	0.77	0.440
Kafa (LS)	-0.68	-2.60	**0.010**	-0.20	-0.50	0.614	0.28	0.69	0.488
Kafta Sheraro (S)	0.80	1.10	0.272	-1.49	-1.74	0.083	2.02	2.51	**0.012**
Majang (LS)	0.14	0.53	0.593	1.25	1.52	0.129	1.78	3.53	**<0.001**
Melka Guba (S)	1.75	2.96	**0.003**	-0.62	-0.94	0.346	1.41	2.04	**0.042**
Munessa Kuke (LS)	0.56	0.97	0.331	-1.17	-1.77	0.077	-0.01	-0.02	0.986
Sheka (LS)	0.46	1.19	0.235	0.36	0.54	0.589	-0.30	-0.48	0.635
Shinele Meto (LS)	-6.56	-6.15	**<0.001**	2.21	3.34	**0.001**	1.78	2.44	**0.015**
Simien Mountains (S)	-0.83	-1.59	0.112	-2.36	-2.98	**0.003**	-0.80	-3.01	**0.003**
Sororo Torgam Gara Muktar (LS)	-1.28	-2.29	**0.023**	0.64	0.98	0.327	-1.63	-3.62	**<0.001**
Yayu (LS)	-0.62	-1.79	0.075	2.77	3.87	**<0.001**	-0.50	-1.88	0.061

Supplementary Table 15 Model averaged predictors of protected area environmental and wellbeing performance across models where ($\Delta AIC < 2$) demonstrate significant predictors for environmental performance are area-adjusted budget, precipitation and agricultural suitability, while for wellbeing performance none are significant.

Response	Predictor	Estimate (full)	Adjusted S.E	z-value	p-value
Environmental performance	Budget (area-adjusted)	0.54	0.14	3.78	<0.001
	Precipitation	-1.34	0.33	4.07	<0.001
	Agricultural suitability	-0.42	0.18	2.27	0.02
	Agricultural land	-0.08	0.15	0.53	0.60
	Strictness (strict)	0.14	0.33	0.41	0.68
Wellbeing performance	Agricultural suitability	0.65	0.57	1.39	0.17
	Access	-0.06	0.20	0.32	0.75
	Population	0.07	0.25	0.29	0.77
	Agricultural land	0.06	0.21	0.27	0.79

Supplementary Table 16 Changes from grassland to savanna and shrubland are associated with bush encroachment. Results from a linear regression with cattle, sheep and goat densities as predictors of cells which change from grassland to savanna or shrubland (Adjusted $R^2 = 0.006$, $F_{(3, 4836)} = 4.12$, $p = 0.006$)

Predictor	Estimate	S.E	t-value	p-value
Cattle density	7.65×10^{-6}	2.21×10^{-6}	3.47	<0.001
Sheep density	-6.41×10^{-6}	3.15×10^{-6}	-2.04	0.042
Goat density	-2.31×10^{-6}	2.38×10^{-6}	-0.97	0.333

Supplementary references

1. WorldPop. Global 1km Population Individual countries. University of Southampton https://doi.org/10.5258/SOTON/WP00670 (2020).
2. Potapov, P. *et al.* Global maps of cropland extent and change show accelerated cropland expansion in the twenty-first century. *Nat Food* **3**, 19–28 (2022).
3. Nelson, A. *Travel Time to Major Cities*. (European Comission, Italy, 2008).
4. EROS. Global 30 Arc-Second Elevation (GTOPO30). U.S. Geological Survey https://doi.org/10.5066/F7DF6PQS (2017).
5. Karger, D. N. *et al.* Climatologies at high resolution for the earth's land surface areas. *Sci Data* **4**, 170122 (2017).
6. BRAHMS. The Endemic Plants of Ethiopia BRAHMS Database. Version 7.9. Royal Botanic Gardens, Kew. Accessed on 06 September 2023. (2023).
7. Venter, Z. S., Cramer, M. D. & Hawkins, H.-J. Drivers of woody plant encroachment over Africa. *Nat Commun* **9**, 2272 (2018).
8. van Breugel, P., Friis, I., Demissew, S., Lillesø, J.-P. B. & Kindt, R. Current and Future Fire Regimes and Their Influence on Natural Vegetation in Ethiopia. *Ecosystems* **19**, 369–386 (2016).
9. Leta, S. & Mesele, F. Spatial analysis of cattle and shoat population in Ethiopia: growth trend, distribution and market access. *SpringerPlus* **3**, 310 (2014).
10. Gilbert, M. *et al.* Global cattle distribution in 2015 (5 minutes of arc). Harvard Dataverse https://doi.org/10.7910/DVN/LHBICE (2022).
11. Friedl, Mark & Sulla-Menashe, Damien. MCD12Q1 MODIS/Terra+Aqua Land Cover Type Yearly L3 Global 500m SIN Grid V006. NASA EOSDIS Land Processes DAAC https://doi.org/10.5067/MODIS/MCD12Q1.006 (2019).
12. Allaire, J. J. *et al.* networkD3: D3 JavaScript Network Graphs from R. (2017).
13. Venter, O. *et al.* Bias in protected-area location and its effects on long-term aspirations of biodiversity conventions. *Conservation Biology* **32**, 127–134 (2018).
14. O'Garra, T., Martin, R., Pynegar, E., Polo-Urrea, C. & Eklund, J. Selecting among counterfactual methods to evaluate conservation interventions. *Conservation Science and Practice* **7**, e70066 (2025).
15. Schleicher, J. *et al.* Statistical matching for conservation science. *Conservation Biology* **34**, 538–549 (2020).

16. Bastardoz, N. *et al.* Instrumental variables estimation: Assumptions, pitfalls, and guidelines. *The Leadership Quarterly* **34**, 101673 (2023).

17. Wuepper, D. & Finger, R. Regression discontinuity designs in agricultural and environmental economics. *Eur Rev Agric Econ* **50**, 1–28 (2023).

18. Fredriksson, A. & Oliveira, G. M. de. Impact evaluation using Difference-in-Differences. *RAUSP Management Journal* **54**, 519–532 (2019).

19. Geldmann, J., Jones, J. P. G., Wauchope, H. & Ferraro, P. J. Causal claims, causal assumptions and protected area impact. *Nature* **638**, E40–E41 (2025).

20. Cinelli, C. *et al.* sensemakr: Sensitivity Analysis Tools for Regression Models. (2024).

21. Beyene, A. D. *et al.* Contribution of non-timber forest products to the livelihood of farmers in coffee growing areas: evidence from Yayu Coffee Forest Biosphere Reserve. *Journal of Environmental Planning and Management* **63**, 1633–1654 (2020).

22. International Climate Initiative. Restoring degraded coffee landscapes in Ethiopia. https://www.international-climate-initiative.com/en/project/restoring-degraded-coffee-landscapes-in-ethiopia-18-iii-078-eth-a-restoring-coffee-landscapes/ (2018).

23. Wakjira, M. T., Peleg, N., Six, J. & Molnar, P. Current and future cropland suitability for cereal production across the rainfed agricultural landscapes of Ethiopia. *Agricultural and Forest Meteorology* **358**, 110262 (2024).

24. Kiros, S. & Bekele, A. Assessment of conservation challenges in and around Gibe Sheleko National Park, southwestern Ethiopia. *Global Ecology and Conservation* **32**, e01912 (2021).

25. Fischer, A., Yitbarek, T., Czajkowski, M., Tadie, D. & Hanley, N. Trophy hunters' willingness to pay for wildlife conservation and community benefits. *Conservation Biology* **29**, 1111–1121 (2015).

26. Lind, J., Sabates-Wheeler, R., Caravani, M., Kuol, L. B. D. & Nightingale, D. M. Newly evolving pastoral and post-pastoral rangelands of Eastern Africa. *Pastoralism* **10**, 24 (2020).

27. Bassi, M. & Tache, B. The community conserved landscape of the Borana Oromo, Ethiopia. *Management of Environmental Quality: An International Journal* **22**, 174–186 (2011).

28. Ethiopian Wildlife Conservation Authority. *Ethiopian Elephant Action Plant (2015-2025)*. https://ethiopias-elephants.com/wp-content/uploads/2020/10/20181003-FINAL-Ethiopian-Elephant-Action-Plan-IP-RMPB.pdf (2015).

29. Central Statistical Agency of Ethiopia & LSMS-ISA. Rural Socioeconomic Survey 2011-2012. World Bank, Development Data Group https://doi.org/10.48529/80XT-9M68 (2012).

30. Central Statistical Agency of Ethiopia & LSMS-ISA. Socioeconomic Survey 2015-2016, Wave 3. World Bank, Development Data Group https://doi.org/10.48529/AMPF-7988 (2016).

31. Earth Engine. Hansen Global Forest Change v1.9 (2000-2021) | Earth Engine Data Catalog. *Google Developers* https://developers.google.com/earth-engine/datasets/catalog/UMD_hansen_global_forest_change_2021_v1_9 (2022).

32. Hansen, M. C. *et al.* High-Resolution Global Maps of 21st-Century Forest Cover Change. *Science* **342**, 850–853 (2013).

33. Jones, A. D., Ngure, F. M., Pelto, G. & Young, S. L. What Are We Assessing When We Measure Food Security? A Compendium and Review of Current Metrics12. *Adv Nutr* **4**, 481–505 (2013).

34. Vyas, S. & Kumaranayake, L. Constructing socio-economic status indices: how to use principal components analysis. *Health Policy and Planning* **21**, 459–468 (2006).

35. Zabel, F. Global Agricultural Land Resources – A High Resolution Suitability Evaluation and Its Perspectives until 2100 under Climate Change Conditions (v3.0). Zenodo https://doi.org/10.5281/zenodo.5982577 (2022).

36. Dinerstein, E. *et al.* An Ecoregion-Based Approach to Protecting Half the Terrestrial Realm. *BioScience* **67**, 534–545 (2017).

37. Müller-Crepon, C. & Hunziker, P. New spatial data on ethnicity: Introducing SIDE. *Journal of Peace Research* **55**, 687–698 (2018).

38. Friedl, M. & Sulla-Menashe, D. MODIS/Terra+Aqua Land Cover Type Yearly L3 Global 500m SIN Grid V061. NASA EOSDIS Land Processes Distributed Active Archive Center https://doi.org/10.5067/MODIS/MCD12Q1.061 (2022).

39. Mouillot, D. *et al.* The socioeconomic and environmental niche of protected areas reveals global conservation gaps and opportunities. *Nat Commun* **15**, 9007 (2024).

40. Joppa, L. N. & Pfaff, A. High and Far: Biases in the Location of Protected Areas. *PLOS ONE* **4**, e8273 (2009).

41. Busch, J. & Ferretti-Gallon, K. What Drives Deforestation and What Stops It? A Meta-Analysis. *Review of Environmental Economics and Policy* **11**, 3–23 (2017).

42. Getahun, K., Van Rompaey, A., Van Turnhout, P. & Poesen, J. Factors controlling patterns of deforestation in moist evergreen Afromontane forests of Southwest Ethiopia. *Forest Ecology and Management* **304**, 171–181 (2013).
43. Birhanu, L., Hailu, B. T., Bekele, T. & Demissew, S. Land use/land cover change along elevation and slope gradient in highlands of Ethiopia. *Remote Sensing Applications: Society and Environment* **16**, 100260 (2019).
44. Stifel, D. & Minten, B. Market Access, Well-being, and Nutrition: Evidence from Ethiopia. *World Development* **90**, 229–241 (2017).
45. Samberg, L. H., Shennan, C. & Zavaleta, E. S. Human and Environmental Factors Affect Patterns of Crop Diversity in an Ethiopian Highland Agroecosystem. *The Professional Geographer* **62**, 395–408 (2010).
46. Moges, A. & Holden, N. M. Soil Fertility in Relation to Slope Position and Agricultural Land Use: A Case Study of Umbulo Catchment in Southern Ethiopia. *Environmental Management* **42**, 753–763 (2008).
47. Gou, Y. *et al.* Intra-annual relationship between precipitation and forest disturbance in the African rainforest. *Environ. Res. Lett.* **17**, 044044 (2022).
48. Neke, K. S. & Du Plessis, M. A. The Threat of Transformation: Quantifying the Vulnerability of Grasslands in South Africa. *Conservation Biology* **18**, 466–477 (2004).
49. Demeke, A. B., Keil, A. & Zeller, M. Using panel data to estimate the effect of rainfall shocks on smallholders food security and vulnerability in rural Ethiopia. *Climatic Change* **108**, 185–206 (2011).
50. Makate, C., Angelsen, A., Holden, S. T. & Westengen, O. T. Crops in crises: Shocks shape smallholders' diversification in rural Ethiopia. *World Development* **159**, 106054 (2022).
51. He, X. & Chen, Z. Weather, cropland expansion, and deforestation in Ethiopia. *Journal of Environmental Economics and Management* **111**, 102586 (2022).
52. Jellason, N. P. *et al.* A Systematic Review of Drivers and Constraints on Agricultural Expansion in Sub-Saharan Africa. *Land* **10**, 332 (2021).
53. Akpoti, K., Kabo-bah, A. T. & Zwart, S. J. Review - Agricultural land suitability analysis: State-of-the-art and outlooks for integration of climate change analysis. *Agricultural Systems* **173**, 172–208 (2019).
54. Terefe, A. T., Aredo, M. K., Workagegnehu, A. M. & Tesfaye, W. M. Interdependence of rural household welfare measurement in the context of climate variability in Ethiopia. *Heliyon* **10**, (2024).
55. *The Soils of Ethiopia*. (Springer International Publishing, Cham, 2023). doi:10.1007/978-3-031-17012-6.

56. Mekuria, W. & Mekonnen, K. Determinants of crop–livestock diversification in the mixed farming systems: evidence from central highlands of Ethiopia. *Agric & Food Secur* **7**, 60 (2018).

57. *The Soils of Ethiopia*. (Springer, Berlin, 2023).

58. Husmann, C. Marginality as a Root Cause of Poverty: Identifying Marginality Hotspots in Ethiopia. *World Development* **78**, 420–435 (2016).

59. Ethiopian Biodiversity Institute. *Ethiopia's Fifth National Report to the Convention on Biological Diversity*. https://www.ebi.gov.et/wp-content/uploads/2021/06/et-nr-05-en.pdf (2014).

60. Bullock, E. L. *et al.* Three Decades of Land Cover Change in East Africa. *Land* **10**, 150 (2021).

61. Gebre-Selassie, A. & Bekele, T. *A Review of Ethiopian Agriculture: Roles, Policy and Small-Scale Farming Systems*.

62. Chase, R. R. *et al.* Smallholder farmers expand production area of the perennial crop enset as a climate coping strategy in a drought-prone indigenous agrisystem. *PLANTS, PEOPLE, PLANET* **5**, 254–266 (2023).

63. Matewos, T. Climate Change-Induced Impacts on Smallholder Farmers in Selected Districts of Sidama, Southern Ethiopia. *Climate* **7**, 70 (2019).

64. Adimassu, Z., Kessler, A. & Stroosnijder, L. Farmers' strategies to perceived trends of rainfall and crop productivity in the Central Rift Valley of Ethiopia. *Environmental Development* **11**, 123–140 (2014).

65. Jateno, W., Alemu, B. A. & Shete, M. Household dietary diversity across regions in Ethiopia: Evidence from Ethiopian socio-economic survey data. *PLOS ONE* **18**, e0283496 (2023).

66. Michalopoulos, S. & Papaioannou, E. National Institutions and Subnational Development in Africa *. *The Quarterly Journal of Economics* **129**, 151–213 (2014).

67. Akinyemi, F. O. & Ifejika Speranza, C. Agricultural landscape change impact on the quality of land: An African continent-wide assessment in gained and displaced agricultural lands. *International Journal of Applied Earth Observation and Geoinformation* **106**, 102644 (2022).

68. Rampersad, C. *et al.* Indigenous crop diversity maintained despite the introduction of major global crops in an African centre of agrobiodiversity. *PLANTS, PEOPLE, PLANET* **5**, 985–996 (2023).

69. Ryan, S. J. *et al.* Population pressure and global markets drive a decade of forest cover change in Africa's Albertine Rift. *Applied Geography* **81**, 52–59 (2017).

70. Wassie, S. B. Natural resource degradation tendencies in Ethiopia: a review. *Environ Syst Res* **9**, 33 (2020).

71. Josephson, A. L., Ricker-Gilbert, J. & Florax, R. J. G. M. How does population density influence agricultural intensification and productivity? Evidence from Ethiopia. *Food Policy* **48**, 142–152 (2014).

72. Workicho, A. *et al.* Household dietary diversity and Animal Source Food consumption in Ethiopia: evidence from the 2011 Welfare Monitoring Survey. *BMC Public Health* **16**, 1192 (2016).

73. Bigsten, A., Kebede, B., Shimeles, A. & Taddesse, M. Growth and Poverty Reduction in Ethiopia: Evidence from Household Panel Surveys. *World Development* **31**, 87–106 (2003).

74. Bogaert, J. *et al.* Fragmentation of Forest Landscapes in Central Africa: Causes, Consequences and Management. in *Patterns and Processes in Forest Landscapes: Multiple Use and Sustainable Management* (eds. Lafortezza, R., Sanesi, G., Chen, J. & Crow, T. R.) 67–87 (Springer Netherlands, Dordrecht, 2008). doi:10.1007/978-1-4020-8504-8_5.

75. Gashaw, T., Tulu, T., Argaw, M. & Worqlul, A. W. Evaluation and prediction of land use/land cover changes in the Andassa watershed, Blue Nile Basin, Ethiopia. *Environ Syst Res* **6**, 17 (2017).

76. Guyalo, A. K., Alemu, E. A. & Degaga, D. T. Impact of large-scale agricultural investments on the food security status of local community in Gambella region, Ethiopia. *Agric & Food Secur* **11**, 43 (2022).

77. Shete, M. & Rutten, M. Impacts of large-scale farming on local communities' food security and income levels – Empirical evidence from Oromia Region, Ethiopia. *Land Use Policy* **47**, 282–292 (2015).

78. Ricciardi, V., Ramankutty, N., Mehrabi, Z., Jarvis, L. & Chookolingo, B. How much of the world's food do smallholders produce? *Global Food Security* **17**, 64–72 (2018).

79. Headey, D., Dereje, M. & Taffesse, A. S. Land constraints and agricultural intensification in Ethiopia: A village-level analysis of high-potential areas. *Food Policy* **48**, 129–141 (2014).
