# Supplementary material for: Trade-offs between nature and people in Ethiopia’s protected areas demonstrate challenges in translating global conservation targets into national realities
Source: Nat Ecol Evol. 2026 May 12;10(6):1057–70. doi: 10.1038/s41559-026-03047-9 (PMC13253355; doi:10.1038/s41559-026-03047-9)
Supplement: Supplementary file 2 — Reporting Summary [file 41559_2026_3047_MOESM2_ESM.pdf]

nature portfolio

Corresponding author(s): James Borrell
Last updated by author(s): Feb 27, 2026

Reporting Summary

Nature Portfolio wishes to improve the reproducibility of the work that we publish. This form provides structure for consistency and transparency in reporting. For further information on Nature Portfolio policies, see our Editorial Policies and the Editorial Policy Checklist.

Statistics

For all statistical analyses, confirm that the following items are present in the figure legend, table legend, main text, or Methods section.

n/a	Confirmed	
☐	☒	The exact sample size (*n*) for each experimental group/condition, given as a discrete number and unit of measurement
☐	☒	A statement on whether measurements were taken from distinct samples or whether the same sample was measured repeatedly
☐	☒	The statistical test(s) used AND whether they are one- or two-sided<br>*Only common tests should be described solely by name; describe more complex techniques in the Methods section.*
☐	☒	A description of all covariates tested
☐	☒	A description of any assumptions or corrections, such as tests of normality and adjustment for multiple comparisons
☐	☒	A full description of the statistical parameters including central tendency (e.g. means) or other basic estimates (e.g. regression coefficient) AND variation (e.g. standard deviation) or associated estimates of uncertainty (e.g. confidence intervals)
☐	☒	For null hypothesis testing, the test statistic (e.g. *F*, *t*, *r*) with confidence intervals, effect sizes, degrees of freedom and *P* value noted<br>*Give P values as exact values whenever suitable.*
☒	☐	For Bayesian analysis, information on the choice of priors and Markov chain Monte Carlo settings
☐	☒	For hierarchical and complex designs, identification of the appropriate level for tests and full reporting of outcomes
☐	☒	Estimates of effect sizes (e.g. Cohen's *d*, Pearson's *r*), indicating how they were calculated

Our web collection on statistics for biologists contains articles on many of the points above.

Software and code

Policy information about availability of computer code

Data collection	No software was used for data collection
Data analysis	All statistical analysis were conducted in R version 4.2.1. The R codes are deposited in a community repository

For manuscripts utilizing custom algorithms or software that are central to the research but not yet described in published literature, software must be made available to editors and reviewers. We strongly encourage code deposition in a community repository (e.g. GitHub). See the Nature Portfolio guidelines for submitting code & software for further information.

Data

Policy information about availability of data

All manuscripts must include a data availability statement. This statement should provide the following information, where applicable:

- Accession codes, unique identifiers, or web links for publicly available datasets
- A description of any restrictions on data availability
- For clinical datasets or third party data, please ensure that the statement adheres to our policy

Updated protected area shapefiles are available https://github.com/SCJago/protected_area_performance/tree/main/data. Protected area budget data is available in categorical format in Supplementary Table S9, continuous numerical budget data requests should be directed to the Ethiopian Wildlife Conservation Authority. Species' range data is available for download from the IUCN Red List, either via a manual search (https://www.iucnredlist.org/search) or through the spatial database (https://www.iucnredlist.org/resources/spatial-data-download). Point data for plant species without IUCN Red List ranges can also be obtained from the

same sources. Occurrence data for Ethiopia's endemic plant species is stored in the "Endemic Plants of Ethiopia" database on RBG Kew BRAHMS Online. BRAHMS data requests should be directed to the relevant contact listed on this webpage: https://brahmsonline.kew.org/kewbol/Websites. All open source datasets used are referenced in the main manuscript/supplementary information.

Research involving human participants, their data, or biological material

Policy information about studies with human participants or human data. See also policy information about sex, gender (identity/presentation), and sexual orientation and race, ethnicity and racism.

Reporting on sex and gender	For the stakeholder questionnaire, gender information as self-reported by participants, with 14% identifying as female and 86% identifying as male. These data were used to demonstrate the demographic diversity within the participant pool. Consent for sharing gender-related information was obtained as part of the broader informed consent process for the study.
Reporting on race, ethnicity, or other socially relevant groupings	Our quasi-experimental analysis used the first component of a principal component analysis of the proportion of the population identifying as each ethnolinguistic group as a covariate for statistical matching. Specific information on the data is available at New spatial data on ethnicity: Introducing SIDE | Carl Müller-Crepon
Population characteristics	Our quasi-experimental analysis uses data from 1km resolution population counts from 2000-2020 as a covariate for statistical matching. Specific information available: WorldPop :: Population Counts
Recruitment	Recruitment of stakeholders for the questionnaire is described in the main text. Participants were recruited using a purposive, opportunistic, snowball sampling approach. Participation was voluntary and informed consent was obtained from all respondents prior to data collection. Perceptions reflect reflect the demographic and professional composition of the sampled conservation practitioner community rather than the general population.
Ethics oversight	Approved by the University of Kent Conservation Ethics Committee: Ethics ID 20251741251220900

Note that full information on the approval of the study protocol must also be provided in the manuscript.

Field-specific reporting

Please select the one below that is the best fit for your research. If you are not sure, read the appropriate sections before making your selection.

☐ Life sciences ☐ Behavioural & social sciences ☒ Ecological, evolutionary & environmental sciences

For a reference copy of the document with all sections, see nature.com/documents/nr-reporting-summary-flat.pdf

Ecological, evolutionary & environmental sciences study design

All studies must disclose on these points even when the disclosure is negative.

Study description	We evaluated Ethiopia's protected area networks performance for meeting global biodiversity targets. We revised protected boundaries, assessed ecological representativeness across ecoregions and species, tested effectiveness for maintaining habitats and resisting anthropogenic pressures as well as food security and material wellbeing outcomes using quasi-experimental methods, and surveyed conservation stakeholders on priorities for the protected area network.
Research sample	Spatial: 79 gazetted PAs after revision of WDPA records with Ethiopian Wildlife Conservation Authority data.<br>Environmental outcomes: 1 km² grid cells (treatment = PA cells, control = unprotected cells).<br>Social outcomes: 3,699 households in the LSMS-ISA Ethiopian Socio-economic Survey panel (2011–2016).<br>Stakeholder survey: 37 Ethiopian conservation practitioners, researchers and policymakers.
Sampling strategy	Environment: Grid-based sampling with 2 km spacing to avoid autocorrelation. Treatment = protected area cells; controls = outside of a 10km buffer around each protected area.<br>Social: Households within 10 km buffers of protected areas = treatment; >20 km = control.<br>Stakeholders: Purposive snowball sampling of experts directly engaged in PA policy, research or management.
Data collection	Majority of the study used open source datasets downloaded online. For the questionnaire data was collected via email by Gebremeskel Gizaw (Ethiopian Wildlife Conservation Authority)
Timing and spatial scale	National coverage of Ethiopia.<br>Land-cover change: 2000–2020/21.<br>Household wellbeing: 2011–2016 panel.
Data exclusions	57 National Forest Priority Areas excluded (not protected areas). After updating protected area boundaries, 12 degazetted, 1 duplicate, 2 amalgamated protected areas were excluded. 16 PAs established after baseline excluded from effectiveness analyses (63 of 79 retained). Household records which were not resurveyed in 2016 were excluded. Unmatched grid cells or households excluded during statistical matching.
Reproducibility	All analyses conducted in R with reproducible scripts. Multiple matching methods tested (propensity score, Mahalanobis, calipers, replacement options). Robustness confirmed through >300 alternative model specifications and sensitivity tests (Sensemakr). Results

	were consistent across approaches.
Randomization	Treatment/control assignment defined by spatial rules (overlap with PA boundaries/buffers). Matching ensured baseline comparability.
Blinding	Not applicable to spatial and household datasets. Stakeholder survey responses anonymised prior to analysis.

Did the study involve field work? ☐ Yes ☒ No

Reporting for specific materials, systems and methods

We require information from authors about some types of materials, experimental systems and methods used in many studies. Here, indicate whether each material, system or method listed is relevant to your study. If you are not sure if a list item applies to your research, read the appropriate section before selecting a response.

Materials & experimental systems

n/a	Involved in the study
☒	☐ Antibodies
☒	☐ Eukaryotic cell lines
☒	☐ Palaeontology and archaeology
☒	☐ Animals and other organisms
☒	☐ Clinical data
☒	☐ Dual use research of concern
☒	☐ Plants

Methods

n/a	Involved in the study
☒	☐ ChIP-seq
☒	☐ Flow cytometry
☒	☐ MRI-based neuroimaging

Plants

Seed stocks	*Report on the source of all seed stocks or other plant material used. If applicable, state the seed stock centre and catalogue number. If plant specimens were collected from the field, describe the collection location, date and sampling procedures.*
Novel plant genotypes	*Describe the methods by which all novel plant genotypes were produced. This includes those generated by transgenic approaches, gene editing, chemical/radiation-based mutagenesis and hybridization. For transgenic lines, describe the transformation method, the number of independent lines analyzed and the generation upon which experiments were performed. For gene-edited lines, describe the editor used, the endogenous sequence targeted for editing, the targeting guide RNA sequence (if applicable) and how the editor was applied.*
Authentication	*Describe any authentication procedures for each seed stock used or novel genotype generated. Describe any experiments used to assess the effect of a mutation and, where applicable, how potential secondary effects (e.g. second site T-DNA insertions, mosiacism, off-target gene editing) were examined.*
