# Supplementary material for: Trade-offs between nature and people in Ethiopia’s protected areas demonstrate challenges in translating global conservation targets into national realities
Source: Nat Ecol Evol. 2026 May 12;10(6):1057–70. doi: 10.1038/s41559-026-03047-9 (PMC13253355; doi:10.1038/s41559-026-03047-9)
Supplement: Supplementary file 3 — Peer Review File [file 41559_2026_3047_MOESM3_ESM.pdf]

nature portfolio

Peer Review File

Trade-offs between nature and people reveal challenges in translating global conservation targets into national realities

Corresponding Author: Dr James Borrell

Version 0:

Decision Letter:

5th October 2025

*Please ensure you delete the link to your author homepage in this e-mail if you wish to forward it to your co-authors.

Dear Dr Borrell,

Your manuscript entitled "Trade-offs between nature and people reveal challenges in translating global conservation targets into national realities" has now been seen by three reviewers, whose comments are attached. The reviewers have raised a number of concerns which will need to be addressed before we can offer publication in Nature Ecology & Evolution. We will therefore need to see your responses to the criticisms raised and to some editorial concerns, along with a revised manuscript, before we can reach a final decision regarding publication.

We therefore invite you to revise your manuscript taking into account all reviewer and editor comments. Please highlight all changes in the manuscript text file.

We are committed to providing a fair and constructive peer-review process. Do not hesitate to contact us if there are specific requests from the reviewers that you believe are technically impossible or unlikely to yield a meaningful outcome.

When revising your manuscript:

* Include a “Response to reviewers” document detailing, point-by-point, how you addressed each reviewer comment. If no action was taken to address a point, you must provide a compelling argument. This response will be sent back to the reviewers along with the revised manuscript.

* If you have not done so already please begin to revise your manuscript so that it conforms to our Article format instructions at http://www.nature.com/natecolevol/info/final-submission. Refer also to any guidelines provided in this letter.

* Include a revised version of any required reporting checklist. It will be available to referees (and, potentially, statisticians) to aid in their evaluation if the manuscript goes back for peer review. A revised checklist is essential for re-review of the paper.

* Extended Data Figures - please ensure that any supplementary figures and tables that are crucial to the manuscript’s conclusions are converted into Extended Data figures and tables to increase visibility of these data. Extended Data figures and tables are online-only (present in the online PDF and full-text HTML versions of the paper), peer-reviewed display items that provide essential background to the article but are not included in the main article due to space constraints. A maximum of ten Extended Data display items (figures and tables) is permitted.

Please use the link below to submit your revised manuscript and related files:

Link Redacted

Note: This URL links to your confidential home page and associated information about manuscripts you may have submitted, or that you are reviewing for us. If you wish to forward this email to co-authors, please delete the link to your homepage.

We hope to receive your revised manuscript within four to eight weeks. If you cannot send it within this time, please let us know. We will be happy to consider your revision so long as nothing similar has been accepted for publication at Nature Ecology & Evolution or published elsewhere.

Nature Ecology & Evolution is committed to improving transparency in authorship. As part of our efforts in this direction, we are now requesting that all authors identified as ‘corresponding author’ on published papers create and link their Open Researcher and Contributor Identifier (ORCID) with their account on the Manuscript Tracking System (MTS), prior to acceptance. ORCID helps the scientific community achieve unambiguous attribution of all scholarly contributions. You can create and link your ORCID from the home page of the MTS by clicking on ‘Modify my Springer Nature account’. For more information please visit please visit www.springernature.com/orcid.

Please do not hesitate to contact me if you have any questions or would like to discuss these revisions further.

We look forward to seeing the revised manuscript and thank you for the opportunity to review your work.

[redacted]

Reviewer expertise:

Reviewer #1: PAs and local communities, livelihoods

Reviewer #2: PA effectiveness

Reviewer #3: conservation and development economics

Reviewer comments:

Reviewer #1 (Remarks to the Author):

This is an interesting, detailed and thorough paper. Although only a single-country study, it is convincing precisely because of the depth of analysis. It is an important contribution to this general area of research.

I have two comments which apply to the analysis. Firstly, would it be helpful to analyse and discuss the variation in performance by ecotype a bit more. I can imagine that livelihood strategies etc would vary significantly by ecosystems, and may explain a fair bit of the variation.

Secondly, it is disappointing to see the politics and social trends relegated to the supplementary information, given that we can assume that this is a major part of the trends observed. A simple before/after comparison works for the counterfactual, but it masks a complex history which might explain the variation in performance and the general trends observed. For example, how might the idea of parks as refugia have a legacy on impacts that go beyond the end of the Derg regime (as in, it reshaped livelihoods and population patterns that continued long after conflict). Or how things like subsequent conflict have reworked patterns of population and of livelihoods. Or the role of NGOs like African Parks in funding protected areas. So, in other words, adding some explanation of key political, social, economic trends as explanations of possible trends observed. Of course, it is up to the editors to decide whether or not the paper which is quite descriptive of trends rather than engaging in political explanations is better suited to the journal.

Reviewer #2 (Remarks to the Author):

This is a very timely paper exploring how well PAs in Ethiopia are performing both in terms of biodiversity coverage and in mitigating land-use change pressures, and also investigating if the PAs have adverse effects on local development and wellbeing. The paper thus provides a comprehensive view of how well the PA network has delivered for people and nature. This is especially timely in light of the new 30 by 30 target of the Global Biodiversity Framework of the CBD. I believe many in the conservation community have serious concerns about how such an ambitious scaling up of area-based conservation might play out in poor countries in the Global South. This study provides some answers and the results have high policy relevance. The findings and the paper are framed around the trade-off, even if I would have expected even more discouraging results, and some of the win-wins could be discussed: what lessons learned, why do these PAs function?

There have been some studies that have explored the same theme before:
https://www.sciencedirect.com/science/article/abs/pii/S0264837723000479
https://www.sciencedirect.com/science/article/pii/S0960982224006870

both missing from the ref list, so there is a need to convince the reader of why and how this study is novel/different and these papers also provide good food for thought for the discussion and how generalizable the findings might be beyond Ethiopia.

The figures are all very easy to understand and informative and summarizes the main findings in an elegant and insightful way.

My biggest concern is the main text. Considering the robustness of the analyses and the insightful ways of illustrating the findings, the main text feels under-developed and will need a substantial rewrite. Especially the Discussion needs more

attention. The discussion should engage more with previous studies that have also explore the trade-off between conservation and wellbeing and also literature on who bears the costs of conservation. A possibility is also to consider how spatial conservation prioritization tools (Marxan, Zonation) could be used in the future to maximize species representation while minimizing opportunity costs, when expanding the PA network.

line 67: or at least require more funding to compensate for adverse effects?
line 90: do you mean IUCN category II (i.e. not plural)?
line 92: you have to keep in mind that at this stage, the reader has not yet read the methods, so you need to clarify this discrepancy and where it comes from and what data you were using and their reliability.
line 106: same as before, the reader has no idea what “Ethiopia’s multidimensional environmental space” is?
about representativeness, I wonder if your dataset could be used to also explore temporal trends in how “high and far” the PAs are located? i.e. are more recently established PAs located in areas of higher pressures?
line 127: you have to keep in mind that the reader has not yet read the methods, so you need to specify what the strict and less strict PAs are somewhere here in the main text (perhaps in first section of Results?).
line 133: really odd opening of paragraph here, what does this refer to, what biodiversity outcomes? you just had a paragraph about pressures and land-use change in PAs, are you referring to this as biodiversity outcomes?
line 137: can you mention the covariates used for this matching here please
line 151: here you mention environmental outcomes, is this the same as the biodiversity outcomes? Define what you mean and be consistent throughout.
line 158: what are these six effectiveness measures, you need to explain this clearly earlier on when setting the scene, since methods comes only later in the Nature format.
line 159: worth here to be specific about the nature of the trade-off, i.e. in favor of environmental outcomes or wellbeing?
line 167: accounting for PA size, right? i.e. management costs per hectare? also think about economies of scales, see e.g. https://www.sciencedirect.com/science/article/pii/S0006320710004258
https://conbio.onlinelibrary.wiley.com/doi/full/10.1111/csp2.12697
line 176: out of what options? need to clarify briefly, as methods come only later.
line 217: see my earlier comment, you could have done an analysis if this was the case so far in Ethiopia?
line 230: could have been said before, in general the discussion is just a repetition of results and points raised earlier, little useful synthesis and a missed chance to enter a dialogue with previous literature.
line 283: I am missing a bit better referencing to previous literature looking into who bears the cost of conservation, e.g https://peerj.com/articles/5106/ but I think Neal Hockley has worked on this also more generally so worth to look into.
line 291: when gazetted?
line 294: how did you deal with the overlaps?
line 302: why is this more accurate?
line 310: what is this environmental space, please explain better
line 330: here comes the definitions I was missing, use this terminology consistently in the main text.
line 334: give more info on data for the outcomes and how it was done. otherwise not possible to evaluate how rigorous.
line 344: sure, but how about the time period? doesn’t match with your PA polygons? Also the sample seems small, could you put it on a map in suppl so the reader could see the spatial extent and possible spatial aggregation?
line 347: did you mask them out from serving as control sites for the matching?
line 370: what period of instability, you need to state this specifically, ideally already in the main text
line 392: please specify what you mean with random offset here, not just in SI.
line 476: needs to be budget per hectare, not overall budget, see previous comment and references provided.

Reviewer #3 (Remarks to the Author):

This manuscript assesses the representativeness of the protected areas network in Ethiopia, and attempts to quantify the impact of these protected areas on land cover and human wellbeing (food intake and assets)
It is well-written, and the results are interesting. A lot of work has been put into this work. However, while the results would be of great interest to a field journal, I believe the contribution is not substantial enough for Nature Ecology & Evolution.
Among all policy instruments, protected areas have been the most widely studied in the academic literature. Their (partial) ability to limit the expansion of the human footprint has been widely documented. The use of socio-economic surveys is less common, but the question of the possible negative effect on neighboring communities has also been quantified in several studies (e.g., Naidoo et al. 2019). The existing literature could be largely refined, notably by adopting more robust methodologies. In this manuscript, however, the authors mostly replicate the existing methodology without advancing it. Hence, the paper does not bring much thematic or methodological contribution.
In particular, the authors use statistical matching to correct for the possible non-random location of protected areas. While doing so is better than performing naïve comparisons between treated and control observations, matching has also been shown to be an extremely flawed method. While the authors demonstrate that the direction of the effect holds for many matching procedures, their figures also indicate that the point estimate varies significantly depending on the choice of model.
As the extent of protected areas has increased since the 2000s, why haven’t we focused on these protected areas and estimated a Difference in Difference / BACI model? This would help you relax several hypotheses to claim causality. (I understand that it would have been more complicated for the socio-economic outcomes with only data points at two dates.
A handful of more precise comments regarding the use of LSMS data, which might be the most orginal contribution of this paper:
« Measuring change using the panel data ensures any patterns we see are not due to people immigrating or emigrating from the area”. I believe this claim is not correct. Yoi have attrition in your data, and this attrition can be different across treatments.

- From your methodology, you don’t seem to have used the sampling weights of LSMS, which are necessary to produce unbiased results.
- You mention that you detail your LSMS outcomes in Table 1 but I can’t find the table in the manuscript.

For both LSMS and ecological data, you could consider clustering your standard errors at a higher level than the matched pairs. This could be the enumeration area for LSMS data or some administrative boundaries.

********************END********************

Version 1:

Decision Letter:

15th February 2026

Dear James,

Thank you for submitting your revised manuscript "Trade-offs between nature and people reveal challenges in translating global conservation targets into national realities" (NATECOLEVOL-25093024A), and thank you also for your patience while we gathered further reviews.

The revised paper has now been seen again by the original three reviewers, and also an additional Reviewer 4, who has expertise in Ethiopian conservation and wildlife science. The four reviewers' comments are below. The reviewers find that the paper has improved in revision, and therefore we'll be happy in principle to publish it in Nature Ecology & Evolution, pending minor revisions to satisfy the reviewers' final requests and to comply with our editorial and formatting guidelines.

As you will see from the comments below, Reviewer 3 has substantial remaining technical concerns. We asked Reviewer 2 to provide some further comment on these concerns. I have pasted those comments below, with Reviewer 2's permission, and will also share them with Reviewer 3. We feel that these comments raise very interesting points that we would like to see further discussion of in your revised article, but that they should not preclude publication of your paper. As you finalise your paper, please do include some additional discussion around these points, and please feel free to email me if you have any questions around this.

At this stage, please do not upload a revised article until you receive a pre-publication checklist from us. If the current version of your manuscript is in a PDF format, please email us a copy of the file in an editable format (Microsoft Word or LaTex)-- we can not proceed with PDFs at this stage.

If you have not done so already, please ensure that you also email us a completed copy of the Reporting summary :

Reporting summary: https://www.nature.com/documents/nr-reporting-summary.pdf

We are now performing detailed checks on your paper and will send you a checklist detailing our editorial and formatting requirements in about a week. Please do not upload the final materials and make any revisions until you receive this additional information from us.

Thank you again for your interest in Nature Ecology & Evolution. Please do not hesitate to contact me if you have any questions.

[redacted]

Reviewer #1 (Remarks to the Author):

N/A the authors have answered my queries. I remain mildly concerned about the lack of political differentiation, but recognise the complexities of doing this in a country that is so ecologically and socially varied.

Reviewer #2 (Remarks to the Author):

I find this to be a very carefully designed study with robust analytical choices. It is rare to see e.g. the sensitivity testing of arbitrary matching choices at the scale done here. I also find the replies to the previous round of reviewer comments convincing. The authors have embarked on a number of new analyses based on the comments by reviewers and they have fine-tuned their analyses and incorporated for example a consideration of funding/area and possible economies of scales of PA management costs. One of my previous concerns was the limited household data, but the authors show that the household data they have used is still a larger sample than similar data used in other studies. As far as I can see, this is a study that builds on the best data available and using state of the art matching to analyze it in a robust way.

The paper is now also more carefully framed and nuanced in its discussion of the findings and previous literature. The scope of this study is both ambitious and novel as it inspects PA performance from so diverse perspectives, most studies so far have focused on estimating either coverage/PA effectiveness in mitigating land-use change/ or PA impacts on local development. This study does all three and even adds stakeholder views, all within a carefully crafted study of a single case country which allows depth and use of the best available national data.

In conclusion, I can see that the authors have done a lot of hard work in addressing previous concerns by us reviewers and I am happy with this new version. I find the results extremely interesting and believe this will be a valuable and important contribution to the debate around the implementation of the 30by30 target.

Reviewer #3 (Remarks to the Author):

I thank the authors for their response to my comments. While the topic remains important, I maintain that the methodological limitations prevent the paper from producing trustworthy causal estimates – or at least with the level of certainty that the authors claim. Below I detail my remaining concerns. Unless the authors implement an alternative methodology to test for the internal validity of their results, I would recommend to reject the paper. Given the data available to the authors and the context, a difference-in-differences model or a difference-in-discontinuity model seems appropriate.

Main methodological concern: identification strategy
My central concern remains the choice of statistical matching as the identification strategy. The authors state in their answers that they "fully agree [that matching] has limitations," yet they defend their approach without seriously exploring alternatives or reflecting these limitations in the main manuscript.
The authors rule out other approaches by assertion rather than demonstration. Other studies have successfully used instrumental variable approaches in similar contexts (e.g., Sims 2010; Libois et al. 2022). Here the authors simply state they cannot do so without explanation. Likewise for regression discontinuity design: the authors claim that conditions differ just inside and outside protected area boundaries, but do not show this. If the authors wish to convincingly rule out RDD, they should provide standard validation graphs demonstrating discontinuities in covariates at the boundary—this would support their claim that the approach is infeasible.

When defending matching over BACI / Difference in differences, the authors fail to recognize the fundamental trade-off between internal and external validity. Matching relies on the assumption that selection into treatment is fully captured by observable covariates—an assumption difficult to defend in the context of PA placement, where unobserved political, ecological, and accessibility factors likely drive both placement and outcomes. Other methods, such as BACI allow to drastically limit the number of assumptions to made. It comes at a cost: focusing on protected areas created over the pat 20 years. The authors choose a method with questionable internal validity in order to generalize results. But generalizing results from a method that lacks internal validity simply means generalizing untrustworthy estimates.

Interpretation of impact evaluation results
Despite acknowledging matching's limitations in their response, the authors' results still read as definitive causal claims. For example, they state that "Protected areas *reduced* forest cover loss by *25%* compared to control." This language implies far more certainty than the methodology can support in terms of causality and the precision of estimates - particularly given that point estimates in Figure 3 vary by a factor of three across specifications. This concern is compounded by the authors' tendency to present robustness tests selectively: claiming that 97% of specifications yield results in the same direction, while failing to acknowledge the substantial variation in point estimates—variation that should temper confidence in any single estimate. The same critic applies across the entire section.

The section on trade-offs between environmental and social outcomes assumes that LSMS data are representative of wellbeing at the PA level. However, LSMS surveys are designed to be nationally representative, not locally representative of PA-adjacent populations. Yet, you are computing an average treatment effect by Protected Area to make this analysis hence assuming that the households in LSMS are representative of the population of this specific area.
Additionally, the analysis explaining heterogeneity in PA-level effects uses estimated ATTs as the dependent variable. Because these ATTs are estimated with considerable noise (as Figure 3 demonstrates), using point estimates without accounting for their uncertainty is problematic. This is an advanced generated regressors problem: errors propagate from the first to the second stage ; and the error in the first model is quite large according to Figure 3. The current approach does not account for this, yielding incorrect standard errors and probably effect sizes.

Missing studies in R3.01
“1. Environmental and wellbeing outcomes are rarely assessed together”
Some key missing studies in your answer attenuate the novelty of your claim:
Sims, K. R. (2010). Conservation and development: Evidence from Thai protected areas. Journal of environmental economics and management, 60(2), 94-114.

Libois, F., Baland, J. M., Delbart, N., & Pattanayak, S. (2022). Community Forest Management: The story behind a success story in Nepal.

Alix-Garcia, J. M., Sims, K. R., & Yañez-Pagans, P. (2015). Only one tree from each seed? Environmental effectiveness and

poverty alleviation in Mexico’s payments for ecosystem services program. American Economic Journal: Economic Policy, 7(4), 1-40.

Sims, K. R., & Alix-Garcia, J. M. (2017). Parks versus PES: Evaluating direct and incentive-based land conservation in Mexico. Journal of Environmental Economics and Management, 86, 8-28.

« 2. Social outcomes are rarely assessed at the household level”
I don’t find this statement and answer completely convincing as your answer miss several relevant studies (well-pubished & well-cited , not obscure papers). This includes:
Naughton-Treves, L., Alix-Garcia, J., & Chapman, C. A. (2011). Lessons about parks and poverty from a decade of forest loss and economic growth around Kibale National Park, Uganda. Proceedings of the National Academy of Sciences, 108(34), 13919-13924.

Nowakowski, A. J., Canty, S. W., Bennett, N. J., Cox, C. E., Valdivia, A., Deichmann, J. L., ... & McField, M. (2023). Co-benefits of marine protected areas for nature and people. Nature Sustainability, 6(10), 1210-1218.

Desbureaux, S., Girard, J., Dalongeville, A., Devillers, R., Mouillot, D., Jiddawi, N., ... & Leblois, A. (2024). The long-term impacts of Marine Protected Areas on fish catch and socioeconomic development in Tanzania. Conservation Letters, 17(6), e13048.

Reviewer #4 (Remarks to the Author):

Title: Trade-offs between nature and people reveal challenges in translating global conservation targets into national realities

I enjoyed reading the manuscript, which aims to evaluate whether Ethiopia could expand its protected areas, which currently cover about 10% of the country, to achieve the Global Biodiversity Framework’s 30-by-30 target, excluding Forest Priority Areas. Generally, the manuscript is well designed and well written, covering large topics. It explores the conservation challenges facing developing countries, particularly those affecting local livelihoods and protected areas. This will help design policy and management strategies for the short and long term. I recommend the manuscript as a minor review.

I have a few minor and major comments below to help improve the quality of the manuscript.
Abstract:
Line 43: Does it also help species conservation or….

Introduction
The Introduction is well framed and written from general to specific, with clear background information.
Line 79: … does the only concern is …. “local food supplies”…
Probably a local livelihood in which local communities not only depend on agriculture but also on cattle grazing, the use of forest resources, etc

Results
Line 150-158: Good to clarify more the number of threatened species in each group compared to the total species number in each category, such as mammals, birds, amphibians, reptiles and plants. Fig. 2b does not clearly show the details as it only shows the proportions. How about the endemic animal species representation in protected areas? Good to mention in the text.

Line 186-189: See the comment in the abstract above. The household income of local people living in the vicinity of protected areas is affected. Their livelihoods depend on agriculture, domestic animal grazing, and firewood collection, but they lack access to these resources in strictly protected areas. The competition for resources beyond food.

Discussion
Line 289-281: How about the long-term solution of expanding protected areas? In the future, industrial transformation, such as that in China, will likely increase urbanization and reduce the number of people living near protected areas, which leaves more land for protected area expansion. However, such a transformation would likely be supported by developed nations as part of climate change adaptation and capacity building. Good to think about short-term and long-term solutions.
It would be great to summarize the main findings of your study in one concluding paragraph or two, including implications for conservation, policy and local livelihood development for the short-term and long-term.

Materials and Methods:
The Materials and Methods section is detailed, which is helpful for researchers who could carry out similar studies. However, it is long in its current form and not suitable for general readers. I suggest the authors should reduce it by about 1/4th. It is not necessary to explain the details, and it would be good to move part of it to the supplementary file.

Line 386-388: Please double-check the number of amphibian and reptile species found in Ethiopia. The number of amphibian and reptile species appears lower than that found in Ethiopia. Please double-check recent studies. It would be great to mention the number of threatened animal and plant species in the text.

Line 896: Double check whether it is … 200 or ….2000 …

***

Additional comments from Reviewer 2 in response to Reviewer 3's comments:

Regarding the reviewer's comments, I believe the suggestions are technically very interesting but, in this particular case, perhaps a bit unreasonable. In an ideal world with unlimited data, a difference-in-differences (DiD) approach would indeed be preferable to simple matching. The before-after component would increase internal validity by strengthening the evidence that observed differences in outcomes are due to the intervention rather than pre-existing differences. However, the challenge here, as in most protected area (PA) studies, is that the majority of PAs were established long before remote sensing data on forest loss became available. This means that a DiD design would be limited to PAs established after 2000. Based on my reading of the Ethiopia case, such an analysis could only be conducted for 16 out of the 79 PAs, severely limiting the scope of the study and its policy relevance. Rather than strictly requesting this addition or completely reframing the paper, I feel this is an interesting research question that would merit a separate study.

As for the suggestion to use a regression discontinuity design (RDD), I am somewhat confused. My understanding is that RDD is most useful when an eligibility criterion can serve as a proxy or cut-off point for who or what receives an intervention. The reviewer writes that "the authors claim that conditions differ just inside and outside protected area boundaries, but do not show this." However, I fail to see why such an approach would be necessary here, as the PA polygons themselves are the intervention. Hence, since spatial data on the intervention is available, why revert to a proxy when the actual delineation is already known?

The instrumental variable approach is an interesting idea, but it is rarely used in PA effectiveness studies. I would be keen to hear what instrumental variable the reviewer suggests that could work in the Ethiopian context. The reviewer refers to studies in Thailand that used priority watershed status and another study on community forest management in Nepal (though I could only find working paper versions of the latter, so just wanted to flag that it may not have undergone peer review). Again, I find myself asking why such an approach would be preferable to using the spatially delineated PA boundaries, which, by definition, represent the intervention.

In sum, I believe that a study design based on matching is the best option given the data limitations (i.e., forest loss satellite data being available only from 2000 onwards). While a DiD design would be ideal, it would severely restrict the scope to only the 16 most recently established PAs out of 79. I therefore find it unreasonable to demand a different methodology and reject the paper based on this. Instead, in my opinion, it is enough that the authors stress that matching is sensitive to unobserved confounders. Note also that the authors have done some proper sensitivity analyses and show how much an unobserved variable would need to explain to be relevant. I think the following two papers can be used justify why matching in this case is ok (albeit not ideal):

- O'Garra, T., Mangubhai, S., Jagadish, A., Tabunakawai-Vakalalabure, M., Tawake, A., Govan, H., & Mills, M. (2023). National-level evaluation of a community-based marine management initiative. Nature Sustainability, 6, 908–918.
- O'Garra (2025). Selecting among counterfactual methods to evaluate conservation interventions. Conservation Science and Practice, 7, e70066.

Open Access This Peer Review File is licensed under a Creative Commons Attribution 4.0 International License, which

permits use, sharing, adaptation, distribution and reproduction in any medium or format, as long as you give appropriate credit to the original author(s) and the source, provide a link to the Creative Commons license, and indicate if changes were made.
In cases where reviewers are anonymous, credit should be given to 'Anonymous Referee' and the source.
The images or other third party material in this Peer Review File are included in the article’s Creative Commons license, unless indicated otherwise in a credit line to the material. If material is not included in the article’s Creative Commons license and your intended use is not permitted by statutory regulation or exceeds the permitted use, you will need to obtain permission directly from the copyright holder.
To view a copy of this license, visit https://creativecommons.org/licenses/by/4.0/

Reviewer comments:

Reviewer #1 (Remarks to the Author):

R1.01 This is an interesting, detailed and thorough paper. Although only a single-country study, it is convincing precisely because of the depth of analysis. It is an important contribution to this general area of research.

We thank the reviewer for these positive comments and agree that single-country studies enable a much more comprehensive, accurate and context relevant analysis. With both Ethiopian and international members of our authorship team having visited many of these sites, we have much greater confidence in the data and analysis than would be feasible for a global or regional study.

R1.02 I have two comments which apply to the analysis. Firstly, would it be helpful to analyse and discuss the variation in performance by ecotype a bit more. I can imagine that livelihood strategies etc would vary significantly by ecosystems, and may explain a fair bit of the variation.

We thank the reviewer for this helpful suggestion; we agree that providing more context for variation in performance is important. In response **we have updated our original analysis comparing performance between protected areas to now assess association between multiple potential predictors of protected area performance** (rather than just budget, as presented previously). We agree that livelihood strategies in Ethiopia are influenced by climate and also by other accessibility factors, and while we do not have reliable data on livelihood strategies across Ethiopia, we do have data on variables that may influence this and include these as proxies in the analysis.

Additions to the model

For our model comparing performance between protected areas, we now include additional potential predictors: temperature, precipitation, elevation, agricultural suitability, agricultural land cover, accessibility, management strictness and NGO involvement. We standardised continuous variables; excluded variables with variance inflation factors > 5; compared fixed- vs random-effects base models using AIC and likelihood-ratio tests; then conducted AIC-ranked model selection and averaging (using the R package *MuMIn*), checking residual normality and homoscedasticity of the top performing models.

Justification of approach

We elected not to compare performance directly by ecoregion as sample sizes were highly unbalanced (ranging from 0 to 21 protected areas per ecoregion) and would therefore produce unstable estimates for poorly represented categories. Secondly, because "ecoregion" can include very different social contexts, treating it as a fixed effect would risk confounding and imprecise estimates for sparsely represented categories and make interpretation difficult. Therefore, instead we retained ecoregion as context by including it as a random intercept in mixed-effects models, thereby controlling baseline differences among ecoregions while estimating the effects of other predictors.

Unchanged headline results

Following this new analysis, our main finding (that budget is a predictor of environmental performance but not of wellbeing performance) was unchanged. However, the expanded model provides **new interesting insights**. We found that better environmental performance is best explained by higher area-adjusted budgets, lower mean annual precipitation and lower agricultural suitability, which we interpret as protected areas do better, from a conservation perspective, where opportunity costs for agricultural returns are lower.

Manuscript updates

1. We have revised the methods to explain the expanded modelling approach and the rationale for treating ecoregion as a random intercept:

   "We assessed predictors of protected area performance using data on main ecoregions, management strictness, non-governmental organisation involvement (Supplementary Table S8) as well as average temperature, precipitation, elevation, agricultural suitability, agricultural land cover, accessibility (data sources in Supplementary Table S5) and area-adjusted budgets. Protected area budget data (Figure 4B) was obtained from the Ethiopian Wildlife Conservation Authority as average annual budgets (in USD adjusted to 2014 inflation levels). To account for non-linear scaling of costs across protected area sizes (Armsworth et al., 2011; Le Bouille et al., 2022), we modelled budget as a function of area and used the residuals as an area-adjusted measure of financial input. Continuous predictors were standardised, and variables with high multicollinearity (variance inflation factor > 5) were excluded. We then modelled both biodiversity and wellbeing performance outcomes separately to maintain as much data as possible (as fewer protected areas were assessed for wellbeing outcomes, due to not all protected areas having households surveyed in the Living Standards Measurement Survey). For each outcome, we fitted linear mixed-effects models with ecoregion as a random intercept and compared them to fixed-effects models using Akaike Information Criterion (AIC) and likelihood ratio tests. Using the better fitted base model, we then performed model selection and averaging with the MuMIn package, ranking models by AIC and averaging across all models with AIC < 2. Residuals were examined for normality and homoscedasticity."

2. Updated the results to include the additional significant predictors:

   "Comparing between individual protected areas, full model-averaged estimates (Supplementary Table S15) indicate improved environmental performance was best explained by higher area-adjusted budgets ($\beta = 0.54$, $z = 3.78$, $p < 0.001$), less precipitation ($\beta = -1.33$, $z = 4.07$, $p < 0.001$) and less agricultural suitability ($\beta = -0.42$, $z = 2.27$, $p = 0.02$). For social wellbeing performance, full model averaged estimates (Supplementary Table S15) showed no significant predictors, the best-supported model included only agricultural suitability and here higher suitability was weakly associated with greater improvements in wellbeing ($\beta = 0.68$, $t = 2.06$, $p = 0.051$; R2 = 0.15)."

3. Added a supplementary table with full model averaged results (Supplementary Table S15):

Table S15 Model averaged predictors of protected area environmental and wellbeing performance across models where ($\Delta AIC < 2$) demonstrate significant predictors for environmental performance are area-adjusted budget, precipitation and agricultural suitability, while for wellbeing performance none are significant.

Response	Predictor	Estimate (full)	Adjusted S.E	z-value	p-value
Environmental performance	Budget (area-adjusted)	0.54	0.14	3.78	<0.001
	Precipitation	-1.34	0.33	4.07	<0.001
	Agricultural suitability	-0.42	0.18	2.27	0.02
	Agricultural land	-0.08	0.15	0.53	0.60
	Strictness (strict)	0.14	0.33	0.41	0.68
Wellbeing performance	Agricultural suitability	0.65	0.57	1.39	0.17
	Access	-0.06	0.20	0.32	0.75
	Population	0.07	0.25	0.29	0.77
	Agricultural land	0.06	0.21	0.27	0.79

4. Updated the discussion to add further interpretation of the variation in performance:

   - *"we find that environmental outcomes of protected areas are worse in wetter and more agriculturally suitable areas where agricultural expansion and timber extraction likely produce better returns (Lomax et al., 2024) while wellbeing outcomes are marginally better where agricultural suitability is greater. Therefore, future progress towards a more representative network will require careful spatial planning, using multi-objective spatial conservation prioritization tools to identify locations that help to deliver conservation goals at least cost to people (Arneth et al., 2023; Neubert et al., 2025)."*

   - *While we find that higher area-adjusted budgets are associated with improved environmental outcomes, they show no detectable relationship with wellbeing performance. This is consistent with evidence that protected area funding and management capacity (e.g., enforcement and habitat management) underpin ecological effectiveness (Coad et al., 2019), but that insufficient resources are being allocated to strategies that support livelihood improvements for surrounding communities (Admasu et al., 2020; Oldekop et al., 2016; Rakotonarivo et al., 2025)."*

   - *"Only a small subset of Ethiopian protected areas delivered 'win-win' outcomes (Fig. 4), often in places where local livelihoods were compatible with conservation (Supplementary Text S1)."*

5. Added supplementary text regarding win-win sites:

   "Text S1 Context for win-win sites

   We identify five protected areas experiencing win-win outcomes: Yayu Biosphere Reserve, Gibe Sheleko National Park, Erer-Gota Controlled Hunting Area, Borena National Park, and Kafta Sheraro National Park. Below we summarise plausible descriptive reasons consistent with these results.

   Yayu Biosphere Reserve: Households in Yayu obtain substantial income for forest coffee and other non-timber forest products (Beyene et al., 2020). This aligns both livelihood and conservation incentives for maintaining forest. Additionally it has a high budget (Supplementary Table S9) and associations with the International Climate Initiative with a project focused on restoring degraded coffee landscapes in Ethiopia (International Climate Initiative, 2018)

   Gibe Sheleko National Park: Gibe Sheleko is located in an area of high agricultural suitability (Wakjira et al., 2024) which may help communities to maintain food security with less expansion of agricultural land. Local livelihoods include honey bee rearing and eucalyptus plantations, and non-timber forest products and are recognised by local communities as a benefit from the park, improving their perceptions of it (Kiros & Bekele, 2021).

   Erer-Gota Controlled Hunting Area: Ethiopia's controlled hunting area framework allows community revenue shares, and contributes to the rural economies neighbouring the parks (Fischer et al., 2015) It is located in a hot arid region of Ethiopia that more suited to pastoralist lifestyles reducing pressure of agricultural conversion (Lind et al., 2020).

   Borena National Park: Borena is also located in an arid region dominated by pastoralist livelihoods (Lind et al., 2020). In Borana zone, Gadaa based institutions

> *historically organised pasture use and participatory rangeland management is prioritised (Bassi & Tache, 2011). It also has a comparatively high budget (Supplementary table S9).*
>
> *Kafta Sheraro National Park: Kafta Sheraro also has a high budget (Supplementary table S9) which likely supports better environmental outcomes and it is has a flagship transboundary elephant population making it a national priority and attracting external support (Ethiopian Wildlife Conservation Authority, 2015)."*

R1.03 Secondly, it is disappointing to see the politics and social trends relegated to the supplementary information, given that we can assume that this is a major part of the trends observed. A simple before/after comparison works for the counterfactual, but it masks a complex history which might explain the variation in performance and the general trends observed. For example, how might the idea of parks as refugia have a legacy on impacts that go beyond the end of the Derg regime (as in, it reshaped livelihoods and population patterns that continued long after conflict). Or how things like subsequent conflict have reworked patterns of population and of livelihoods. Or the role of NGOs like African Parks in funding protected areas. So, in other words, adding some explanation of key political, social, economic trends as explanations of possible trends observed. Of course, it is up to the editors to decide whether or not the paper which is quite descriptive of trends rather than engaging in political explanations is better suited to the journal.

We agree that factors such as the legacy of parks as refugia, post-conflict population patterns, and the role of NGOs shape today's landscapes and institutions, and have updated the discussion to reflect this. In the original manuscript, some of this important context (the history of conservation in Ethiopia alongside relevant political developments) is presented as a timeline figure in the supplementary information. We agree it would be good to move this figure to the main text but this would be more figures than allowed by the journal. If the editor allows it, we would be happy to move this figure.

Initially, we have made a clearer link to this figure in the results:

> *"Ethiopia's protected area network has expanded steadily over time (Figure 1B) <u>amid changing political regimes and evolving conservation policy (Supplementary Figure S2)</u>"*

We have also revised the discussion text to explicitly reference these historical and socio-political factors, ensuring that the broader context is integrated into the interpretation of our findings while maintaining the focus on the quasi-experimental analysis of protected area impacts. **Please see manuscript updates response to comment R1.02.**

Regarding the role of NGOs specifically, we agree that NGOs and donor projects can influence protected area outcomes.

- We have now collated information, where it is available and accessible, on each protected area and whether it has been associated with an NGO during our study period. We provide this is Supplementary Table S8.

- We have also included NGO presence as a variable in our updated models (**see response to previous comment R1.02**). This does not come up as a significant predictor of protected area performance in either model.

- Given that area-adjusted protected area budgets were not a significant predictor of wellbeing performance we also highlight in the discussion:

> *"Avoiding negative social impacts of protected areas will require additional, targeted social investments that go beyond core protected area budgets (Hockley et al., 2018)."*

We would also like to take this opportunity to highlight that our quasi-experimental design was carefully thought out to analyse post-2000 protected are performance – a phase of comparatively greater stability – for three main reasons:

1. Policy relevance:

   Ethiopia's policy decisions including conservation choices and budgeting are made under current governance and socio-political context. Estimating effects across earlier instability and very different institutional regimes would produce results that are less relevant and actionable for current policy.

2. External applicability

   By focusing the analysis on the post-2000 period and aligning covariates with present-day drivers, our findings translate more readily to other countries facing similar modern pressures and policy frameworks.

3. Credible causal claims

   Extending our analysis into earlier decades, which are influenced by very specific conflict and instability, we would introduce unobserved, time-varying confounding that our data cannot adequately address; any pooled estimates would risk being descriptive correlations rather than defensible causal effects, undermining the reliability of guidance for policy.

We do not dispute the importance of longer-run legacies; we interpret post-2000 outcomes as operating **within** those complexities. To address them within our scope, we condition on time-invariant geography and 2000-baseline covariates (as set out in our DAGs) so that pre-2000 patterns reflected in settlement, access, and land cover are taken into account to the extent they persist. In short, our aim is to explain **current** protected-area performance for **current** decisions in Ethiopia in a way that is also **transferrable** to other countries working toward Global Biodiversity Framework targets under comparable present-day constraints. We've updated the text to reflect this:

> In the methods: *"Focusing the analysis on the post-2000 period, aligns it with the policy context in which decisions are currently made."*
>
> In the discussion: *"Using this baseline allows us to rigorously evaluate contemporary protected area performance aligning the analysis with the governance, budgeting and reporting context under which conservation decisions are currently made, rather than conflating our analysis when conservation operated under a very different political context."*

<u>Reviewer #2 (Remarks to the Author):</u>

R2.01 This is a very timely paper exploring how well PAs in Ethiopia are performing both in terms of biodiversity coverage and in mitigating land-use change pressures, and also investigating if the PAs have adverse effects on local development and wellbeing. The paper thus provides a comprehensive view of how well the PA network has delivered for people and nature. This is especially timely in light of the new 30 by 30 target of the Global Biodiversity Framework of the CBD. I believe many in the conservation community have serious concerns about how such an ambitious scaling up of area-based conservation might play out in poor countries in the Global South. This study provides some answers and the results have high policy relevance. The findings and the paper are framed around the trade-off, even if I would have expected even more discouraging results, and some of the win-wins could be discussed: what lessons learned, why do these PAs function?

Thank you for your positive words. We have **added further analysis about the factors associated with the various outcomes from the protected areas** in the sample which has allowed us to gain more insights into why certain protected areas function better than others, specifically protected areas with better environmental performance compared to their matched controls are in dryer, less agriculturally suitable areas (which we interpret as having lower opportunity costs for agricultural expansion and timber extraction), they also have higher area-adjusted budgets, while better wellbeing performance is associated with better agricultural suitability. We have revised the discussion to better highlight these insights. **Please see response to comment R1.02 for further details**.

We now also discuss win-wins in more detail:

> *"While protected areas consistently reduce environmental degradation, they are associated with significantly worse food security and material wellbeing changes among nearby communities (Fig. 3). Only a small subset of Ethiopian protected areas delivered 'win-win' outcomes (Fig. 4), often in places where local livelihoods were compatible with conservation (Supplementary Text S1). These findings make an important contribution to ongoing debates about the extent to which protected areas can be expected to deliver 'win-wins' in terms of positive impacts on both environmental and social wellbeing outcomes(Hockley et al., 2018; Li et al., 2024; Naidoo et al., 2019; Poudyal et al., 2018; Rakotonarivo et al., 2025; Woodhouse et al., 2022; Zafra-Calvo et al., 2019) and provide grounded evidence needed to inform protected area management or expansion decisions."*

R2.02 There have been some studies that have explored the same theme before:
https://www.sciencedirect.com/science/article/abs/pii/S0264837723000479 (Auliz-Ortez et al. 2023)
https://www.sciencedirect.com/science/article/pii/S0960982224006870 (Li et al 2024)
both missing from the ref list, so there is a need to convince the reader of why and how this study is novel/different and these papers also provide good food for thought for the discussion and how generalizable the findings might be beyond Ethiopia.

Of course, the reviewer is correct that other authors have explored the trade-offs and synergies between conservation effectiveness and wellbeing. However, our paper makes a novel contribution:

First, as noted by reviewer 1 '*although only a single-country study, it is convincing precisely because of the depth of analysis. It is an important contribution to this general area of research'*, we improve on global studies by focusing on a single country to provide much more comprehensive, accurate and context relevant analysis that improves applicability to policy:

- Previous work on synergies/trade-offs between conservation effectiveness and local development at global scales rely on expert opinion (Gatiso et al., 2022) or proxies (Li et al., 2024). For example, the reference Li et al (2024) highlighted by the reviewer assesses over 10,000 protected areas globally using nightlights as a development proxy; however, this proxy is not necessarily relevant at national scales where progress towards protected area targets are being implemented. Li et al. actually draw out Ethiopia as one of their top four countries that showed synergies between conservation and local economic growth (with synergies referring to similar or larger increase in natural land cover and nightlights). However, in least developed countries such as Ethiopia, nightlight intensity is near zero across much of the country (in 2000 radiance values ranged from 0-111 with a mean of 0.002, and in 2018 values ranged from 0-103 with a mean of 0.01) making this proxy insensitive to local economic variation. Looking at Figure 2 in Li et al. corroborates this, showing that Ethiopia's protected areas demonstrate no difference in nightlight change compared to the counterfactuals, and we argue this is an artifact of the proxy rather than a true reflection of the development changes. This limitation is likely to apply to many low-income countries included in Li et al. where low baseline development level makes it difficult to detect meaningful effects using global proxies. This highlights the importance of national scale analysis and using context-

specific indicators to produce our study more accurate and nuanced assessment of trade-offs between biodiversity conservation and human wellbeing.

Second, we improve on the scale of analysis compared to relevant previous work at national scales:

- **Existing studies often rely on data aggregated at larger spatial scales, rather than the household level data we used in our paper**. Ferraro et al (2011) and Den Braber et al (2024) both use census-tract level data, Morgans et al (2024) uses village-level, and Auliz-Ortiz et al (2023) used municipality level analysis. In contrast, our study maintains data at the household level and while both approaches report an overall average effect, they average over different units. We estimate the average effect on households using household microdata and household-specific exposure, which reduces spatial mismeasurement. Area-level studies estimate the average effect on places using tract/village/municipality means, giving equal weight to each area and relying on coarser exposure definitions.
- **Additionally, we include a broader range of outcome measures compared to many other trade-off/synergy quasi-experimental studies**. For example, most only use deforestation as an outcome for conservation effectiveness (Auliz-Ortiz et al., 2023; den Braber et al., 2024; Ferraro et al., 2011; Morgans et al., 2024), Miranda et al. (2016) expand slightly by also using forest disturbance. Others use poverty or marginality indexes which don't allow for breakdown of what dimensions of wellbeing are specifically being affected, such as Ferraro et al. (2011) in Costa Rica and Auliz-Ortiz et al (2023) in Mexico. Others rely on predominantly monetary based outcomes such as Ferraro et al. (2011) use poverty headcount ratio for Thailand and Miranda et al. (2016) use income and poverty rate in Peru. We expand on this by assessing three environmental (change in forest, grassland, and agricultural land cover) and three social wellbeing (months of adequate food, dietary diversity and material wellbeing) outcomes.

We have updated the references we use in the introduction to summarise previous work on trade-offs between social and environmental outcomes from protected areas to include the two that the reviewer has highlighted and have clarified how our analysis contributes beyond these:

> *"A few studies have explicitly looked at trade-offs between environmental and social outcomes, however, many rely on data aggregated across large administrative units (Auliz-Ortiz et al., 2023; den Braber et al., 2024; Ferraro et al., 2011; Morgans et al., 2024), limited outcome indicators (Auliz-Ortiz et al., 2023; den Braber et al., 2024; Ferraro et al., 2011; Li et al., 2024; Miranda et al., 2016; Morgans et al., 2024), or global proxies for development (Li et al., 2024) that are insensitive to local variation, and household-level multidimensional analyses remain rare (Ghoddousi et al., 2022)."*

We have also engaged more with this literature throughout the main text with relevant additions including:

> *"Evidence from the Global South shows that simple 'win-win' narratives can be misleading with costs often borne locally, especially by marginalised groups (Woodhouse et al., 2022).*

> *"Studies also vary in scale; however, global syntheses pool highly diverse socio-ecological contexts, which can mask heterogeneity in outcomes and limit national policy relevance (Geldmann et al., 2019; Li et al., 2024; Naidoo et al., 2019)."*

> *"In low-income countries where rural poverty remains a considerable challenge, protected areas are increasingly expected to contribute to socio-economic development alongside conservation goals, despite environmental and social goals often conflicting with one another (Adams et al., 2023; Gatiso et al., 2022; Henry et al., 2022). In such contexts, there is little credible evidence of sustained positive social outcomes (Rakotonarivo et al., 2025) and transparent evaluation is needed to identify who bears the costs (Hockley et al., 2018; Poudyal et al., 2018)."*

“These findings make an important contribution to ongoing debates about the extent to which protected areas can be expected to deliver ‘win-wins’ in terms of positive impacts on both environmental and social wellbeing outcomes (Hockley et al., 2018; Li et al., 2024; Naidoo et al., 2019; Poudyal et al., 2018; Rakotonarivo et al., 2025; Woodhouse et al., 2022; Zafra-Calvo et al., 2019) and provide grounded evidence needed to inform protected area management or expansion decisions.”

R2.03 The figures are all very easy to understand and informative and summarizes the main findings in an elegant and insightful way.

We thank the reviewer for their positive remarks.

R2.04 My biggest concern is the main text. Considering the robustness of the analyses and the insightful ways of illustrating the findings, the main text feels under-developed and will need a substantial rewrite. Especially the Discussion needs more attention. The discussion should engage more with previous studies that have also explore the trade-off between conservation and wellbeing and also literature on who bears the costs of conservation. A possibility is also to consider how spatial conservation prioritization tools (Marxan, Zonation) could be used in the future to maximize species representation while minimizing opportunity costs, when expanding the PA network.

We thank the reviewer for this constructive feedback. We have substantially revised the Discussion and made revisions to the introduction and results framing to (1) engage more with previous work on trade-offs between conservation and wellbeing; (2) link more with the literature on who bears the cost of conservation (3) highlight the need for spatial conservation prioritisation to guide future decisions, and (4) generally add clearer synthesis throughout the discussion. Please see specific additions and changes below:

1. Engage more directly with prior trade-off studies:

   (**please see response to comment R2.02 where we outline substantial changes related to this topic**).

2. Link more with the literature on who bears the cost:

   (**please see response to comment R2.20 where we outline substantial changes related to this topic**).

3. Highlight the need for spatial conservation prioritisation:

   > *“Expanding protected areas in these regions would likely incur high local opportunity costs, compounding existing livelihood challenges and trade-offs (Green et al., 2013; Schleicher et al., 2019). Consistent with this, we find that environmental outcomes of protected areas are worse in wetter and more agriculturally suitable areas where agricultural expansion and timber extraction likely produce better returns (Lomax et al., 2024), while wellbeing outcomes are marginally better where agricultural suitability is greater. Therefore, future progress towards a more representative network will require careful spatial planning, using multi-objective spatial conservation prioritization tools to identify locations that help to deliver conservation goals at least cost to people (Arneth et al., 2023; Neubert et al., 2025)”*
   >
   > *“To bridge the global–local divide, conservation must reflect economic and institutional realities, with governments balancing land-use trade-offs through cross-sector collaboration and inclusive, livelihood-aligned spatial planning.”*

R2.05 line 67: or at least require more funding to compensate for adverse effects?

We have updated the sentence to the following (underlined shows additions):

> *"Without a clearer understanding of trade-offs, countries may be reluctant to support protected area expansion that risks harming local communities, or may require additional funding and international support to offset potential negative effects."*

R2.06 line 90: do you mean IUCN category II (i.e. not plural)?

This has been corrected.

R2.07 line 92: you have to keep in mind that at this stage, the reader has not yet read the data you were using and their reliability.

We have updated this sentence to the following (underlined shows additions):

> *"This differs from the 17% reported on the World Database on Protected Areas (Supplementary Table S1) because our updated dataset removes degazetted or duplicate areas and updates boundaries of downsized or merged areas."*

R2.08 line 106: same as before, the reader has no idea what "Ethiopia's multidimensional environmental space" is?

We have updated this sentence to the following (underlined shows additions):

> *"Protected areas also encompassed 33% of Ethiopia's multidimensional environmental space, defined as the range of climatic and environmental conditions summarised across 19 bioclimatic variables (Supplementary Figure S6)."*

R2.09 about representativeness, I wonder if your dataset could be used to also explore temporal trends in how "high and far" the PAs are located? i.e. are more recently established PAs located in areas of higher pressures?

We thank the reviewer for this interesting suggestion. In response we conducted an **additional analysis** to assess whether more recently established protected areas are located in areas experiencing greater human pressures.

New analysis

Using a representative sample of gridcells within the protected area network, we extracted covariates assumed to represent human pressures and used Spearman's rank to identify relationships with year of protected area establishment. We used a fixed time point for our human pressure measurements to avoid our results being driven by population growth and economic development (Supplementary Methods S1).

Results provide evidence of original interpretation

Our results show weak but statistically significant trends with more recently established protected areas being associated with higher population density and agricultural land cover and reduced travel time to cities. We update the main text results to include this, and link to a more detailed description in the supplementary material:

> *"Newer protected areas have generally been established in areas of higher human pressures (Supplementary Results S1)"*
>
> ***"Results S1** Newer protected areas are being established in areas of higher human pressure*

Spearman's rank correlations indicate that more recently established protected areas tend to be located in areas with higher population density (r_s = 0.24, $p < 0.001$), and more agricultural land (r_s = 0.24, $p < 0.001$). They are also weakly associated with shorter travel times to cities (r_s = –0.045, $p < 0.001$) and higher elevations (r_s = 0.096, $p < 0.001$) indicating they newer protected areas are generally being established in areas of higher human pressure."

Please also see **additional response to related comment R2.18**.

R2.10 line 127: you have to keep in mind that the reader has not yet read the methods, so you need to specify what the strict and less strict PAs are somewhere here in the main text (perhaps in first section of Results?).

As suggested, we have updated the first section of the results to specify the IUCN categories of the strict and less strict PAs are. We now say:

"As of September 2024, protected areas cover 9.4% of Ethiopia (Fig. 1A) of which 3.8% comprises strict protected areas (IUCN category II) with the remainder being less strict (IUCN categories IV and VI) (Supplementary Table S9).

R2.11 line 133: really odd opening of paragraph here, what does this refer to, what biodiversity outcomes? you just had a paragraph about pressures and land-use change in PAs, are you referring to this as biodiversity outcomes?

We have updated the opening sentence to the following:

"Although Ethiopia's protected area network was effective at resisting land cover changes across measured environmental outcomes, this success came with substantial local cost for social wellbeing."

We also have clarified the meaning of environmental outcomes and social wellbeing outcomes and ensured consistent use of terminology throughout the manuscript (**please see response to comment R2.14**)

R2.12 line 137: can you mention the covariates used for this matching here please

We have updated the start of the results section on protected area effectiveness to introduce the covariates used for matching, adding the text:

"Using a covariate-adjusted regression comparing statistically matched cells and households within and outside Ethiopia's protected area network, we accounted for key environmental and socio-economic confounders (Supplementary Figure S3, Supplementary Table S5), including elevation, slope, temperature, precipitation, agricultural suitability, access to cities, population, ethno-linguistic groups, and agriculture. For environmental outcomes we additionally included ecoregion and other baseline land cover variables (forest, grassland, majority land cover type)."

This updated text provides the reader with a clear understanding of the factors accounted for in our quasi-experimental approach while detailed methodological information remains in the Methods section and Supplementary Information.

R2.13 line 151: here you mention environmental outcomes, is this the same as the biodiversity outcomes? Define what you mean and be consistent throughout.

Considering both this comment and comment **R2.14**, we have updated the manuscript to ensure consistency throughout the text to refer to outcomes associated with land cover change as 'environmental outcomes' and we have now defined environmental outcomes in the first paragraph of the results section on protected area effectiveness as:

> *“three environmental outcomes (forest, grassland and agricultural land cover change)”.*

R2.14 line 158: what are these six effectiveness measures, you need to explain this clearly earlier on when setting the scene, since methods comes only later in the Nature format.

We have now clarified at the start of the results section on protected area effectiveness that:

> *“we assess this effectiveness across six measures: three environmental outcomes (forest, grassland and agricultural land cover change) and three social outcomes (months of adequate food, dietary diversity, and material wellbeing)”.*

R2.15 line 159: worth here to be specific about the nature of the trade-off, i.e. in favor of environmental outcomes or wellbeing?

The original wording of: “68% demonstrated trade-offs between environmental outcomes and wellbeing of local communities” referred to trade-offs in either direction.

We have now split this summary statistic into two to show the proportion of PAs experiencing trade-offs in each direction separately rewording to:

> *“68% demonstrated trade-offs between environmental outcomes and wellbeing outcomes (12 of the 17 protected areas experiencing trade-offs had effective environmental performance at the cost of wellbeing); 20% experienced win-win outcomes, and 12% experienced lose-lose outcomes (Fig. 4a).”*

R2.16 line 167: accounting for PA size, right? i.e. management costs per hectare? also think about economies of scales, see e.g. https://www.sciencedirect.com/science/article/pii/S0006320710004258 https://conbio.onlinelibrary.wiley.com/doi/full/10.1111/csp2.12697

This is a tricky issue which we discussed quite extensively as an authorship group. Following this reviewer comment we have **revised our approach to account for economies of scale in management costs**. We now account for protected area size by modelling total annual budget as a function of protected area size and using the residuals as an area-adjusted measure of financial input. This captures whether a protected area receives more or less funding than is expected given its size. We have updated the methods accordingly and have referenced the linked papers above.

Additionally, in response to a related comment from Reviewer 1 we have updated the model to include a wider variety of potential ecological and socio-economic predictors of protected area performance (**see response to R1.02**). Importantly, our results are consistent with our previous findings: budget remains an important predictor of environmental performance, but not of wellbeing performance.

R2.17 line 176: out of what options? need to clarify briefly, as methods come only later.

The text highlighting the options has now been made clearer, these options were defined with the help of Ethiopia’s protected area think tank:

> *“We asked 37 Ethiopian conservation professionals working in policy, research or practice (Supplementary Table S8) to rank three overarching priorities for Ethiopia’s protected area network: (i) expanding the network, (ii) making the existing network more effective, and (iii) carrying out additional research to guide improvements. Most respondents (77%) ranked effectiveness as their top priority, followed by research, with expansion ranked the lowest.”*

R2.18 line 217: see my earlier comment (**R2.09**), you could have done an analysis if this was the case so far in Ethiopia?

Thank you for this suggestion. In addition to conducting the analysis suggested in **comment R2.09,** we have **conducted a further additional analysis** to compare pressures across ecoregions relative to their protected area representation.

New analysis

We classified ecoregions as under- of over-represented based on whether their coverage in protected areas was below or above 9.4% (Ethiopia's current protected area land coverage). We extracted weighted samples from each group based on how far under or over-represented the ecoregions were in the current network, and compared potential pressures including population, agricultural land cover, agricultural suitability, accessibility, and elevation using a blocked permutation test to account for spatial correlation (full details are in Supplementary Method S2)

Main results

The results show that more underrepresented ecoregions tend to occur in areas with greater agricultural land cover, higher population density and better access to cities, we include details of the statistical inference in Supplementary Results S2.

> *"**Results S2** Human and land-use pressures across under- and over- represented ecoregions*
>
> *Blocked permutation tests show that underrepresented ecoregions had higher agricultural land cover (Underrepresented = 19.68, Overrepresented = 9.76; p = 0.002, FDR-adjusted p = 0.005), shorter travel time to cities (Underrepresented = 538.14, Overrepresented = 638.92; p = 0.002, FDR-adjusted p = 0.005), and higher population density (Underrepresented = 64.37, Overrepresented = 47.14; p = 0.030, FDR-adjusted p = 0.050). We found no evidence of differences for agricultural suitability (FDR-adjusted p = 0.47) or elevation (FDR-adjusted p = 0.47)."*

Main text updates

We have updated the 'ecological and taxonomic representativeness' results section text to highlight that:

> *"Gaps in the network disproportionately occur in more accessible areas with more agriculture and higher population densities (Supplementary results S2)"*

These patterns indicate that expanding protection to meet the 30-by-30 target is likely to involve greater social and land-use trade-offs. We have updated the discussion to reflect this:

> *"Expanding protected areas to pursue ecological representativeness may exacerbate trade-offs between environmental and social wellbeing outcomes. Ethiopia's underrepresented ecoregions (Fig. 2) are located in areas facing higher human pressures (Supplementary Results S2). For example, the Ethiopian montane grasslands and woodlands represents one of the most agriculturally productive areas in Ethiopia (Workie & Debella, 2018), and the Somali Acacia–Commiphora bushlands and thickets is among one of the most food insecure areas (Mohamed, 2017). Expanding protected areas in these regions would likely incur high local opportunity costs, compounding existing livelihood challenges and trade-offs (Green et al., 2013; Schleicher et al., 2019). Consistent with this, we find that environmental outcomes of protected areas are worse in wetter and more agriculturally suitable areas where agricultural expansion and timber extraction likely produce better returns (Lomax et al., 2024), while wellbeing outcomes are marginally better where agricultural suitability is greater. Therefore, future progress towards a more representative network will require careful spatial planning, using multi-objective spatial conservation prioritization tools to identify locations that help to deliver conservation goals at least cost to people (Arneth et al., 2023; Neubert et al., 2025). Protected areas have often been established in areas with low opportunity costs (Joppa & Pfaff, 2009; Venter et al., 2018), meaning that many countries – particularly lower-*

income countries (Waldron et al., 2022) – are likely to face similar challenges when considering ecologically representative protected area expansion."

R2.19 line 230: could have been said before, in general the discussion is just a repetition of results and points raised earlier, little useful synthesis and a missed chance to enter a dialogue with previous literature.

Thank you for this helpful pointer. We have **rewritten the discussion** to cut repetition of the results, add synthesis and enter a clearer dialogue with the previous literature. Specifically we have:

1. Reduced the recap of numeric results and kept the discussion interpretive, the opening of the discussion now highlights the take home message without going into numerical values

2. Added synthesis to describe potential reasons behind our findings:

> *"Only a small subset of Ethiopian protected areas delivered 'win-win' outcomes (Fig. 4), often in places where local livelihoods were compatible with conservation (Supplementary Text S1)."*
>
> *"Expanding protected areas in these regions would likely incur high local opportunity costs, compounding existing livelihood challenges and trade-offs (Green et al., 2013; Schleicher et al., 2019). Consistent with this, we find that environmental outcomes of protected areas are worse in wetter and more agriculturally suitable areas where agricultural expansion and timber extraction likely produce better returns (Lomax et al., 2024) while wellbeing outcomes are marginally better where agricultural suitability is greater. Therefore, future progress towards a more representative network will require careful spatial planning, using multi-objective spatial conservation prioritization tools to identify locations that help to deliver conservation goals at least cost to people(Arneth et al., 2023; Neubert et al., 2025)"*
>
> *"While we find that higher area-adjusted budgets are associated with improved environmental outcomes, they show no detectable relationship with wellbeing performance. This is consistent with evidence that protected area funding and management capacity (e.g., enforcement and habitat management) underpin ecological effectiveness(Coad et al., 2019), but that insufficient resources are being allocated to strategies that support livelihood improvements for surrounding communities (Admasu et al., 2020; Oldekop et al., 2016; Rakotonarivo et al., 2025)"*
>
> *"Avoiding negative social impacts of protected areas will require additional, targeted social investments that go beyond core protected area budgets"*

3. Included additional, and engaged more, with existing literature:

   (**please see responses to comments R2.02 and R2.20 where we outline substantial changes related to this topic**).

R.2.20 line 283: I am missing a bit better referencing to previous literature looking into who bears the cost of conservation, e.g https://peerj.com/articles/5106/ (Poudyal et al., 2018) but I think Neal Hockley has worked on this also more generally so worth to look into.

We cited this paper in the introduction originally but have now also added it here. Additionally, we have added references to a wider variety of literature on who bears the cost of conversation including additional relevant references from Neal Hockley's work, among others working on this topic:

Manuscript text updates (additions underlined)

Introduction:

- *"Evidence from the Global South shows that simple 'win-win' narratives are often misleading with costs often borne locally, especially by marginalised groups (Woodhouse et al., 2022)."*

- *"To meet the more ambitious 30-by-30 target, and ensure ecologically representative networks, countries will need to expand into under-represented ecoregions which risks increasing competition with alternative land use such as agriculture. Consequently, both trade-offs with local food supplies and the number of people impacted by protected areas is expected to increase dramatically(Rakotonarivo et al., 2025; Schleicher et al., 2019)."*

- *Global syntheses pool highly diverse socio-ecological contexts, which can mask heterogeneity in outcomes and limit national policy relevance (Geldmann et al., 2019; Li et al., 2024; Naidoo et al., 2019).*

- *"In low-income countries where rural poverty remains a considerable challenge, protected areas are increasingly expected to contribute to socio-economic development alongside conservation goals, despite environmental and social goals often conflicting with one another (Adams et al., 2023; Gatiso et al., 2022; Henry et al., 2022). In such contexts, there is little credible evidence of sustained positive social outcomes (Rakotonarivo et al., 2025) and transparent evaluation is needed to identify who bears the costs (Hockley et al., 2018; Poudyal et al., 2018)."*

- *"Without a clearer understanding of trade-offs, countries may be reluctant to support protected area expansion that risks harming local communities, or may require additional funding and international support to offset potential negative effects (Rakotonarivo et al., 2025)."*

Discussion:

- *"Ethiopia faces the challenge of meeting ambitious conservation targets while substantial proportions of its population experiences undernourishment and multidimensional poverty (Global Hunger Index, 2024; World Bank, 2025). In many such settings, agricultural development is justifiably a top policy priority (Federal Democratic Republic of Ethiopia Planning and Development Commission, 2021; World Food Programme, 2025) which can conflict with conservation goals."*

- *"Chronic underfunding and capacity shortfalls in protected areas around the world (Fisher et al., 2023) makes it difficult to see how dramatic protected area expansion can be achieved in ways which deliver effective conservation without undermining local wellbeing."*

- *"While we find that higher area-adjusted budgets are associated with improved environmental outcomes, they show no detectable relationship with wellbeing performance. This is consistent with evidence that protected area funding and management capacity (e.g., enforcement and habitat management) underpin ecological effectiveness (Coad et al., 2019), but that insufficient resources are being allocated to strategies that support livelihood improvements for surrounding communities (Admasu et*

al., 2020; Oldekop et al., 2016; Rakotonarivo et al., 2025). Avoiding negative social impacts of protected areas will require additional, targeted social investments that go beyond core protected area budgets (Hockley et al., 2018)."

- *"The Ethiopian protected area network is already severely underfunded (Van Zyl, 2015). In this context, prioritising improvements to the existing network over expansion (as suggested by Ethiopian stakeholders) is sensible (Adams et al., 2019)"*

R2.21 line 291: when gazetted?

The gazettement year for each of the 12 protected areas referred to here is provided in Supplementary Table S1, and we direct readers to this. We feel listing gazettement dates of 12 protected areas in main text would interrupt the narrative flow. We have now made it clearer that this information is available updating the text to:

> *"(further details on gazettement years and area changes are provided in Supplementary Table S1)"*

R2.22 line 294: how did you deal with the overlaps?

We have clarified this sentence as follows:

> *"We also exclude from the WDPA database 57 National Forest Priority Areas (NFPAs), which identify areas with important forest resources but do not meet the IUCN definition of a protected area and often have little natural forest remaining (Ethiopian Biodiversity Institute, 2023; Reusing, 1998). Twenty-six of these NFPAs overlap at least partially with gazetted protected areas (Supplementary Figure S1); for these, we retain the overlapping portions to ensure that all legally recognised protected areas are included."*

R2.23 line 302: why is this more accurate?

In the World Database on Protected Areas, the "designation year" field often records the date of the most recent formal legal designation or IUCN classification update, not necessarily when the area first started being protected. However, the ecological and social effects of a PA — changes in deforestation rates, land use, human settlement, etc. — depend on when on-the-ground protection began, not when official designation or redesignation occurred.

We have now reflected this more clearly in the text:

> *"For year of establishment, we use the earliest record of the protected area either regionally or nationally, as this more closely reflects when on-the-ground protection began, whereas the designation year in the WDPA often refers to later legal updates or IUCN reclassifications."*

R2.24 line 310: what is this environmental space, please explain better

We have updated the text to define what we mean by environmental space:

> *"Representativeness of Ethiopia's protected area network in environmental space, defined as the range of climatic and environmental conditions across the country summarised through a principal component analysis of 19 bioclimatic variables, was also assessed (Supplementary Methods S1)."*

R2.25 line 330: here comes the definitions I was missing, use this terminology consistently in the main text.

We have updated the text to ensure consistent use of terminology.

R2.26 line 334: give more info on data for the outcomes and how it was done. otherwise not possible to evaluate how rigorous.

Due to word count limitations this information is provided in supplementary table S2 which outlines the three environmental (forest, grassland and agricultural land cover change) and three social wellbeing (months of adequate food, dietary diversity, material wellbeing) outcome variables used to understand protected area effectiveness.

This table includes columns for (i) the outcome variable, (ii) name of the dataset used and reference, (iii) time period, (iv) details of how each outcome was measured, defined and the resolution, and (v) the expected direction of change if protected areas are effective.

R2.27 line 344: sure, but how about the time period? doesn't match with your PA polygons? Also the sample seems small, could you put it on a map in suppl so the reader could see the spatial extent and possible spatial aggregation?

For the wellbeing analysis, we used the longest continuous household panel available from Ethiopia's Living Standards Measurement Study (LSMS), which spans 2011 to 2016. Unfortunately, the subsequent 2018 LSMS round was not a continuation of the same panel but a new, cross-sectional sample, meaning that we could not directly compare household level changes from 2011-2018. Therefore, 2011–2016 represents the only period over which we can robustly assess changes in household wellbeing. We now highlight this as a limitation in our discussion:

> *"We also note timing mismatches between environmental (2000–2020/21) and wellbeing (2011–2016) outcomes. Although the shorter wellbeing timeframe may miss longer-run effects, it uses the longest household panel available which allows us to track the same households, reduces bias from migration, and keeps both outcome sets within the same post-2000 policy regime."*

While we agree that the initial sample size of 3699 households is smaller than we would like, it is large relative the literature. For comparison, we extracted sample size data from Kandel et al (2022) meta-analysis which looked at studies that had assessed the impact of protected areas on income, and of 22 studies which were carried out at the household level 81% had a smaller sample size than our study, and for 61% the sample size was an order of magnitude smaller. Additionally, many of these studies only assessed social outcomes of protected areas, whereas Our approach additionally combines this with environmental outcomes.

In response to this comment, we have also added a map of the enumeration area locations in supplementary figure S4.

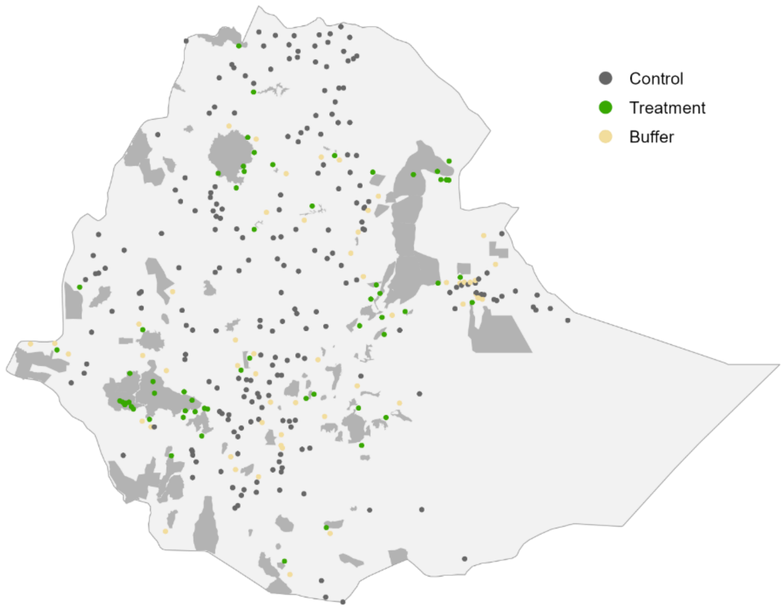

Figure S4. Map of enumeration area locations from households surveyed in both the 2011 and 2016 Living Standards Measurement Survey. The control pool are more than 20km from a protected area, treatment pools are less than 10km from a protected area and buffer pools are in between.

R2.28 line 347: did you mask them out from serving as control sites for the matching?

We did not mask out National Forest Priority Areas (NFPAs) from serving as control sites. Empirically, forest cover change in NFPAs was not different from other non-protected areas, so we decided on balance to include them in the donor pool of sites from which controls could be selected. Retaining NFPAs in the donor pool increased the pool of available matches, improving covariate balance and the robustness of our quasi-experimental matching.

R2.29 line 370: what period of instability, you need to state this specifically, ideally already in the main text

We have updated the text to provide further explanation on the period of instability which is described in more detail in Supplementary Figure S2. The new text reads:

> *"We use the year 2000 as this represents the period following high instability under the Derg regime and the subsequent targeted exploitation of protected areas after its fall, during which there was limited funding for conservation (Supplementary Figure S2). This period of instability effectively acted as a reset for protected area management in Ethiopia, before the relatively more stable post-2000 period where management has been more aligned with the goals of the Convention on Biodiversity."*

R2.30 line 392: please specify what you mean with random offset here, not just in SI.

We have added more detail regarding the offset into the main text:

> *"For wellbeing outcomes, due to household coordinates being randomly offset by 0-2km to maintain participant confidentiality (Supplementary Methods S6), covariate data were aggregated across a 2 km buffer around each household unit."*

R2.31 line 476: needs to be budget per hectare, not overall budget, see previous comment and references provided.

Please see response to the previous comment referred to here (R2.16). The methods and analysis have been updated.

Reviewer #3 (Remarks to the Author):

R3.01 This manuscript assesses the representativeness of the protected areas network in Ethiopia, and attempts to quantify the impact of these protected areas on land cover and human wellbeing (food intake and assets). It is well-written, and the results are interesting. A lot of work has been put into this work. However, while the results would be of great interest to a field journal, I believe the contribution is not substantial enough for Nature Ecology & Evolution. Among all policy instruments, protected areas have been the most widely studied in the academic literature. Their (partial) ability to limit the expansion of the human footprint has been widely documented. The use of socio-economic surveys is less common, but the question of the possible negative effect on neighboring communities has also been quantified in several studies (e.g., Naidoo et al. 2019). The existing literature could be largely refined, notably by adopting more robust methodologies. In this manuscript, however, the authors mostly replicate the existing methodology without advancing it. Hence, the paper does not bring much thematic or methodological contribution.

We agree that the effectiveness of protected areas has been widely studied (albeit with varying degrees of robustness), however this reflects the importance of the topic. We strongly believe that our paper is of broad interest and is appropriate for *Nature Ecology and Evolution*, we have revised the paper to make the original contribution more explicit. We highlight the novelty of the paper explicitly below.

1. Environmental and wellbeing outcomes are rarely assessed together

As this reviewer notes, studies examining both environmental and social wellbeing outcomes are rare. The paper highlighted by the reviewer (Naidoo et al., 2019), for example, looks only at social outcomes. Most assessments of protected area effectiveness using quasi-experimental approaches (Andam et al., 2008; Black & Anthony, 2022; Bowker et al., 2017; Brodie et al., 2023; Canavire-Bacarreza et al., 2018; Eklund et al., 2016; Gaveau et al., 2009; Geldmann et al., 2019; Keane et al., 2020; Meng et al., 2023; Naidoo et al., 2019; Wauchope et al., 2022) evaluate either ecological or social outcomes, rarely both. This means that there are very few studies which explicitly assess environmental-social wellbeing trade-offs.

2. Social outcomes are rarely assessed at the household level

Some studies have assessed trade-offs, however for social wellbeing outcomes these often rely on proxies or spatially aggregated data. Li et al. (2024) combine forest-loss data with night-time lights as a human development proxy. However, such proxies are often uninformative in least developed country contexts such as Ethiopia. National scale analyses rely on aggregated data (e.g. Ferraro et al. 2011; Den Braber et al. 2024 at census-tract level; Morgans et al. 2024 at village-level; Auliz-Ortez et al. 2023 at municipality-level), which, as noted by Morgans et al. can mask local heterogeneity. **See response to R2.02 for more detail**. We update the text to highlight this:

> *"A few studies have explicitly looked at trade-offs between environmental and social outcomes, however, many rely on data aggregated across large administrative units (Auliz-Ortiz et al., 2023; den Braber et al., 2024; Ferraro et al., 2011; Morgans et al., 2024), limited outcome indicators (Auliz-Ortiz et al., 2023; den Braber et al., 2024; Ferraro et al., 2011; Li et al., 2024; Morgans et al., 2024), or global proxies for development (Li et al., 2024) that are insensitive to local variation, and household-level multidimensional analyses remain rare (Ghoddousi et al., 2022)"*

3. Many studies rely on inaccurate protected area data

Global studies, such as Naidoo et al. (2019) and Li et al. (2024) typically rely on protected area boundaries from the World Database of Protected Areas (WDPA), which often contain outdated, incomplete or simply incorrect delineations. We improve upon this by using manually and comprehensively validated PA polygons from the Ethiopian Wildlife Conservation Authority, ensuring accurate classification of whether areas are exposed to the treatment, therefore enabling much greater confidence in causal inference. We show that in Ethiopia, nationally validated polygons differ substantially from the WDPA database with an absolute difference in percentage land coverage of 7.6 percentage points (17% on the WDPA vs 9.4% with nationally validated polygons) which is a valuable contribution in its own right as highlights the problems with the WDPA.

4. Improving policy relevance with individual protected area level insights

We extend standard matching approaches by using linear-model weights (LMW) to rebalance matched samples at the individual-PA level, moving from network-wide to PA-specific estimates of average treatment effects. This disaggregation enables classification of PA outcomes (win–win, win–lose, lose–lose) and allows us to assess potential drivers of performance, making our results more policy relevance.

5. Previous studies have not linked protected area representativeness, effectiveness, and equity

This paper does a lot more than just examine trade-offs between environmental and social outcomes from protected areas. We link protected area extent, ecological representativeness and impact effectiveness – three dimensions that to our knowledge have not been assessed together. This integrated perspective provides a more complete view of conservation performance and progress toward multiple dimensions of the 30x30 target. We have now highlighted this in the first paragraph of our introduction:

> *"While attention has largely been focused on area coverage (Bingham et al., 2019), both 30-by-30 and its predecessor, Aichi Target 11, also require protected areas to be ecologically representative, well connected, effectively managed and equitably governed (Convention on Biological Diversity, 2022) – dimensions that are far less often systematically assessed or reported (Gannon et al., 2019)"*

6.Conservation policies and decisions are made nationally

Progress towards global conservation targets such as the high-profile 30-by-30 target requires national action. By taking a detailed look at Ethiopia's protected area network, how expansion to its current extent has impacted both environmental and social wellbeing outcomes, and national appetite for further expansion, we provide valuable insight about what will need to change for the target to be met. In doing so, we also contribute to a broader understanding of how conservation outcomes vary across contexts and how progress towards global conservation commitments requires national level data to be accurately assessed.

R3.2 In particular, the authors use statistical matching to correct for the possible non-random location of protected areas. While doing so is better than performing naïve comparisons between treated and control observations, matching has also been shown to be an extremely flawed method. While the authors demonstrate that the direction of the effect holds for many matching procedures, their figures also indicate that the point estimate varies significantly depending on the choice of model.

We appreciate the opportunity to clarify our rationale for using statistical matching to estimate the impact of protected areas, which we fully agree has limitations.

Protected areas are not randomly located in the landscape. It would be unethical as well as impractical to randomly allocate protected areas, which makes using randomized control trials to quantify protected area impacts impossible. While, at least in theory, a natural experiment may occur where protected area allocation is driven by factors unconnected to the outcomes of interest (land cover change or wellbeing), we don't know any examples where such instrumental variables control protected area allocation, and they certainly don't exist in the Ethiopian context. This therefore rules out the option of using instrumental variables. Regression discontinuity designs are increasingly being used in agricultural and environmental economics (Wuepper & Finger, 2023a) and could potentially be used to evaluate protected areas using the boundary as a spatial cut-offs rather than the more normal assignment thresholds (for example: Neal, 2024). However, this depends on the assumption that conditions are comparable on both sides of the boundary which is not the case in Ethiopia's protected areas. Additionally, this approach would only capture local effects at the boundary, whereas our study aimed to assess national scale impacts.

For these reasons, quasi-experimental approaches, such as using statistical matching to control for observed confounders (covariates driving both exposure to the treatment and outcomes of interest), have become the standard approach to evaluate the effectiveness of protected areas (Schleicher et al 2020; Geldmann et al 2025). Statistical matching has been applied in several recent *Nature Ecology and Evolution* papers including Berger et al., 2025 and den Braber et al., 2024. We recognise of course that matching cannot eliminate all sources of bias. Crucial, it rests on the assumption that there are no important unobserved confounders (Geldmann et al., 2025; Jones et al., 2022). In addition, matching results can be sensitive to somewhat arbitrary specification choices (King and Nielsen, 2019). However, we address these limitation in the following ways:

1. We use context-appropriate directed acyclic graphs to identify which covariates are needed to block back door pathways (shared associations that may create misleading correlations) between protection and each outcome that may otherwise affect our causality claims. This provided us with a context-specific set of observable covariates to match on, so that treated and control locations are comparable on the factors that jointly influence protected area locations and our outcomes, thereby justifying conditional ignorability (i.e. once we condition on these covariates, assignment to protection is as-if random with respect to the outcomes)

2. We acknowledge that there may be hidden confounders. However, we test the sensitivity of our results to these hidden confounders by applying sensitivity analysis using *sensemakr* (Cinelli & Hazlett 2020) to quantify how strong an unobserved confounder would have to be to nullify our results. We show an unobserved confounder would have to explain at least nine times more residual variance than our strongest observed covariate to overturn our findings. This gives us confidence that our findings are not vulnerable to hidden confounders.

3. We carry out post-matching diagnostic checks including the proportion of treated units retained after matching and standardised mean differences for all the covariates. These checks show improved balance while retaining sufficient treated units for inference. We follow the reporting standards presented in the highly cited paper: Schleicher et al. (2022).

4. We combine matching with covariate-adjusted regression to address any residual imbalance in the matched sample when estimating our effects.

5. We compare results across multiple alternative matching specifications. Our primary matching design is the "best match" that retains the full covariate set meaning it achieves the greatest covariate balance while maintaining the most treated units. Our primary approach therefore thus minimises residual confounding providing the most credible estimate of the causal effect. We compare this against multiple admissible alternatives to demonstrate robustness. Variation in the magnitude of effect across specifications is expected in complex social-

ecological systems but it does not undermine the overall conclusions which are highly consistent.

We have now **added an additional supplementary methods section**, which we refer to in the ‘quasi-experimental design’ section of our methods:

“Methods S9 Justification for the use of matching methods

Protected areas are not randomly located and are often biased towards areas with lower opportunity costs, which have particular biophysical and socioeconomic characteristics which may also influence the outcomes of interest (Venter et al., 2018). Simple comparisons between protected and unprotected areas are therefore inherently biased. Evaluating the causal impacts of protected areas, requires constructing a credible counterfactual (what would have happened if protection had not been put in place) which can be achieved through the use of quasi-experimental methods. Common approaches included statistical matching, difference-in-differences, synthetic control, instrumental variables and regression discontinuity and each of which relies on specific assumptions (O’Garra et al., 2025; Schleicher et al., 2020).

The suitability of different quasi-experimental approaches depends on the nature of the intervention being assessed, the available data and the spatial scale of inference (O’Garra et al., 2025; Schleicher et al., 2020). In Ethiopia, protected areas have been established gradually over decades through context-specific decisions rather than a consistent eligibility threshold. There is no variable that plausibly influences protected areas designation but does not affect the outcomes of interest (land cover and social wellbeing changes) other than through protection. This rules out a valid instrumental variables (Bastardoz et al., 2023) or regression discontinuity methods (Wuepper & Finger, 2023b). Synthetic control methods are best suited to discrete treatment event such as a policy or a single protected area with a specific start date (O’Garra et al., 2025). Difference-in-differences approaches require temporally consistent pre-and post-intervention data at comparable spatial resolution for both treated and untreated units (Fredriksson & Oliveira, 2019), which were unavailable for our outcomes.

Among quasi-experimental methods, statistical matching combined with post-matching covariate-adjusted regression was the most appropriate design for our data and research questions. This approach is widely used in conservation impact evaluation (Geldmann et al., 2025; Schleicher et al., 2020), and provides a transparent and replicable way to reduce selection bias by constructing a comparable sample of treatment and control units that are statistically similar based on observable covariates. To ensure robust causal inference from matching, we followed established best practice guidelines (O’Garra et al., 2025; Schleicher et al., 2020): all covariates likely to influence both protected area placement and the outcomes were included; balance was assessed post-matching and post-matching; covariate-adjusted regression was applied to reduce residual bias; and multiple matching algorithms were applied to test sensitivity to specification choice. While matching cannot account for potential unobserved confounders and is sensitive to the matching specification used, we explicitly assess these limitations. We conducted sensitivity analyses using sensemakr (Cinelli et al., 2024) to quantify the amount of variance in the treatment and outcome an unobserved confounder would need to explain compared to a benchmark observed covariate before it nullified the result.”

R3.3 As the extent of protected areas has increased since the 2000s, why haven’t we focused on these protected areas and estimated a Difference in Difference / BACI model? This would help you

relax several hypotheses to claim causality. (I understand that it would have been more complicated for the socio-economic outcomes with only data points at two dates.

The reviewer raises an important point which we discussed at length when developing the analysis. Including only the protected areas established after 2000 would limit the analysis to only 15 of the 79 protected areas, equating to around 1.3% of Ethiopia's land area or 15% of the total protected area network. We could have focused just on this subset but this would say much less about the impacts of the protected area network, and would also limit our ability to detect trade-offs due to the small sample size. Additionally, we concluded with our Ethiopian partners that this and lacks relevance to the overall challenges facing Ethiopia's protected areas.

Our chosen design targets policy relevance and coverage. We analyse 64 protected areas (approximately 85% of the network) and evaluate post-2000 management effects on environmental and household outcomes while including only PAs established before the outcome windows to avoid post-treatment bias. The year-2000 baseline is motivated by Ethiopia's institutional break: the instability under the Derg and the subsequent period of protected area exploitation resulted in a de facto management "reset"; from 2000 onward funding and objectives aligned more closely with Convention on Biodiversity goals (supplementary figure S2). We formalised the causal logic with directed acyclic graphs in supplementary figure S3.

R3.4 A handful of more precise comments regarding the use of LSMS data, which might be the most original contribution of this paper:
« Measuring change using the panel data ensures any patterns we see are not due to people immigrating or emigrating from the area". I believe this claim is not correct. Yoi have attrition in your data, and this attrition can be different across treatments.

The reviewer raises a good point. As noted in the original methods, some households were lost between survey rounds. We have now quantified attrition rates and found them to be similar between treatment (14%) and control (12%) groups. There is of course a chance that people with different characteristics were more or less likely to be lost to follow up in either treatment or control. However, we have checked whether months of adequate food security in 2011 differs between households which were lost between survey rounds and those that remained and found no significant difference between the groups so we do not believe this is to be a substantial issue.

We now acknowledge attrition as a caveat:

> *"Measuring change using the panel data reduces bias from people immigrating and emigrating from an area, however it does not fully eliminate bias due to attrition (Supplementary Methods S7)"*

And have also added the attrition rates and comparison statistics into Supplementary Methods S7.

> *"Attrition rates between survey years were low and similar (14% and 12% respectively) between households close to protected areas (within 10km) and further away (more than 20km). While attrition can bias estimates, the low rates and small difference suggest only limited potential for this. Additionally, there is no significant difference in months of adequate food in 2011 between households that were lost over survey rounds and those that remained ($t_{595.6} = 1.61$, $p = 0.11$)."*

R3.5 From your methodology, you don't seem to have used the sampling weights of LSMS, which are necessary to produce unbiased results.

We thank the reviewer for raising this point. We agree that survey sampling weights are necessary when using the full LSMS dataset to obtain nationally representative estimates. However, our primary analysis uses 1:1 propensity score matching to compare households close to protected areas (treatment) with matched controls, which creates a subsample of the original survey. This subsample composed of statistically similar treatment and control units is intentionally designed for internal

comparability and causal inference, not for population representativeness. Importantly, our analysis focuses on estimating the Average Treatment effect on the Treated (ATT) — i.e., the effect of proximity to protected areas on households that are actually near protected areas — rather than the population Average Treatment Effect (ATE). For ATT estimation, the goal is internal validity within the matched sample, and survey weights are not required. Applying the original sampling weights, which reflect survey design probabilities for the full sample, would be inappropriate and could bias covariate balance between treated and control units. Accordingly, our estimates reflect causal effects for the matched treated sample—households living near protected areas—rather than for the national population represented by the LSMS.

Nonetheless, as a robustness check, we re-estimated our models using normalized LSMS sampling weights in the matched sample. The overall results and estimated effects of proximity to protected areas remain consistent: for example, the negative effect on household income (MINS_change) and assets (asset_change) persists and remains statistically significant, while the effect on dietary diversity (HDDS_change) remains small and not significant (please see the table below). This confirms that our main conclusions are robust to survey weighting.

Outputs	Analysis	Estimate	p-value
**Months of adequate food**	Original	-1.23	<0.001
	Incl. sampling weights	-1.38	<0.001
**Dietary diversity**	Original	0.12	0.47
	Incl. sampling weights	0.24	0.23
**Asset ownership**	Original	-1.21	<0.001
	Incl. sampling weights	-1.67	<0.001

R3.6 You mention that you detail your LSMS outcomes in Table 1 but I can't find the table in the manuscript.

We thank the reviewer for spotting this oversight, the table was moved to Supplementary Table S2 due to limitations on the number of items in the main text, however we mistakenly did not update the reference to this in the text. This has now been corrected.

R3.7 For both LSMS and ecological data, you could consider clustering your standard errors at a higher level than the matched pairs. This could be the enumeration area for LSMS data or some administrative boundaries.

Our choice of clustering follows design-based from Abadie et al. (2023). Their framework clarifies that clustering should reflect the assignment mechanism to treatment and controls and how clusters are part of the treatment effect estimation.

In our study, the Average Treatment Effect on the Treated (ATT) is identified by within subclass comparisons that pair treated units with closely matched control units based on a wide variety of relevant covariates. Clustering at the matched-subclass level therefore aligns the standard error calculation with the groups that actually generate the estimate. Additionally, Abadie et al. show that higher level clustering can be over-conservative and produce unstable standard errors, especially in the cases when cluster sizes/treatment shares are uneven, which is the case in our matched sample. For example, with LSMS data enumeration area cluster sizes within the matched sample range from 1 to 12 households and households within any enumeration area are either all treatment or all control. Likewise, with ecological data, clustering by administrative boundaries would involve very few clusters which would be highly uneven in both size and treated share. For these reasons, we retain subclass clustering rather than clustering at the EA or administrative-unit level, and we have cited Abadie et al. in the text.

References

Adams, V. M., Chauvenet, A. L. M., Stoudmann, N., Gurney, G. G., Brockington, D., & Kuempel, C. D. (2023). Multiple-use protected areas are critical to equitable and effective conservation. *One Earth*, *6*(9), 1173–1189. https://doi.org/10.1016/j.oneear.2023.08.011

Adams, V. M., Iacona, G. D., & Possingham, H. P. (2019). Weighing the benefits of expanding protected areas versus managing existing ones. *Nature Sustainability*, *2*(5), 404–411. https://doi.org/10.1038/s41893-019-0275-5

Admasu, S., Tadele, H., Tessema, M., & Tefera, Z. (2020). Underfunding, the challenge of federally managed protected areas of Ethiopia. *International Journal of Biodiversity and Conservation*, *12*(4), 316–325. https://doi.org/10.5897/IJBC2020.1431

Andam, K. S., Ferraro, P. J., Pfaff, A., Sanchez-Azofeifa, G. A., & Robalino, J. A. (2008). Measuring the effectiveness of protected area networks in reducing deforestation. *Proceedings of the National Academy of Sciences*, *105*(42), 16089–16094. https://doi.org/10.1073/pnas.0800437105

Armsworth, P. R., Cantú-Salazar, L., Parnell, M., Davies, Z. G., & Stoneman, R. (2011). Management costs for small protected areas and economies of scale in habitat conservation. *Biological Conservation*, *144*(1), 423–429. https://doi.org/10.1016/j.biocon.2010.09.026

Arneth, A., Leadley, P., Claudet, J., Coll, M., Rondinini, C., Rounsevell, M. D. A., Shin, Y.-J., Alexander, P., & Fuchs, R. (2023). Making protected areas effective for biodiversity, climate and food. *Global Change Biology*, *29*(14), 3883–3894. https://doi.org/10.1111/gcb.16664

Auliz-Ortiz, D. M., Arroyo-Rodríguez, V., Mendoza, E., & Martínez-Ramos, M. (2023). Are there trade-offs between conservation and development caused by Mexican protected areas? *Land Use Policy*, *127*, 106581. https://doi.org/10.1016/j.landusepol.2023.106581

Bassi, M., & Tache, B. (2011). The community conserved landscape of the Borana Oromo, Ethiopia. *Management of Environmental Quality: An International Journal*, *22*, 174–186. https://doi.org/10.1108/14777831111113365

Bastardoz, N., Matthews, M. J., Sajons, G. B., Ransom, T., Kelemen, T. K., & Matthews, S. H. (2023). Instrumental variables estimation: Assumptions, pitfalls, and guidelines. *The Leadership Quarterly*, *34*(1), 101673. https://doi.org/10.1016/j.leaqua.2022.101673

Berger, I., Kamble, A., Morton, O., Raj, V., Nair, S. R., Edwards, D. P., Wauchope, H. S., Joshi, V., Basu, P., Smith, B., & Dicks, L. V. (2025). India's agroecology programme, 'Zero Budget Natural Farming', delivers biodiversity and economic benefits without lowering yields. *Nature Ecology & Evolution*, 1–12. https://doi.org/10.1038/s41559-025-02849-7

Beyene, A. D., Mekonnen, A., Hirons, M., Robinson, E. J. Z., Gonfa, T., Gole, T. W., & Demissie, S. (2020). Contribution of non-timber forest products to the livelihood of farmers in coffee growing areas: Evidence from Yayu Coffee Forest Biosphere Reserve. *Journal of Environmental Planning and Management*, *63*(9), 1633–1654. https://doi.org/10.1080/09640568.2019.1679615

Bingham, H. C., Juffe Bignoli, D., Lewis, E., MacSharry, B., Burgess, N. D., Visconti, P., Deguignet, M., Misrachi, M., Walpole, M., Stewart, J. L., Brooks, T. M., & Kingston, N. (2019). Sixty years of tracking conservation progress using the World Database on Protected Areas. *Nature Ecology & Evolution*, *3*(5), 737–743. https://doi.org/10.1038/s41559-019-0869-3

Black, B., & Anthony, B. P. (2022). Counterfactual assessment of protected area avoided deforestation in Cambodia: Trends in effectiveness, spillover effects and the influence of establishment date. *Global Ecology and Conservation*, *38*, e02228. https://doi.org/10.1016/j.gecco.2022.e02228

Bowker, J. N., De Vos, A., Ament, J. M., & Cumming, G. S. (2017). Effectiveness of Africa's tropical protected areas for maintaining forest cover. *Conservation Biology*, *31*(3), 559–569. https://doi.org/10.1111/cobi.12851

Brodie, J. F., Mohd-Azlan, J., Chen, C., Wearn, O. R., Deith, M. C. M., Ball, J. G. C., Slade, E. M., Burslem, D. F. R. P., Teoh, S. W., Williams, P. J., Nguyen, A., Moore, J. H., Goetz, S. J., Burns, P., Jantz, P., Hakkenberg, C. R., Kaszta, Z. M., Cushman, S., Coomes, D., … Luskin, M. S. (2023). Landscape-scale benefits of protected areas for tropical biodiversity. *Nature*, *620*(7975), 807–812. https://doi.org/10.1038/s41586-023-06410-z

Canavire-Bacarreza, G., Diaz-Gutierrez, J. E., & Hanauer, M. M. (2018). Unintended consequences of conservation: Estimating the impact of protected areas on violence in Colombia. *Journal of Environmental Economics and Management*, *89*, 46–70. https://doi.org/10.1016/j.jeem.2018.02.004

Cinelli, C., Ferwerda, J., Hazlett, C., Tsao, D., Rudkin, A., & Ljubownikow, G. (2024). *sensemakr: Sensitivity Analysis Tools for Regression Models* (Version 0.1.6) [Computer software]. https://cran.r-project.org/web/packages/sensemakr/index.html

Coad, L., Watson, J. E., Geldmann, J., Burgess, N. D., Leverington, F., Hockings, M., Knights, K., & Di Marco, M. (2019). Widespread shortfalls in protected area resourcing undermine efforts to conserve biodiversity. *Frontiers in Ecology and the Environment*, *17*(5), 259–264. https://doi.org/10.1002/fee.2042

Convention on Biological Diversity. (2022). *Kunming-Montreal Global Biodiversity Framework. CBD/COP/15/L.25*. https://www.cbd.int/doc/c/e6d3/cd1d/daf663719a03902a9b116c34/cop-15-l-25-en.pdf

den Braber, B., Oldekop, J. A., Devenish, K., Godar, J., Nolte, C., Schmoeller, M., & Evans, K. L. (2024). Socio-economic and environmental trade-offs in Amazonian protected areas and Indigenous territories revealed by assessing competing land uses. *Nature Ecology & Evolution*, *8*(8), 1482–1492. https://doi.org/10.1038/s41559-024-02458-w

Eklund, J., Blanchet, F. G., Nyman, J., Rocha, R., Virtanen, T., & Cabeza, M. (2016). Contrasting spatial and temporal trends of protected area effectiveness in mitigating deforestation in Madagascar. *Biological Conservation*, *203*, 290–297. https://doi.org/10.1016/j.biocon.2016.09.033

Ethiopian Biodiversity Institute. (2023). *Final Technical Report—Small Technical Grant for Assessment 2021: Assessment of Ethiopia's protected area data quality for improved protected area management and governance* (No. STGA-EA-1422). http://geo.portal.ebi.gov.et/documents/905

Ethiopian Wildlife Conservation Authority. (2015). *Ethiopian Elephant Action Plant (2015-2025)*. https://ethiopias-elephants.com/wp-content/uploads/2020/10/20181003-FINAL-Ethiopian-Elephant-Action-Plan-IP-RMPB.pdf

Federal Democratic Republic of Ethiopia Planning and Development Commission. (2021). *Ten Years Development Plan: A Pathway to Prosperity*. https://nepad-aws.assyst-uc.com/agenda2063-national-development-plan/ten-years-development-plan-pathway-prosperity

Ferraro, P. J., Hanauer, M. M., & Sims, K. R. E. (2011). Conditions associated with protected area success in conservation and poverty reduction. *Proceedings of the National Academy of Sciences*, *108*(34), 13913–13918. https://doi.org/10.1073/pnas.1011529108

Fischer, A., Yitbarek, T., Czajkowski, M., Tadie, D., & Hanley, N. (2015). Trophy hunters' willingness to pay for wildlife conservation and community benefits. *Conservation Biology*, *29*, 1111–1121. https://doi.org/10.1111/cobi.12467

Fisher, J., Allen, S., Woomer, A., & Crawford, A. (2023). Protected areas under pressure: An online survey of protected area managers regarding social and environmental conservation target attainment and stakeholder conflicts. *World Development Sustainability*, *3*, 100084. https://doi.org/10.1016/j.wds.2023.100084

Fredriksson, A., & Oliveira, G. M. de. (2019). Impact evaluation using Difference-in-Differences. *RAUSP Management Journal*, *54*(4), 519–532. https://doi.org/10.1108/RAUSP-05-2019-0112

Gannon, P., Dubois, G., Dudley, N., Ervin, J., Ferrier, S., Gidda, S., Mackinnon, K., Richardson, K., Schmidt, M., Seyoum-Edjigu, E., & Shestakov, A. (2019). An update on progress towards Aichi Biodiversity Target 11. *PARKS*. https://doi.org/10.2305/IUCN.CH.2019.PARKS-25-2PG.en

Gatiso, T. T., Kulik, L., Bachmann, M., Bonn, A., Bösch, L., Freytag, A., Heurich, M., Wesche, K., Winter, M., Ordaz-Németh, I., Sop, T., & Kühl, H. S. (2022). Sustainable protected areas: Synergies between biodiversity conservation and socioeconomic development. *People and Nature*, *4*(4), 893–903. https://doi.org/10.1002/pan3.10326

Gaveau, D. L. A., Epting, J., Lyne, O., Linkie, M., Kumara, I., Kanninen, M., & Leader-Williams, N. (2009). Evaluating whether protected areas reduce tropical deforestation in Sumatra. *Journal of Biogeography*, *36*(11), 2165–2175. https://doi.org/10.1111/j.1365-2699.2009.02147.x

Geldmann, J., Jones, J. P. G., Wauchope, H., & Ferraro, P. J. (2025). Causal claims, causal assumptions and protected area impact. *Nature*, *638*(8052), E40–E41. https://doi.org/10.1038/s41586-024-08512-8

Geldmann, J., Manica, A., Burgess, N. D., Coad, L., & Balmford, A. (2019). A global-level assessment of the effectiveness of protected areas at resisting anthropogenic pressures. *Proceedings of the National Academy of Sciences*, *116*(46), 23209–23215. https://doi.org/10.1073/pnas.1908221116

Ghoddousi, A., Loos, J., & Kuemmerle, T. (2022). An Outcome-Oriented, Social–Ecological Framework for Assessing Protected Area Effectiveness. *BioScience*, *72*(2), 201–212. https://doi.org/10.1093/biosci/biab114

Global Hunger Index. (2024). *Ethiopia*. https://www.globalhungerindex.org/ethiopia.html

Green, J. M. H., Larrosa, C., Burgess, N. D., Balmford, A., Johnston, A., Mbilinyi, B. P., Platts, P. J., & Coad, L. (2013). Deforestation in an African biodiversity hotspot: Extent, variation and the effectiveness of protected areas. *Biological Conservation*, *164*, 62–72. https://doi.org/10.1016/j.biocon.2013.04.016

Henry, R. C., Arneth, A., Jung, M., Rabin, S. S., Rounsevell, M. D., Warren, F., & Alexander, P. (2022). Global and regional health and food security under strict conservation scenarios. *Nature Sustainability*, *5*(4), 303–310. https://doi.org/10.1038/s41893-021-00844-x

Hockley, N., Mandimbiniaina, R., & Rakotonarivo, O. S. (2018). Fair and equitable conservation: Do we really want it, and if so, do we know how to achieve it? *Madagascar Conservation & Development*, *13*(1), 3–5.

International Climate Initiative. (2018). *Restoring degraded coffee landscapes in Ethiopia*. https://www.international-climate-initiative.com/en/project/restoring-degraded-coffee-landscapes-in-ethiopia-18-iii-078-eth-a-restoring-coffee-landscapes/

Jones, J. P. G., Barnes, M., Eklund, J., Ferraro, P. J., Geldmann, J., Oldekop, J. A., & Schleicher, J. (2022). Quantifying uncertainty about how interventions are assigned would improve impact evaluation in conservation: Reply to Rasolofoson 2022. *Conservation Biology: The Journal of the Society for Conservation Biology*, *36*(6), e14007. https://doi.org/10.1111/cobi.14007

Joppa, L. N., & Pfaff, A. (2009). High and Far: Biases in the Location of Protected Areas. *PLOS ONE*, *4*(12), e8273. https://doi.org/10.1371/journal.pone.0008273

Keane, A., Lund, J. F., Bluwstein, J., Burgess, N. D., Nielsen, M. R., & Homewood, K. (2020). Impact of Tanzania's Wildlife Management Areas on household wealth. *Nature Sustainability*, *3*(3), 226–233. https://doi.org/10.1038/s41893-019-0458-0

Kiros, S., & Bekele, A. (2021). Assessment of conservation challenges in and around Gibe Sheleko National Park, southwestern Ethiopia. *Global Ecology and Conservation*, *32*, e01912. https://doi.org/10.1016/j.gecco.2021.e01912

Le Bouille, D., Fargione, J., & Armsworth, P. R. (2022). Spatiotemporal variation in costs of managing protected areas. *Conservation Science and Practice*, *4*(6), e12697. https://doi.org/10.1111/csp2.12697

Li, B. V., Wu, S., Pimm, S. L., & Cui, J. (2024). The synergy between protected area effectiveness and economic growth. *Current Biology*, *34*(13), 2907-2920.e5. https://doi.org/10.1016/j.cub.2024.05.044

Lind, J., Sabates-Wheeler, R., Caravani, M., Kuol, L. B. D., & Nightingale, D. M. (2020). Newly evolving pastoral and post-pastoral rangelands of Eastern Africa. *Pastoralism*, *10*(1), 24. https://doi.org/10.1186/s13570-020-00179-w

Lomax, G. A., Powell, T. W. R., Lenton, T. M., Economou, T., & Cunliffe, A. M. (2024). Untangling the environmental drivers of gross primary productivity in African rangelands. *Communications Earth & Environment*, *5*(1), 500. https://doi.org/10.1038/s43247-024-01664-5

Meng, Z., Dong, J., Ellis, E. C., Metternicht, G., Qin, Y., Song, X.-P., Löfqvist, S., Garrett, R. D., Jia, X., & Xiao, X. (2023). Post-2020 biodiversity framework challenged by cropland expansion in protected areas. *Nature Sustainability*, *6*(7), 758–768. https://doi.org/10.1038/s41893-023-01093-w

Miranda, J. J., Corral, L., Blackman, A., Asner, G., & Lima, E. (2016). Effects of Protected Areas on Forest Cover Change and Local Communities: Evidence from the Peruvian Amazon. *World Development*, *78*, 288–307. https://doi.org/10.1016/j.worlddev.2015.10.026

Mohamed, A. A. (2017). Food Security Situation in Ethiopia: A Review Study. *International Journal of Health Economics and Policy*, *2*(3), Article 3. https://doi.org/10.11648/j.hep.20170203.11

Morgans, C. L., Jago, S., Andayani, N., Linkie, M., Lo, M. G. Y., Mumbunan, S., St. John, F. A. V., Supriatna, J., Voigt, M., Winarni, N. L., Santika, T., & Struebig, M. J. (2024). Improving well-being and reducing deforestation in Indonesia's protected areas. *Conservation Letters*, *17*(3), e13010. https://doi.org/10.1111/conl.13010

Naidoo, R., Gerkey, D., Hole, D., Pfaff, A., Ellis, A. M., Golden, C. D., Herrera, D., Johnson, K., Mulligan, M., Ricketts, T. H., & Fisher, B. (2019). Evaluating the impacts of protected areas on human well-being across the developing world. *Science Advances*, *5*(4), eaav3006. https://doi.org/10.1126/sciadv.aav3006

Neal, T. (2024). Estimating the effectiveness of forest protection using regression discontinuity. *Journal of Environmental Economics and Management*, *127*, 103021. https://doi.org/10.1016/j.jeem.2024.103021
Neubert, S., McGowan, J., Metcalfe, K., Hanson, J. O., Buenafe, K. C. V., Dabalà, A., Dunn, D. C., Everett, J. D., Possingham, H. P., Stelzenmüller, V., Estep, A., Ervin, J., & Richardson, A. J. (2025). Multiple-use spatial planning for sustainable development and conservation. *Trends in Ecology & Evolution*, *0*(0). https://doi.org/10.1016/j.tree.2025.09.007
O'Garra, T., Martin, R., Pynegar, E., Polo-Urrea, C., & Eklund, J. (2025). Selecting among counterfactual methods to evaluate conservation interventions. *Conservation Science and Practice*, *n/a*(n/a), e70066. https://doi.org/10.1111/csp2.70066
Oldekop, J. A., Holmes, G., Harris, W. E., & Evans, K. L. (2016). A global assessment of the social and conservation outcomes of protected areas. *Conservation Biology*, *30*(1), 133–141. https://doi.org/10.1111/cobi.12568
Poudyal, M., Jones, J. P. G., Rakotonarivo, O. S., Hockley, N., Gibbons, J. M., Mandimbiniaina, R., Rasoamanana, A., Andrianantenaina, N. S., & Ramamonjisoa, B. S. (2018). Who bears the cost of forest conservation? *PeerJ*, *6*, e5106. https://doi.org/10.7717/peerj.5106
Rakotonarivo, O. S., Shyamsundar, P., Kramer, R., & Hockley, N. (2025). Conservation practice must catch up with commitments to local people for 30 × 30 success. *Nature Reviews Biodiversity*, *1*(2), 84–85. https://doi.org/10.1038/s44358-025-00021-4
Reusing, M. (1998). *Monitoring of Forest Resources in Ethiopia*. Ministry of Agriculture. http://localhost:8080/xmlui/handle/123456789/1055
Schleicher, J., Eklund, J., D. Barnes, M., Geldmann, J., Oldekop, J. A., & Jones, J. P. G. (2020). Statistical matching for conservation science. *Conservation Biology*, *34*(3), 538–549. https://doi.org/10.1111/cobi.13448
Schleicher, J., Zaehringer, J. G., Fastré, C., Vira, B., Visconti, P., & Sandbrook, C. (2019). Protecting half of the planet could directly affect over one billion people. *Nature Sustainability*, *2*(12), 1094–1096. https://doi.org/10.1038/s41893-019-0423-y
Van Zyl, H. (2015). *The Economic Value and Potential of Protected Areas in Ethiopia*. https://doi.org/10.13140/RG.2.1.2151.1449
Venter, O., Magrach, A., Outram, N., Klein, C. J., Possingham, H. P., Di Marco, M., & Watson, J. E. M. (2018). Bias in protected-area location and its effects on long-term aspirations of biodiversity conventions. *Conservation Biology*, *32*(1), 127–134. https://doi.org/10.1111/cobi.12970
Wakjira, M. T., Peleg, N., Six, J., & Molnar, P. (2024). Current and future cropland suitability for cereal production across the rainfed agricultural landscapes of Ethiopia. *Agricultural and Forest Meteorology*, *358*, 110262. https://doi.org/10.1016/j.agrformet.2024.110262
Waldron, A., Besancon, C., Watson, J. E. M., Adams, V. M., Sumaila, U. R., Garnett, S. T., Balmford, A., & Butchart, S. H. M. (2022). *The costs of global protected-area expansion (Target 3 of the post-2020 Global Biodiversity Framework) may fall more heavily on lower-income countries* (p. 2022.03.23.485429). bioRxiv. https://doi.org/10.1101/2022.03.23.485429
Wauchope, H. S., Jones, J. P. G., Geldmann, J., Simmons, B. I., Amano, T., Blanco, D. E., Fuller, R. A., Johnston, A., Langendoen, T., Mundkur, T., Nagy, S., & Sutherland, W. J. (2022). Protected areas have a mixed impact on waterbirds, but management helps. *Nature*, *605*(7908), 103–107. https://doi.org/10.1038/s41586-022-04617-0
Woodhouse, E., Bedelian, C., Barnes, P., Cruz-Garcia, G. S., Dawson, N., Gross-Camp, N., Homewood, K., Jones, J. P. G., Martin, A., Morgera, E., & Schreckenberg, K. (2022). Rethinking entrenched narratives about protected areas and human wellbeing in the Global South. *UCL Open Environment*, *4*, e050. https://doi.org/10.14324/111.444/ucloe.000050
Workie, T. G., & Debella, H. J. (2018). Climate change and its effects on vegetation phenology across ecoregions of Ethiopia. *Global Ecology and Conservation*, *13*, e00366. https://doi.org/10.1016/j.gecco.2017.e00366
World Bank. (2025). *Poverty and Inequity Platform* (Version 20250401_2021_01_02_PROD) [Dataset]. https://pip.worldbank.org/country-profiles/ETH
World Food Programme. (2025). *Ethiopia country strategic plan (2020-2025)*. https://www.wfp.org/operations/et02-ethiopia-country-strategic-plan-2020-2025
Wuepper, D., & Finger, R. (2023a). Regression discontinuity designs in agricultural and environmental economics. *European Review of Agricultural Economics*, *50*(1), 1–28. https://doi.org/10.1093/erae/jbac023
Wuepper, D., & Finger, R. (2023b). Regression discontinuity designs in agricultural and environmental economics. *European Review of Agricultural Economics*, *50*(1), 1–28. https://doi.org/10.1093/erae/jbac023

Zafra-Calvo, N., Garmendia, E., Pascual, U., Palomo, I., Gross-Camp, N., Brockington, D., Cortes-Vazquez, J.-A., Coolsaet, B., & Burgess, N. D. (2019). Progress toward Equitably Managed Protected Areas in Aichi Target 11: A Global Survey. *BioScience*, *69*(3), 191–197. https://doi.org/10.1093/biosci/biy143

Reviewer #1 (Remarks to the Author):

N/A the authors have answered my queries. I remain mildly concerned about the lack of political differentiation, but recognise the complexities of doing this in a country that is so ecologically and socially varied.

We are pleased that our response answered the reviewers queries.

Reviewer #2 (Remarks to the Author):

I find this to be a very carefully designed study with robust analytical choices. It is rare to see e.g. the sensitivity testing of arbitrary matching choices at the scale done here. I also find the replies to the previous round of reviewer comments convincing. The authors have embarked on a number of new analyses based on the comments by reviewers and they have fine-tuned their analyses and incorporated for example a consideration of funding/area and possible economies of scales of PA management costs. One of my previous concerns was the limited household data, but the authors show that the household data they have used is still a larger sample than similar data used in other studies. As far as I can see, this is a study that builds on the best data available and using state of the art matching to analyze it in a robust way.

The paper is now also more carefully framed and nuanced in its discussion of the findings and previous literature. The scope of this study is both ambitious and novel as it inspects PA performance from so diverse perspectives, most studies so far have focused on estimating either coverage/PA effectiveness in mitigating land-use change/ or PA impacts on local development. This study does all three and even adds stakeholder views, all within a carefully crafted study of a single case country which allows depth and use of the best available national data.

In conclusion, I can see that the authors have done a lot of hard work in addressing previous concerns by us reviewers and I am happy with this new version. I find the results extremely interesting and believe this will be a valuable and important contribution to the debate around the implementation of the 30by30 target.

We sincerely thank the reviewer for their careful and constructive engagement with the manuscript across both rounds of review. We are grateful for their positive assessment of the revised manuscript and for recognising the additional analyses and clarifications undertaken in response to reviewer feedback, and are pleased that the revised manuscript more clearly communicates the contribution of this work to ongoing debates around protected area effectiveness and the implementation of the 30-by-30 target.

Reviewer #3 (Remarks to the Author):

I thank the authors for their response to my comments. While the topic remains important, I maintain that the methodological limitations prevent the paper from producing trustworthy causal estimates – or at least with the level of certainty that the authors claim. Below I detail my remaining concerns. Unless the authors implement an alternative methodology to test for the internal validity of their results, I would recommend to reject the paper. Given the data available to the authors and

the context, a difference-in-differences model or a difference-in-discontinuity model seems appropriate.

Main methodological concern: identification strategy

My central concern remains the choice of statistical matching as the identification strategy. The authors state in their answers that they "fully agree [that matching] has limitations," yet they defend their approach without seriously exploring alternatives or reflecting these limitations in the main manuscript.

The authors rule out other approaches by assertion rather than demonstration. Other studies have successfully used instrumental variable approaches in similar contexts (e.g., Sims 2010; Libois et al. 2022). Here the authors simply state they cannot do so without explanation. Likewise for regression discontinuity design: the authors claim that conditions differ just inside and outside protected area boundaries, but do not show this. If the authors wish to convincingly rule out RDD, they should provide standard validation graphs demonstrating discontinuities in covariates at the boundary—this would support their claim that the approach is infeasible.

Instrumental variables

Instrumental variable approaches require a variable that (a) strongly predicts protected area designation and (b) is independent of the outcomes of interest except through protection designation. Our authorship team includes researchers and practitioners directly involved in protected area planning and implementation in Ethiopia, and we are unaware of any such variable.

In the Thailand case, Sims (2010) highlights that protecting upper watersheds was explicitly embedded in national protected area planning documents. This meant that watersheds supplying major river tributaries were much more likely to be designated as protected areas, allowing that feature to be used as an instrumental variable. Similarly, the strategy employed by Libois et al. (2022), exploits spatial diffusion of community forest programme rollout from district headquarters over time.

Unlike both cases mentioned above, protected area designation in Ethiopia has occurred through heterogeneous political, ecological, and administrative processes over multiple regimes with no consistent rule, prioritisation approach or systematic spatial rollout. Without a credible instrumental variable, implementing an instrumental variables approach would risk introducing greater bias than the matching design. We therefore adopt a transparent quasi-experimental matching framework, accompanied by extensive sensitivity analysis to assess robustness to unobserved confounding, and demonstrate that an unobserved confounder would need to be substantially stronger than the most influential observed covariates to overturn our conclusion.

Regression discontinuity

A standard regression discontinuity design is appropriate when treatment assignment is determined by a clearly defined threshold on a continuous eligibility variable (O'Garra, 2025). In our case, protected areas in Ethiopia were not designated based on any measurable threshold rule. Instead, designation occurred through heterogeneous political, ecological and administrative processes over several decades. As a result, there is no continuous variable and cut-off point that would allow implementation of a conventional regression discontinuity design.

We recognise that spatial/geographical regression discontinuity designs (RDD)can sometimes be applied using distance to a treatment boundary as the running variable, such as in Bonilla-Mejíaa &

Higuera-Mendieta (2019). However, this approach relies on different but equally strong assumptions: that the only discontinuity at the boundary must be treatment assignment (Keele and Titiunik, 2015). In Ethiopia, this assumption is unlikely to hold. Protected areas boundaries often follow ecological features, traditional land use changes or administrative boundaries and are decided based on a wide range of political, geographical, socio-economic and ecological characteristics. These factors are likely to create real differences at the boundary unrelated to the casual effect of protection making it difficult to isolate the treatment effect.

Spatial RDD also estimates a local boundary effect, not the average effect across entire protected areas. For example, Bonilla-Mejíaa & Higuera-Mendieta (2019) apply spatial RDD in Colombia and, using data-driven optimal bandwidths, estimate effects using strips as narrow as 2.8 km either side of protected area boundaries. Their estimates therefore reflect outcomes for land located close to PA borders, rather than impacts in core interior areas. In our case, many protected areas are large, and pressures, enforcement, and ecological conditions vary substantially between boundary zones and interior areas. A spatial RDD would therefore identify a local edge effect, whereas our objective is to estimate the average treatment effect on the treated (ATT) across the protected area system as a whole. This creates a mismatch between what spatial RDD is designed to estimate and the policy-relevant parameter we seek to identify.

The spatial configuration of Ethiopia's protected area network further limits the feasibility of a spatial RDD. As illustrated in Figure 1, many protected areas are adjoining or form clusters. Spatial RDD requires a clean comparison between treated and untreated units at a boundary. In our case, however, land just outside one protected area is often part of another protected area, buffer zone, or alternative conservation designation. In cases such as this, spatial RDD requires restricting the analysis to "valid" boundary segments that separate protected from genuinely unprotected land. Bonilla-Mejía and Higuera-Mendieta (2019), for example, implement a two-step correction in which they exclude boundary segments adjacent to other protected areas and recompute distances relative only to valid borders. While this approach mitigates compound treatment bias, it substantially reduces the effective sample and further localises the estimand to specific boundary segments. Given the clustering of protected areas in Ethiopia, applying such restrictions would exclude a large proportion of PA perimeters.

In contrast, our matching-based design does not rely on local exchangeability at a boundary. Instead, it explicitly balances observed environmental, geographic, and socio-demographic covariates across treated and control units. While matching requires the assumption of no important unobserved confounders, this assumption is transparent and testable through balance diagnostics and sensitivity analyses. Given the complex way protected areas were established in Ethiopia and our focus on system-wide impacts rather than edge effects, we consider the assumptions required for matching to be more appropriate, and likely less violated, than those required for spatial regression discontinuity designs.

Bonilla-Mejía, L., & Higuera-Mendieta, I. (2019). Protected areas under weak institutions: Evidence from Colombia. *World Development*, *122*, 585-596.

Keele, L. J., & Titiunik, R. (2015). Geographic boundaries as regression discontinuities. *Political Analysis*, *23*(1), 127-155.

O'Garra, T., Martin, R., Pynegar, E., Polo-Urrea, C., & Eklund, J. (2025). Selecting among counterfactual methods to evaluate conservation interventions. *Conservation Science and Practice*, *7*(11), e70066.

When defending matching over BACI / Difference in differences, the authors fail to recognize the fundamental trade-off between internal and external validity. Matching relies on the assumption that selection into treatment is fully captured by observable covariates—an assumption difficult to defend in the context of PA placement, where unobserved political, ecological, and accessibility factors likely drive both placement and outcomes. Other methods, such as BACI allow to drastically limit the number of assumptions to made. It comes at a cost: focusing on protected areas created over the pat 20 years. The authors choose a method with questionable internal validity in order to generalize results. But generalizing results from a method that lacks internal validity simply means generalizing untrustworthy estimates.

We agree that there is an important trade-off between internal and external validity when selecting among quasi-experimental approaches. Difference-in-differences (DiD) designs can strengthen internal validity by exploiting within-unit temporal variation and relaxing reliance on selection-on-observables. However, in the Ethiopian context, the majority of protected areas were established prior to the availability of consistent remote sensing and socioeconomic baseline data. A DiD design would therefore necessarily restrict the analysis to the small subset of protected areas designated after 2000, substantially limiting the scope of inference and reducing policy relevance for assessing system-level effectiveness.

Our objective is to evaluate the performance of the national protected area network as implemented. To do so, we prioritised an approach that allows inclusion of the full set of protected areas, while carefully addressing observable sources of selection bias through matching and extensive sensitivity analyses. We acknowledge that matching relies on the selection-on-observables assumption, and we therefore conduct robustness checks to assess how strong unobserved confounding would need to be to overturn our conclusions. These tests indicate that an implausibly large unobserved confounder would be required.

In this context, we consider the trade-off transparent and deliberate: a DiD design would increase internal validity for a small and potentially unrepresentative subset of sites, whereas our matching approach allows broader system-level inference while making its identifying assumptions explicit and testable.

We have added to the discussion:

> "Our estimates therefore rely on the assumption that, conditional on the matched covariates, treated and control units would have followed similar trajectories in the absence of protection. Violation of this assumption could bias estimates; however, sensitivity analyses indicate that an unobserved confounder would need to be implausibly stronger than the most influential observed covariates to overturn our conclusions. Alternative quasi-experimental approaches[89] could in principle strengthen internal validity but would substantially restrict the scope of inference. For example, a difference-in-differences design would require protected areas established after 2000 accounting for only around one quarter of Ethiopia's protected areas. Restricting the analysis to this subset would not only reduce sample size but would focus on newer, often smaller protected areas that are unlikely to be representative of the national system. Such an approach would therefore shift the estimand

away from the performance of Ethiopia's protected area network as currently implemented which is central to national planning under the Global Biodiversity Framework. Given Ethiopia's need to balance conservation targets with development priorities at the national scale, we therefore retain a system-wide assessment while transparently acknowledging and empirically testing the assumptions required by the matching design."

Interpretation of impact evaluation results

Despite acknowledging matching's limitations in their response, the authors' results still read as definitive causal claims. For example, they state that "Protected areas *reduced* forest cover loss by *25%* compared to control." This language implies far more certainty than the methodology can support in terms of causality and the precision of estimates - particularly given that point estimates in Figure 3 vary by a factor of three across specifications. This concern is compounded by the authors' tendency to present robustness tests selectively: claiming that 97% of specifications yield results in the same direction, while failing to acknowledge the substantial variation in point estimates—variation that should temper confidence in any single estimate. The same critic applies across the entire section.

We have added more in-depth discussion of the limitations of matching (see response to previous comment).

To address the reviewer's comments, we make two points. First, the matching specification used in the main analysis was selected because it achieved the best covariate balance while retaining a high proportion of treated units, and therefore provides the most credible estimate of the treatment effect. Second, and nevertheless, we present results from a wide range of alternative specifications to show that our overall conclusions (i.e. the directionality of the results, showing PAs are effective) are robust to a very wide variety of modelling choices which we include to be transparent and reassure readers that specific model choice is not driving the main message of the manuscript.

The section on trade-offs between environmental and social outcomes assumes that LSMS data are representative of wellbeing at the PA level. However, LSMS surveys are designed to be nationally representative, not locally representative of PA-adjacent populations. Yet you are computing an average treatment effect by Protected Area to make this analysis hence assuming that the households in LSMS are representative of the population of this specific area.

For the individual protected area results we have added: "For wellbeing outcome ATTs, the treated group comprises LSMS-sampled households located within 10km of that protected area and is not necessarily representative of protected-area level population estimates".

Additionally, the analysis explaining heterogeneity in PA-level effects uses estimated ATTs as the dependent variable. Because these ATTs are estimated with considerable noise (as Figure 3 demonstrates), using point estimates without accounting for their uncertainty is problematic. This is an advanced generated regressors problem: errors propagate from the first to the second stage ; and the error in the first model is quite large according to Figure 3. The current approach does not account for this, yielding incorrect standard errors and probably effect sizes.

Our intention with this secondary analysis is exploratory: to examine broad patterns in how environmental and wellbeing outcomes vary across protected areas and to identify potential

correlates of better or worse performance. We intentionally do not make causal claims about the determinants of heterogeneity. Additionally, the core conclusions of the manuscript do not depend on statistical inference from these second-stage models, but rather on the overall distribution of environmental and social outcomes and the prevalence of trade-offs across protected areas which are evident from the results of the primary analysis.

To clarify this, we have revised the manuscript to explicitly frame these analyses as exploratory and to note that uncertainty in first-stage individual protected area ATT estimates is not fully propagated into second-stage models. We have also adjusted language throughout the second-stage model results (e.g., replacing "predictors" with "associations") to avoid implying causal interpretation of these results.

We hope this clarification addresses the reviewer's concern while preserving the value of this analysis in identifying broad patterns in protected area performance across the system.

Missing studies in R3.01
"1. Environmental and wellbeing outcomes are rarely assessed together"
Some key missing studies in your answer attenuate the novelty of your claim:

- Sims, K. R. (2010). Conservation and development: Evidence from Thai protected areas. Journal of environmental economics and management, 60(2), 94-114.

- Libois, F., Baland, J. M., Delbart, N., & Pattanayak, S. (2022). Community Forest Management: The story behind a success story in Nepal.

- Alix-Garcia, J. M., Sims, K. R., & Yañez-Pagans, P. (2015). Only one tree from each seed? Environmental effectiveness and poverty alleviation in Mexico's payments for ecosystem services program. American Economic Journal: Economic Policy, 7(4), 1-40.

- Sims, K. R., & Alix-Garcia, J. M. (2017). Parks versus PES: Evaluating direct and incentive-based land conservation in Mexico. Journal of Environmental Economics and Management, 86, 8-28.

« 2. Social outcomes are rarely assessed at the household level"
I don't find this statement and answer completely convincing as your answer miss several relevant studies (well-pubished & well-cited , not obscure papers). This includes:

- Naughton-Treves, L., Alix-Garcia, J., & Chapman, C. A. (2011). Lessons about parks and poverty from a decade of forest loss and economic growth around Kibale National Park, Uganda. Proceedings of the National Academy of Sciences, 108(34), 13919-13924.
- Nowakowski, A. J., Canty, S. W., Bennett, N. J., Cox, C. E., Valdivia, A., Deichmann, J. L., ... & McField, M. (2023). Co-benefits of marine protected areas for nature and people. Nature Sustainability, 6(10), 1210-1218.
- Desbureaux, S., Girard, J., Dalongeville, A., Devillers, R., Mouillot, D., Jiddawi, N., ... & Leblois, A. (2024). The long-term impacts of Marine Protected Areas on fish catch and socioeconomic development in Tanzania. Conservation Letters, 17(6), e13048.

We thank the reviewer for highlighting these studies. We have included the three most relevant studies in the manuscript: Sims (2010), Sims & Alix-Garcia (2017), and Naughton-Treves et al (2011). Where we discuss studies that have assessed both environmental and wellbeing outcomes in the introduction of the manuscript, we are specifically referring to those assessing terrestrial protected area impacts. Libois et al (2022) assess community forest management programmes rather than protected areas, and additionally they do not causally assess social outcomes of the programme, instead they use household data to investigate potential mechanisms underlying forest improvements. Alix-Garcia et al (2015) assess the impacts of a conditional cash transfer programme rather than protected areas. Both Nowakowski et al. (2023) and Desbureaux et al (2024) assess marine protected areas.

Reviewer #4 (Remarks to the Author):

I enjoyed reading the manuscript, which aims to evaluate whether Ethiopia could expand its protected areas, which currently cover about 10% of the country, to achieve the Global Biodiversity Framework's 30-by-30 target, excluding Forest Priority Areas. Generally, the manuscript is well designed and well written, covering large topics. It explores the conservation challenges facing developing countries, particularly those affecting local livelihoods and protected areas. This will help design policy and management strategies for the short and long term. I recommend the manuscript as a minor review. I have a few minor and major comments below to help improve the quality of the manuscript.

We thank the reviewer for their thoughtful and encouraging assessment of the manuscript. We are pleased that they find the study well designed, clearly written, and policy relevant, and that they recognise its contribution to understanding conservation challenges in lower-income country contexts, particularly in relation to livelihoods and protected area expansion under the Global Biodiversity Framework's 30-by-30 target.

Abstract:
Line 43: Does it also help species conservation or….

We assume that by maintaining natural habitats and reducing agricultural expansion within protected area boundaries, that the sites are helping species conservation but we did not assess this specifically due to data limitations.

Introduction
The Introduction is well framed and written from general to specific, with clear background information.
Line 79: … does the only concern is …. "local food supplies"…
Probably a local livelihood in which local communities not only depend on agriculture but also on cattle grazing, the use of forest resources, etc

We presume the reviewer means line 48 where we mention "local food supplies" we have added "local livelihoods" to this sentence.

Results

Line 150-158: Good to clarify more the number of threatened species in each group compared to the total species number in each category, such as mammals, birds, amphibians, reptiles and plants. Fig. 2b does not clearly show the details as it only shows the proportions. How about the endemic animal species representation in protected areas? Good to mention in the text.

We believe there may be some confusion over the line numbers but in the paragraph related to species representation we have now added the number of species in each category to both the text and figure legend.

Line 186-189: See the comment in the abstract above. The household income of local people living in the vicinity of protected areas is affected. Their livelihoods depend on agriculture, domestic animal grazing, and firewood collection, but they lack access to these resources in strictly protected areas. The competition for resources beyond food.

We are not sure what the reviewer is referring to here as the line numbers don't match up, we agree with the statement that protected areas have an impact beyond food and have assessed outcomes beyond food looking at material wellbeing measured as an asset ownership index.

Discussion

Line 289-281: How about the long-term solution of expanding protected areas? In the future, industrial transformation, such as that in China, will likely increase urbanization and reduce the number of people living near protected areas, which leaves more land for protected area expansion. However, such a transformation would likely be supported by developed nations as part of climate change adaptation and capacity building. Good to think about short-term and long-term solutions.

It would be great to summarize the main findings of your study in one concluding paragraph or two, including implications for conservation, policy and local livelihood development for the short-term and long-term.

We have added a sentence to the discussion: "While economic transformation and urbanisation may reduce dependence on land-based livelihoods and help ease conservation-livelihood tension over time, future progress towards a more representative network will require…"

Materials and Methods:

The Materials and Methods section is detailed, which is helpful for researchers who could carry out similar studies. However, it is long in its current form and not suitable for general readers. I suggest the authors should reduce it by about 1/4th. It is not necessary to explain the details, and it would be good to move part of it to the supplementary file.

Due to the large scope of analyses undertaken we have maintained the methods to ensure reproducibility for readers.

Line 386-388: Please double-check the number of amphibian and reptile species found in Ethiopia. The number of amphibian and reptile species appears lower than that found in Ethiopia. Please double-check recent studies. It would be great to mention the number of threatened animal and plant species in the text. Line 896: Double check whether it is … 200 or ….2000 …

The number used in this study is not the total number of amphibian and reptile species found in Ethiopia, it is the number with range data available in the IUCN Red List. This is made clear in the

methods but we have now updated the results section to say “across Red Listed species” rather than just “across species”.

***

Additional comments from Reviewer 2 in response to Reviewer 3's comments:

Regarding the reviewer’s comments, I believe the suggestions are technically very interesting but, in this particular case, perhaps a bit unreasonable. In an ideal world with unlimited data, a difference-in-differences (DiD) approach would indeed be preferable to simple matching. The before-after component would increase internal validity by strengthening the evidence that observed differences in outcomes are due to the intervention rather than pre-existing differences. However, the challenge here, as in most protected area (PA) studies, is that the majority of PAs were established long before remote sensing data on forest loss became available. This means that a DiD design would be limited to PAs established after 2000. Based on my reading of the Ethiopia case, such an analysis could only be conducted for 16 out of the 79 PAs, severely limiting the scope of the study and its policy relevance. Rather than strictly requesting this addition or completely reframing the paper, I feel this is an interesting research question that would merit a separate study.

As for the suggestion to use a regression discontinuity design (RDD), I am somewhat confused. My understanding is that RDD is most useful when an eligibility criterion can serve as a proxy or cut-off point for who or what receives an intervention. The reviewer writes that “the authors claim that conditions differ just inside and outside protected area boundaries, but do not show this.” However, I fail to see why such an approach would be necessary here, as the PA polygons themselves are the intervention. Hence, since spatial data on the intervention is available, why revert to a proxy when the actual delineation is already known?

The instrumental variable approach is an interesting idea, but it is rarely used in PA effectiveness studies. I would be keen to hear what instrumental variable the reviewer suggests that could work in the Ethiopian context. The reviewer refers to studies in Thailand that used priority watershed status and another study on community forest management in Nepal (though I could only find working paper versions of the latter, so just wanted to flag that it may not have undergone peer review). Again, I find myself asking why such an approach would be preferable to using the spatially delineated PA boundaries, which, by definition, represent the intervention.

In sum, I believe that a study design based on matching is the best option given the data limitations (i.e., forest loss satellite data being available only from 2000 onwards). While a DiD design would be ideal, it would severely restrict the scope to only the 16 most recently established PAs out of 79. I therefore find it unreasonable to demand a different methodology and reject the paper based on this. Instead, in my opinion, it is enough that the authors stress that matching is sensitive to unobserved confounders. Note also that the authors have done some proper sensitivity analyses and show how much an unobserved variable would need to explain to be relevant. I think the following two papers can be used justify why matching in this case is ok (albeit not ideal):

- O'Garra, T., Mangubhai, S., Jagadish, A., Tabunakawai-Vakalalabure, M., Tawake, A., Govan, H., & Mills, M. (2023). National-level evaluation of a community-based marine management initiative. Nature Sustainability, 6, 908–918.
- O'Garra (2025). Selecting among counterfactual methods to evaluate conservation interventions. Conservation Science and Practice, 7, e70066.

We thank Reviewer 2 for this thoughtful and constructive clarification of the methodological trade-offs involved in evaluating protected area effectiveness at national scale. We have revised the manuscript to more explicitly acknowledge this trade-off between internal and external validity and to clarify the rationale for our chosen quasi-experimental design.

Following the reviewer's helpful suggestions, we have also strengthened the discussion of the assumptions and limitations of matching approaches and have cited the recommended references (O'Garra et al. 2023; O'Garra 2025) to situate our methodological choices within the broader conservation evaluation literature. We are grateful for these suggestions, which have helped improve the clarity and balance of the manuscript.
